# Overcoming Back Electron Transfer in the Electron Donor–Acceptor Complex-Mediated Visible Light-Driven Generation of α-Aminoalkyl Radicals from Secondary Anilines

**DOI:** 10.1021/acs.joc.2c02448

**Published:** 2022-12-08

**Authors:** August Runemark, Henrik Sundén

**Affiliations:** † Department of Chemistry and Chemical Engineering, Chalmers University of Technology, Kemivägen 10, Gothenburg 412 96, Sweden; ‡ Chemistry and Molecular Biology, University of Gothenburg, Kemivägen 10, Gothenburg 412 96, Sweden

## Abstract

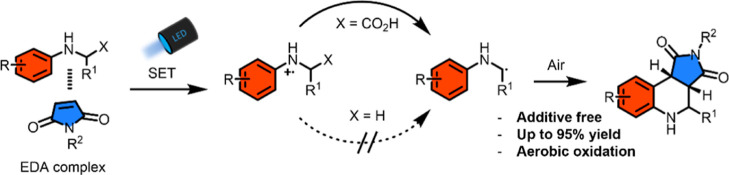

An additive-free, visible light-driven annulation between *N*-aryl amino acids and maleimide to form tetrahydroquinolines (THQs) is disclosed. Photochemical activation of an electron donor–acceptor (EDA) complex between amino acids and maleimides drives the reaction, and aerobic oxygen acts as the terminal oxidant in the net oxidative process. A range of *N*-aryl amino acids and maleimides have been investigated as substrates to furnish the target THQ in good to excellent yield. Mechanistic investigations, including titration and UV–vis studies, demonstrate the key role of the EDA complex as the photoactive species.

## Introduction

α-Functionalization of amines is an invaluable synthetic method and has been used frequently in the synthesis of complex molecular structures, natural products, and biologically active compounds.^1−3^ Recently, radical approaches to α-functionalization have received widespread attention due to the development of photoredox catalysis, which has realized a range of visible light-driven methods.^3−6^ Typically, a photoredox catalyst in its excited state acts as an electron acceptor to oxidize the amine via a single electron transfer (SET). The formed amine radical cation can then undergo α-deprotonation to form a reactive alkyl radical that can be reacted with a suitable radical acceptor. Alternatively, the α-aminoalkyl radical can be further oxidized to form the iminium ion and coupled with a nucleophilic species. However, the use of photoredox catalysts can be troublesome and typically relies on expensive and rare transition metals or complex organic dyes. Separation and recycling of the catalyst pose another major problem.^7^ A way to circumvent the use of a catalyst is to take advantage of an electron donor–acceptor (EDA) complex. An EDA complex is a weak molecular aggregate that can form between a nucleophilic donor and an electrophilic acceptor.^8,9^ Associated with the formed complex is the emergence of a new electronic transition in the electromagnetic spectrum, absent in either the donor or acceptor. Excitation of the complex at this energy induces a net SET from the donor to the acceptor.^9,10^ The radicals formed after the SET can subsequently be used in a range of reactions.^8,11−17^ The fact that the new electronic transition is of lower energy than needed to excite any of the individual species separately very often results in that visible light can be used to excite the EDA complex which is attractive as UV light can be circumvented.

**Scheme 1:**
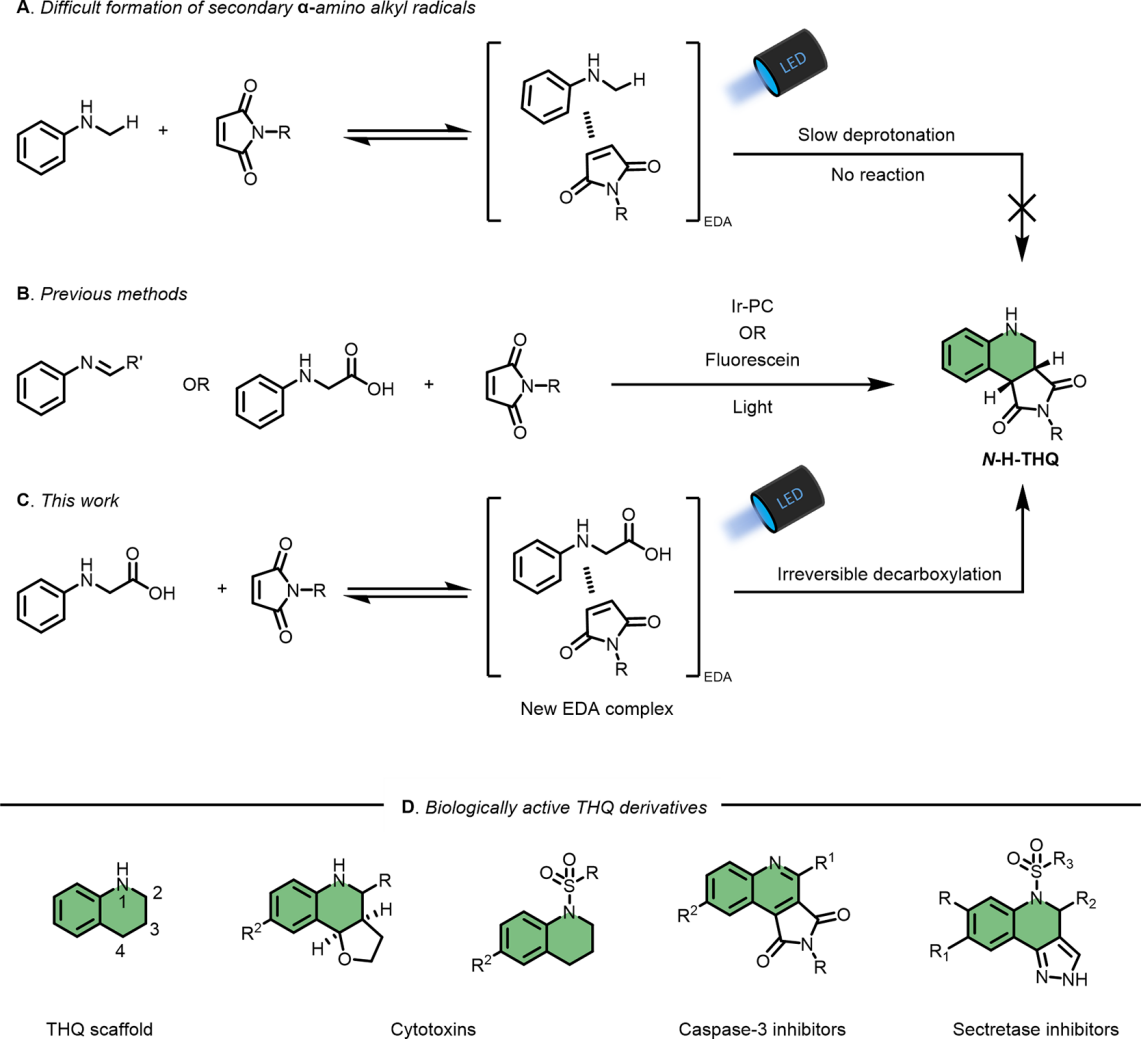
Challenges Associated with the Synthesis of *N*-H–THQs and Biologically Important Targets That Can be Derived from the *N*-H–THQ Core Structure (A) General principle of the generation of secondary α-aminoalkyl radicals from *N*-alkyl anilines; (B) previous methodologies for the synthesis of *N*-H–THQ; (C) this work; and (D) numbering of the THQ scaffold and examples of biologically active THQ and quinolines.

The use of amines as potent electron donors in EDA complexes has been widely documented.^8,11−13^ Recently, we have shown that tertiary anilines can act as donors in EDA complexes in combination with activated alkenes for the visible light-driven synthesis of tetrahydroquinolines (THQs), circumventing the use of complex and expensive photoredox catalysts.^18−20^ However, whereas this catalyst-free approach works well for *N*,*N*-dialkylated anilines, secondary anilines have not been suitable substrates most likely due to back electron transfer (BET). Thus, a major challenge for applications of EDA complexes in organic synthesis is to overcome the BET that can occur after the photoinitiated SET, suppressing the formation of radical ions.^11,21^ This is especially prominent for the generation of secondary α-aminoalkyl radicals as the BET from the amine radical cation back to the oxidant is faster than any forward process (Scheme 1A).^22^ Therefore, developing ways of achieving α-functionalization of secondary anilines is highly desirable.

Different approaches have been developed to circumvent this problem.^22^ One strategy relies on installing a good leaving group in the α-position of the aniline which, after the SET, results in the rapid fragmentation of the substrate, driving the reaction forward. Examples include silyl and carboxyl groups. Several reports of the use of *N*-aryl glycines as the α-aminoalkyl radical precursor in combination with photocatalysts have been reported in the literature.^23−49^ Previous methods providing α-aminoalkyl radicals from amino acids, using an EDA complex approach, include the use of activated esters as acceptors.^14,50,51^

Generation of secondary α-aminoalkyl radicals and reacting them with alkenes would potentially give access to *N*-H–THQ (Scheme 1A), a useful core structure that can be used for the synthesis of a range of biologically active compounds including cytotoxins, γ-sectretase inhibitors, and caspase inhibitors (Scheme 1D).^52−55^*N*-H–THQs are typically accessible via Povarov-type reactions between imines and electron-rich alkenes.^56^ However, synthesis of *N*-H–THQs bearing electron-withdrawing groups is limited to reverse polarity cycloadditions catalyzed by iridium complexes or via deprotection of the *N*-Bn–THQ derivative (Scheme 1B).^57−59^

Inspired by our earlier findings on the synthetic power of aniline–alkene EDA complexes, we postulated that an *N*-aryl amino acid could serve as an electron donor in combination with an activated alkene as the electron acceptor.^20,60^ A photoinitiated charge transfer would lead to an α-carboxyl amine radical cation that could fragment irreversibly, via decarboxylation, to give an α-amino radical.^61^ Herein, we present a novel synthesis where photo-generated secondary α-amino radicals react with maleimides under aerobic conditions to form *N*-H–THQs (Scheme 1C).

## Results and Discussion

**Figure 1:**
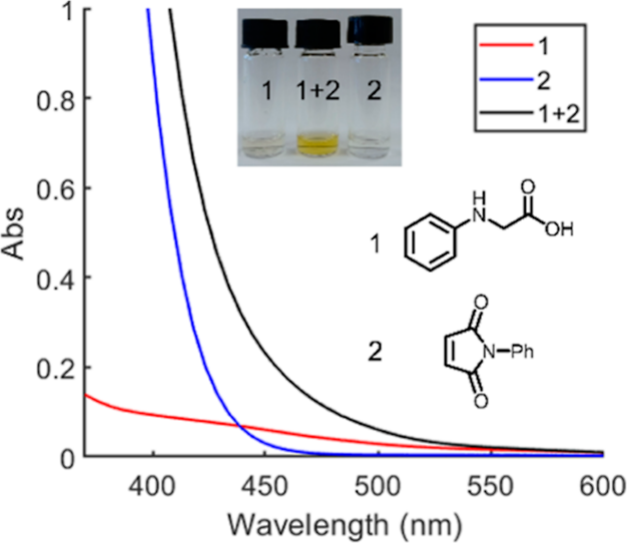
Indication of formation of an EDA complex between **1** and **2**.

To initiate the study, a model system based on *N*-phenyl glycine (**1**) and *N*-phenyl maleimide (**2**) was chosen. When mixed in solution, an increased light absorption in the visible range compared to that of the individual species was observed (Figure 1). This result, indicating the formation of an EDA complex between the two reactants, prompted the investigation of the photoproducts upon irradiation.

A solvent screen revealed that methanol with water as the co-solvent and a 440 nm light-emitting diode (LED) as the light source gave the best reaction outcome in terms of yield and purity of the product. Under our optimal conditions, the desired product **3** was formed in 95% yield after 3 h irradiation time (Table 1, entry 1).

**Table 1:**
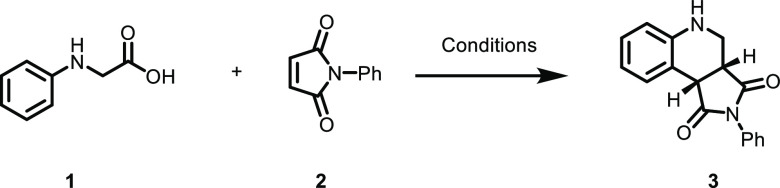
Effect of Reaction Conditions

entry	deviation from standard conditions^a^	yield 3^b^
1	None	95 (90^c^)
2	EtOAc as the solvent	15
3	ACN as the solvent	52
4	1,4-dioxane as the solvent	85
5	MeOH as the solvent	90
6	DMSO as the solvent	41
7	green LED (525 nm), 3 h	37
8	green LED (525 nm), 18 h	90
9	under an O_2_ atmosphere	96
10	under a N_2_ atmosphere	3
11	in the dark	0
12	UV–CFL (370 nm), 3 h	34

a Standard conditions: **1** (0.1 mmol), **2** (4 equiv) in MeOH/H_2_O (2:1). 3 mL was irradiated for 3 h using a 440 nm 40 W LED under an ambient atmosphere.

b Determined by GC–FID using chlorobenzene as the internal standard.

c Isolated yield.

Omitting the addition of water, using pure methanol as the solvent, decreased the yield of the reaction slightly, whereas other polar aprotic solvents such as acetonitrile, 1,4-dioxane, or dimethyl sulfoxide (DMSO) resulted in significantly lower yields (Table 1, entries 3 and 4). These results are in line with the literature, and the beneficial impact of water on the decarboxylation of *N*-aryl glycines has been observed previously.^23,48,62^ Less polar solvents, like ethyl acetate, were not suitable as reaction medium (Table 1, entry 2), and other non-polar solvents could not dissolve the reactants, suppressing the reaction completely. The absorption of the reaction mixture in different solvents was carried out (Supporting Information), and it was observed that methanol and methanol–water resulted in the most red-shifted absorption, compared to less polar systems. Using a green LED (525 nm) resulted in a lower reaction rate with only 37% yield after 3 h (Table 1, entry 7). Increasing the reaction time to 18 h, however, resulted in comparable yields as those with the blue LED (Table 1, entry 8). The lower reaction rate can be related to the significantly lower absorption of the reaction mixture above 500 nm compared to that above 440 nm, see Figure 1. If run under an atmosphere of oxygen, the reaction proceeds smoothly, whereas the exclusion of the oxidant results in diminished conversion (Table 1, entries 9 and 10), showing that oxygen has an important role in this transformation. For discussion on the mechanism, see below.

**Scheme 2:**
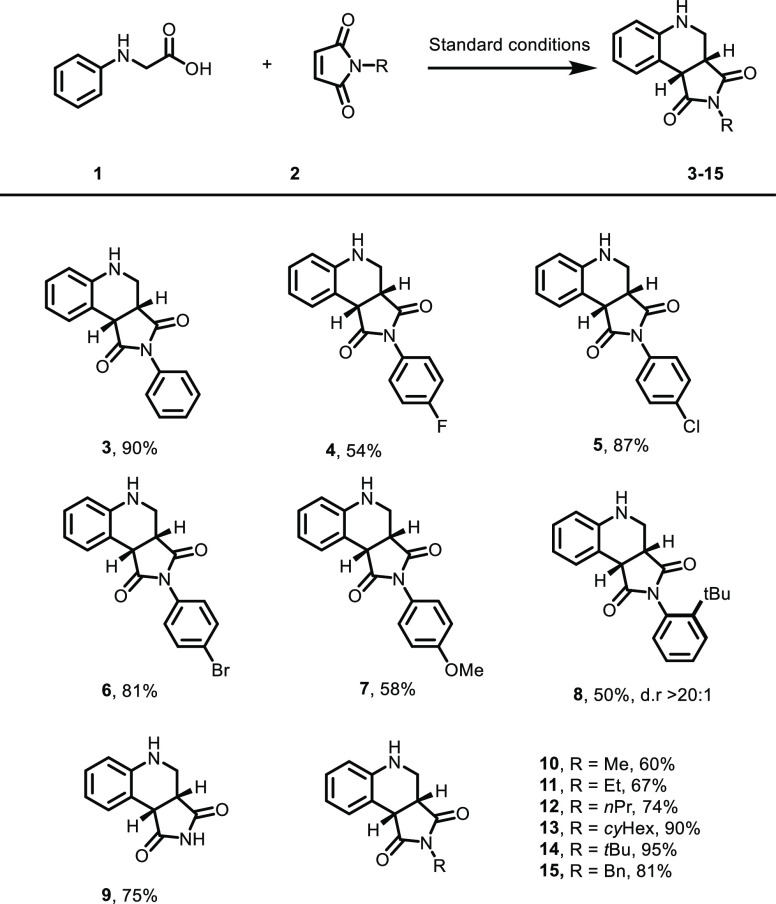
Scope of Maleimides Conditions: substituted maleimide (0.1 mmol) and **1** (4 equiv) in MeOH/H_2_O (2:1). 3–4 mL was irradiated for 3–6 h using a 440 nm 40 W LED under an ambient atmosphere. For full conditions, see the Experimental Section part.

With our optimal reaction conditions developed, different maleimides as acceptors in the reaction were investigated (Scheme 2). Different *N*-aryl maleimides were generally well tolerated in the reaction, leading to the products **3–14** in moderate to high yields. Electron-withdrawing halogens in the *para* position resulted in higher yields than the electron-rich *p*-OMe substituent (Scheme 2, **4–7**). Installation of the bulky *t*-Bu group in the *ortho* position of the *N*-phenyl maleimide decreased the yield significantly to 50% (Scheme 2, entry **8**). The product **8** was obtained in an excellent diastereomeric ratio due to the steric hindrance and restricted rotation.

Next, different *N*-alkyl-substituted maleimides were investigated as substrates in the reaction leading to products **10–14** in 60–95% yield. For example, the bulky *N*-cyclohexyl maleimide is a potent substrate in this transformation, and the corresponding *N*-H–THQ **19** could be isolated in 90% yield indicating that bulky groups on the maleimide do not interfere with the formation of the EDA complex. Unsubstituted maleimide was also a suitable substrate in the reaction leading to the product **9** in 75% yield.

**Scheme 3:**
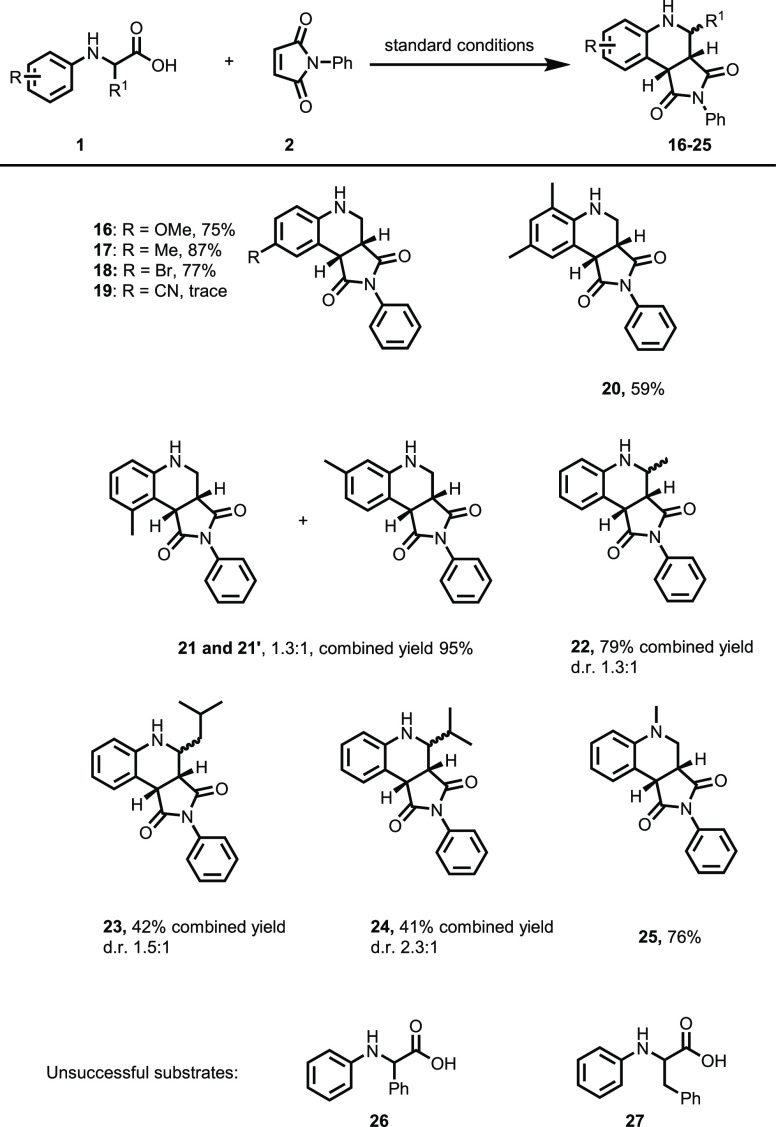
Scope of *N*-Aryl Amino Acids Conditions: **2** (0.1 mmol), amino acid derivative (4 equiv) in MeOH/H_2_O (2:1). 3–4 mL was irradiated for 3–6 h using a 440 nm 40 W LED under an ambient atmosphere. For full conditions, see the Experimental Section part.

The aromatic substitution on the amine reaction partner was proven to have a significant impact on the reaction outcome (Scheme 3). Electron-donating groups such as methyl or mildly withdrawing groups such as bromide were tolerated giving the products **17–18** in 77–87% yield. However, the strongly electron-withdrawing cyano group resulted in only a trace amount of the product formed (Scheme 3, entry **19**). Installation of a methyl group in the *ortho* position of the amine resulted in a significantly lower yield, and product **20** could be isolated in 59% yield as compared to compound **17** isolated in 87% yield lacking the *ortho*-methyl substitution. This might be due to the less planar *o*-substituted aniline interfering with the EDA complex formation. Installation of a methyl group in the less sterically demanding *meta* position did not affect the yield but gave a mixture of regioisomers **21** and **2′** in a 1.3:1 ratio.

Introduction of α-substituents of the *N*-aryl glycine reaction partner led to the formation of products **22–24** (Scheme 3). Generally, the yield was lower compared to that of unsubstituted **1**, presumably due to the increased steric hindrance, and the products were obtained as mixtures of diastereomers. When *N*-phenyl-*N*-methyl glycine was used as the reactant, product **25** was observed as the only product, indicating that the decarboxylation is significantly faster than α-deprotonation of the amine radical cation. The benzyl- and phenyl-substituted **26** and **27** were also tried as substrates in the reaction; however, in these cases, the amine decomposed upon exposure to the reaction conditions, and no desired product could be obtained.

**Scheme 4:**
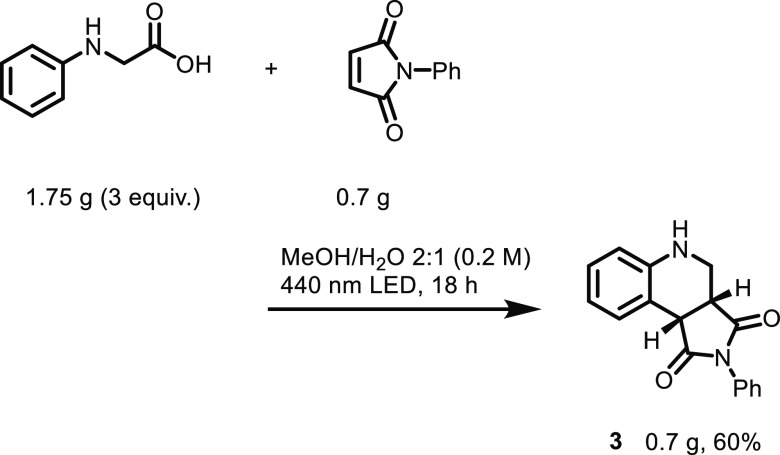
Scale Up of the Annulation Reaction

To show the synthetic potential of the reported method, a reaction was performed on the gram scale to provide the desired product **3** in a yield of 60% (Scheme 4).

**Scheme 5:**
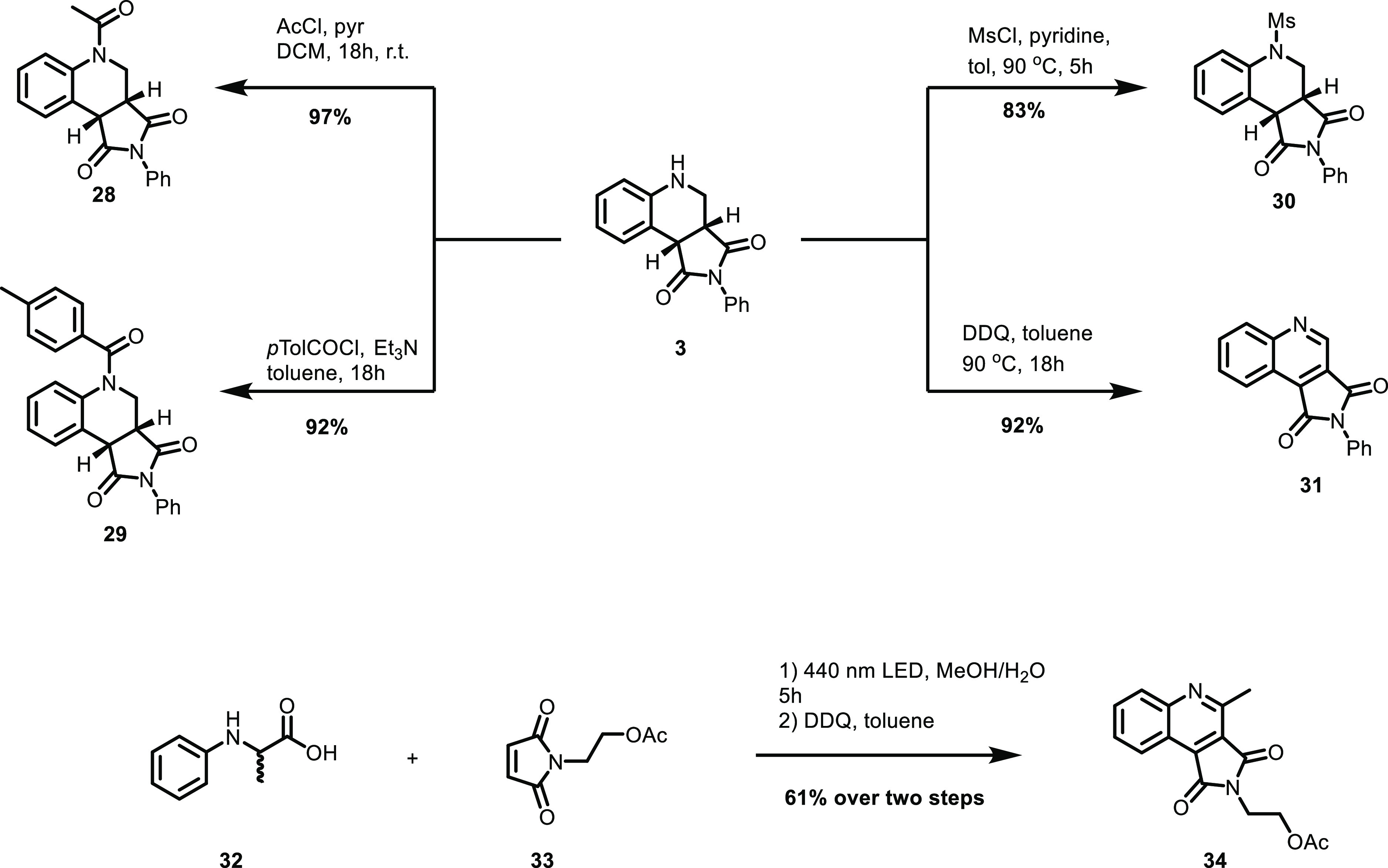
Derivatization of the Model Product **3** and Methodology Application

The formed product could then easily be transformed to different derivatives (Scheme 5). The *N*-acylated products **28** and **29** could be obtained in high yields by treating **3** with acyl or toluoyl chloride under basic conditions. The corresponding *N*-mesyl derivative **30** could also easily be furnished. Under oxidative conditions, **3** could be transformed to the quinoline analogue **31** in a facile manner.

To further exemplify the usefulness of the developed protocol, quinoline **34** was synthesized in 61% yield in a two-step procedure from amino acid **32** and maleimide **33**. Quinolines with the core structure of **34**, that has been shown to be active as potent caspase-3 inhibitors, are typically synthesized from isatins using a multistep procedure.^53−55^ The present protocol showcases the possibilities for a fast and facile synthesis of a large library of the compound class.

**Scheme 6:**
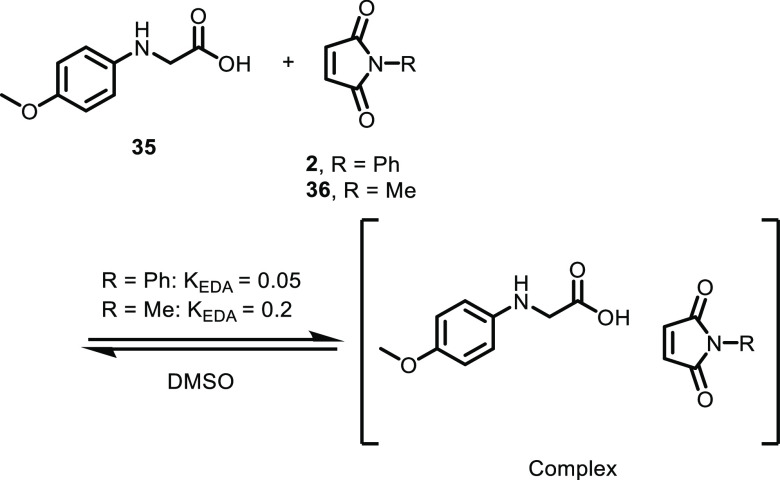
Determination of the Association Constant of the EDA Complex

To elucidate the mechanism of the reaction, a series of control experiments were conducted. First, the necessity of oxygen for a successful reaction outcome was demonstrated as only trace amounts of the product could be observed when the reaction was run under an atmosphere of nitrogen (Table 1, entry 10). Light irradiation was also shown to be needed to drive the reaction (Table 1, entry 11). As demonstrated in Figure 1, the formation of a red-shifted absorbance in the UV–vis spectrum upon mixing of the two reactants points to the formation of an EDA complex which could be vital to the reaction outcome. It has previously been shown that excitation of *N*-H–maleimide in the presence of *N*-phenyl glycine can lead to formation of an annulation product.^63^ The proposed mechanism involves the excitation of maleimide and a SET from ground-state *N*-phenyl glycine to the excited-state maleimide. However, UVA light was used, and the reactivity was limited to unsubstituted maleimides. The fact that our system works smoothly with a wide range of N-substituted maleimides, is activated under visible light irradiation, and is run under an open atmosphere suggests that another reaction mechanism is operating. When varying the irradiation wavelength, the reaction outcome is significantly changed. As a reflection of the low absorbance of the reaction mixture above 500 nm, using a 525 nm LED as the irradiation source lowered the reaction rate, and only after 18 h of irradiation the product yield could reach 90% (Table 1, entry 8). Furthermore, UVA light (370 nm) was shown to be ineffective in driving the reaction, giving a yield of 34% after 3 h, albeit the significant absorbance of the reaction mixture in this region (Table 1, entry 12). These results are compatible with the notion that an EDA complex between the glycines and the maleimides acts as an important photoactive intermediate. To establish the formation of such a complex in solution, titration experiments were carried out (Supporting Information). The association constant *K*_EDA_ for the complexes between *N*-(4′-OMe–phenyl) glycine **35** and either phenyl maleimide **2** or methyl maleimide **36** was determined (Scheme 6).

The association constants for the donor–acceptor pairs were observed to be 0.05 M^–1^ for phenyl maleimide and 0.2 M^–1^ for methyl maleimide (see the Supporting Information). These values are comparable to those of previous studies involving EDA complexes dimethyl aniline and maleimides.^20^ The higher association constant for the amine–methyl maleimide complex compared to that for the phenyl maleimide could be attributed to the higher steric demand of the phenyl group.

**Figure 2:**
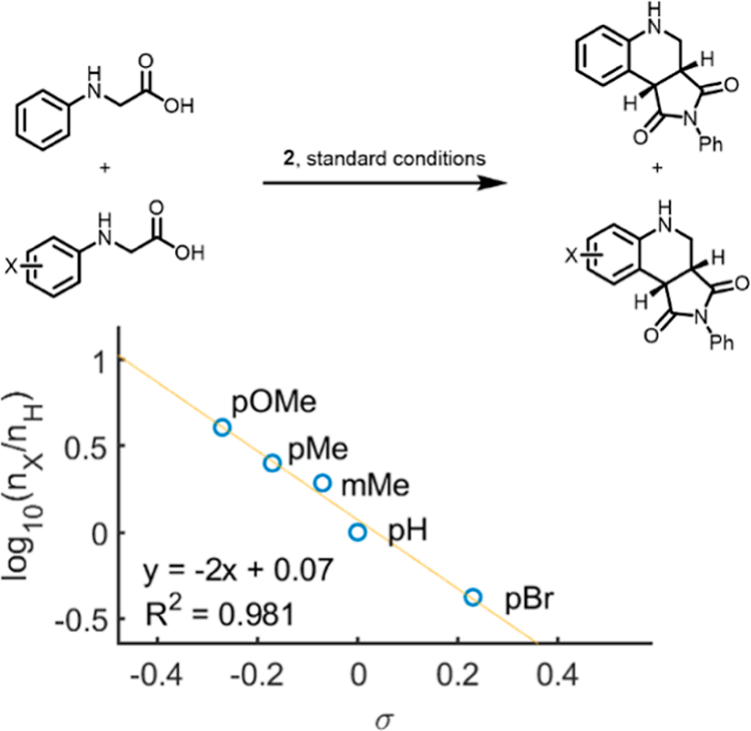
Influence of the electronic properties of the amino acid on the relative reaction rate.

To investigate the influence of the electronic properties of the amine, a series of competition experiments were carried out (Figure 2).

A significant effect of the electronic properties on the relative rate of product formation was observed. Amines with electron-donating groups installed on the aromatic ring reacted faster, whereas electron-withdrawing groups resulted in a decrease in the reaction rate. The large electronic effect is consistent with a build-up of positive charge on the amine, likely due to a single electron oxidation.

The quantum yield of the reaction at 456 nm irradiation was determined to be 5% (Supporting Information), suggesting that the reaction proceeds without any significant contribution from radical chain propagations.

**Scheme 7:**
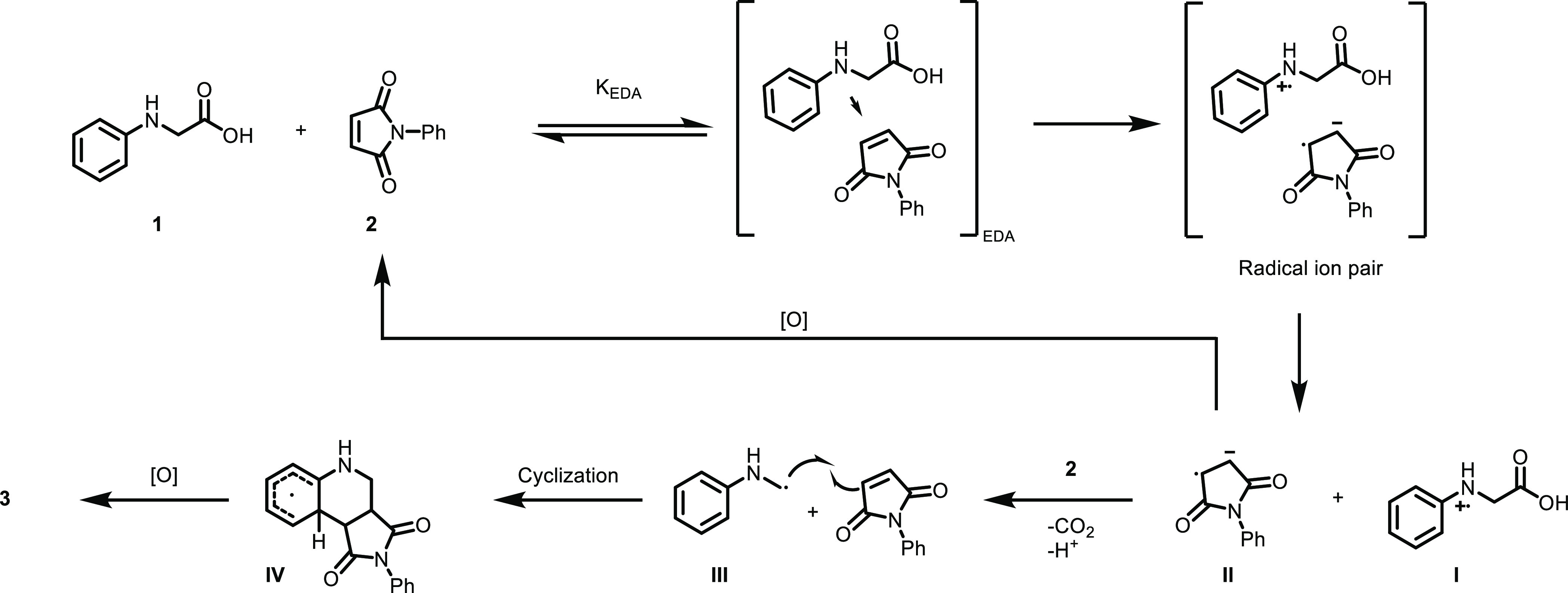
Plausible Reaction Mechanism

With the background of these observations, a plausible reaction mechanism can be postulated (Scheme 7). Initially, the amine **1** and maleimide **2** associate in solution to form an EDA complex which after photoexcitation leads to a radical ion pair. Deprotonation and decarboxylation of radical anion **I** generate the α-aminoalkyl radical (**III**).^23,61^ The radical anion **II** is oxidized by molecular oxygen to reform the maleimide. In the final step, radical **III** reacts with maleimide **2** to form the intermediate **IV**, which after oxidation yields the final product **3**. Since **2** absorbs light above 400 nm (Figure 1), an alternative route that could be operating to some degree is the direct excitation of **2** followed by a SET from ground-state **1** to excited-state **2** resulting in species **I** and **II**.^63^

In summary, a simple, additive-free, and visible light-driven protocol for the synthesis of *N*-H–THQs using simple amino acids and maleimides as starting materials is provided. The reaction is thought to proceed via the photoactivation of a novel EDA complex, with aerobic oxygen as the terminal oxidant. The reaction tolerates a range of aromatic and α-substituents on the amine reaction partner and a range of N-substituted maleimides to provide the desired THQ in moderate to high yields.

## Experimental Section

### General Information

All reagents and solvents were purchased from Sigma-Aldrich and Alfa Aesar and used without any further purification unless specified. Synthesis of maleimides has been described elsewhere.^19^ Purifications were performed using an automated column chromatography Biotage Isolera Spektra One with a Biotage SNAP-10 g KP-silica column together with a 1 g sample cartridge using *n*-heptane or petroleum ether (40–60 °C)/ethyl acetate as the solvent mixture unless otherwise noted. ^1^H (400 MHz) and ^13^C (101 MHz) NMR spectra were acquired on an Agilent NMR machine at 25 °C. The chemical shifts for ^1^H and ^13^C NMR spectra are reported in parts per million (ppm) relative to the residual peak from solvent CDCl_3_ as the internal standard; ^1^H NMR at δ 7.26 ppm and ^13^C NMR at δ 77.16 ppm for CDCl_3_. All coupling constants (*J*) are reported in Hertz (Hz), and multiplicities are indicated by s (singlet), d (doublet), dd (doublet of doublet), td (triplet of doublet), ddd (doublet of doublets of doublets), triplet (t), dt (doublet of triplet), and m (multiplet). Structural assignments were made with additional information from gradient correlation spectroscopy, g heteronuclear single quantum coherence, and g heteronuclear multiple bond correlation experiments. Infrared (IR) spectra were recorded on a Bruker ATR FT-IR spectrometer and are reported in wavenumber (cm^–1^). High-resolution mass spectrometry (HRMS) measurements were performed by CMSI service at Chalmers University of Technology. An Agilent QTOF 6520 equipped with an electrospray interface operated in the positive ionization mode. UV–vis absorption spectra were recorded on a Cary 4000 UV/vis spectrometer, using 1 × 1 cm or 1 × 0.2 cm quartz cuvettes. Light-promoted reactions were carried out in Biotage microwave vials (2–5 mL) under irradiation with a commercial compact fluorescent lamp (Narva Scandinavia, UV light bulb, 15 W, λ_max_ 370 nm), 40 W Kessil PR160L-440 LED lamp (λ_max_ 440 nm), or 40 W Kessil PR160L-525 LED lamp (λ_max_ 525 nm). Gas chromatographic studies were performed using an Agilent 7820A equipped with a flame ionization detector and an Agilent HP-5 19091J-413 column. The emission spectrum of light sources was measured using an AvaSpec-2048-2.

### General Procedure A for the Synthesis of *N*-Aryl Amino Acids

The synthesis was carried out according to a modified published procedure.^64^ Ethyl bromoacetate (11 mmol, 1 equiv), appropriate aniline (18 mmol, 1.7 equiv), and potassium carbonate (22 mmol, 2 equiv) were stirred in acetonitrile (15 mL) at 60 °C for 18 h. After cooling the mixture to room temperature, the mixture was filtered, and the filtrate was concentrated in vacuo. The residue was partitioned between ethyl acetate and water, and the aqueous phase was extracted three times with ethyl acetate. The combined organic layers were dried over sodium sulphate and were concentrated under reduced pressure. The crude product was dissolved in methanol (10 mL), and an aqueous solution of sodium hydroxide (500 mg) was added under strong stirring. The mixture was stirred at room temperature until full conversion as determined by thin layer chromatography (TLC), and the methanol was then removed under reduced pressure. The aqueous phase was washed with ethyl acetate three times and was then acidified to pH 2 with concentrated hydrochloric acid. Ethyl acetate was then used to extract the product three times, and the combined organic layers were dried over sodium sulphate and concentrated in vacuo. The crude product was recrystallized from hot ethanol twice to give the desired *N*-aryl glycine.

### General Procedure B for the Synthesis of *N*-Aryl Amino Acids

Following a published procedure,^65^ the appropriate amino acid (3.2 mmol, 1 equiv), copper iodide (61 mg, 0.32 mmol, 0.1 equiv), potassium carbonate (660 mg, 4.8 mmol, 1.5 equiv), and bromobenzene (500 mg, 3.2 mmol, 1 equiv) were added to a 10–20 mL Biotage microwave vial followed by dimethylacetamide (4 mL). The vial was capped and was purged with nitrogen flow for 10 min. The mixture was then heated to 90 °C for 18 h. Water was added followed by concentrated hydrochloric acid until pH 2. The product was extracted with ethyl acetate three times, and the combined organic layers were dried over sodium acetate, and the solvent was removed under reduced pressure. The solid residue was recrystallized from boiling ethanol twice to yield the desired *N*-aryl amino acid.

#### *p*-Toulylglycine (**S1**)

General procedure A was used to furnish compound **S1** as a slightly yellow solid (1.2 g, 68%). Spectroscopic data are in accordance with the literature;^66^^1^H NMR (400 MHz, DMSO-*d*_6_): δ; ^1^H NMR (400 MHz, DMSO): δ 6.89 (d, *J* = 7.9 Hz, 2H), 6.53–6.46 (m, 2H), 3.75 (d, *J* = 1.1 Hz, 2H), 2.14 (s, 3H) ppm; ^13^C{^1^H} (201 MHz, DMSO-*d*_6_): δ 172.7, 145.6, 129.3, 124.9, 112.5, 45.1, 20.1 ppm.

#### (2,4-Dimethylphenyl)glycine (**S2**)

General procedure A was used to furnish compound **S2** as a slightly white solid (820 mg, 43%). Spectroscopic data are in accordance with the literature;^67^^1^H NMR (400 MHz, DMSO-*d*_6_): δ 6.82–6.76 (m, 2H), 6.29–6.23 (m, 1H), 3.78 (s, 2H), 2.13 (s, 3H), 2.06 (s, 3H); ^13^C{^1^H} (201 MHz, DMSO-*d*_6_): δ 172.9, 143.6, 130.6, 126.9, 124.5, 121.7, 109.3, 45.1, 20.0, 17.4 ppm.

#### (4-Methylphenyl)glycine (**S3**)

General procedure A was used to furnish compound **S3** as a slightly yellow solid (700 mg, 39%). Spectroscopic data are in accordance with the literature;^68^^1^H NMR (400 MHz, DMSO-*d*_6_): δ 6.95 (t, *J* = 7.6 Hz, 1H), 6.42–6.31 (m, 3H), 3.76 (d, *J* = 0.7 Hz, 2H), 2.17 (s, 3H); ^13^C{^1^H} (201 MHz, DMSO-*d*_6_): δ 172.7, 148.1, 137.8, 128.7, 117.3, 112.9, 109.6, 44.8, 21.4 ppm.

#### *N*-Phenylvaline (**S4**)

General procedure B was used to furnish compound **S4** as a slightly yellow solid (320 mg, 52%). Spectroscopic data are in accordance with the literature;^65^^1^H NMR (400 MHz, DMSO-*d*_6_): δ 7.09–6.99 (m, 2H), 6.61 (d, *J* = 8.0 Hz, 2H), 6.56–6.50 (m, 1H), 3.61 (d, *J* = 6.8 Hz, 1H), 2.04 (h, *J* = 6.8 Hz, 1H), 1.00 (d, *J* = 6.8 Hz, 3H), 0.97 (d, *J* = 6.7 Hz, 2H) ppm; ^13^C{^1^H} (201 MHz, DMSO-*d*_6_): δ 174.8, 148.3, 128.8, 116.2, 112.5, 61.9, 30.4, 19.2, 19.0 ppm.

#### *N*-Phenylleucine (**S5**)

General procedure B was used to furnish compound **S5** as a slightly yellow solid (195 mg, 30%). Spectroscopic data are in accordance with the literature;^65^^1^H NMR (800 MHz, DMSO-*d*_6_): δ 7.10–7.01 (m, 2H), 6.57–6.49 (m, 3H), 3.83 (dd, *J* = 8.8, 5.7 Hz, 1H), 1.78 (dt, *J* = 13.5, 6.8 Hz, 1H), 1.68–1.49 (m, 2H), 0.94 (d, *J* = 6.6 Hz, 3H), 0.87 (d, *J* = 6.6 Hz, 3H) ppm; ^13^C{^1^H} (201 MHz, DMSO-*d*_6_): δ 175.9, 148.1, 128.9, 116.1, 112.2, 54.1, 41.1, 24.4, 22.8, 21.8 ppm.

### Synthesis of Maleimide **33**(69)

Following a procedure modified from the literature, maleic anhydride (3.0 g, 31 mmol, 1.5 equiv), ethanolamine (1.3 g, 20 mmol, 1 equiv) in acetic acid (38 mL), and toluene (12 mL) were stirred at 120 °C for 18 h. The solvents were then removed under reduced pressure, and the oily residue was taken up in ethyl acetate and was washed in order with water, saturated sodium bicarbonate solution, and brine. The organic layer was then dried over sodium sulphate and was concentrated under reduced pressure to yield a solid that was recrystallized from ethanol to furnish the desired product as a white solid (1.5 g, 40%).

#### 2-(2,5-Dioxo-2,5-dihydro-1*H*-pyrrol-1-yl)ethyl Acetate (**33**)

Compound **33** was afforded as a white solid (1.5 g, 40%). Spectroscopic data are in accordance with the literature;^69^^1^H NMR (400 MHz, chloroform-*d*): δ 6.73 (s, 1H), 3.86–3.70 (m, 2H), 2.02 (s, 1H); ^13^C{^1^H} (101 MHz, chloroform-*d*): δ 171.0, 170.6, 134.4, 61.6, 37.0, 20.9 ppm.

### General Procedure Oxidative Annulation Reaction

To a 2–5 mL Biotage microwave vial were added N-substituted maleimide (0.1 mmol, 1 equiv) and *N*-phenyl glycine (0.4 mmol, 4 equiv). Methanol (2 mL) and water (1 mL) were then added, and the mixture was stirred until it turned homogeneous. The reaction mixture was then stirred for 3 h under an open atmosphere irradiated with a 440 nm Kessil LED, at a distance of 10 cm. A fan was used to ensure a stable temperature <25 °C. When the reaction was complete, as determined by TLC (SiO_2_, 25% ethyl acetate in *n*-heptane), the methanol was removed in vacuo, and the residue was taken up in 10 mL of ethyl acetate. The organic phase was then washed with saturated sodium hydrogen carbonate solution (2 × 5 mL) and was then dried and evaporated in vacuo. If further purification was needed, the product was loaded on a silica column and eluted with a mixture of ethyl acetate in petroleum ethers to afford the desired THQ product.

#### 5-Methyl-2-phenyl-3a,4,5,9b-tetrahydro-1*H*-pyrrolo[3,4-*c*]quinoline-1,3(2*H*)-dione (**3**)^23^

On a 1.15 mmol scale, compound **3** was afforded as a yellow solid (290 mg, 90%). For the gram-scale version of the reaction, **2** (708 mg, 4.1 mmol, 1 equiv) and **1** (1.7 g, 11.6 mmol, 3 equiv) were dissolved in 70 mL of methanol–water 2:1 in a 100 mL round bottom flask. The reaction mixture was then stirred under an ambient atmosphere at room temperature for 18 h under irradiation with two 40 W Kessil blue LEDs at a distance of 10 cm. The methanol was then removed under reduced pressure, and ethyl acetate was used to dissolve the product. The organic phase was washed with sodium bicarbonate solution and was then dried over magnesium sulphate. The solvent was then removed, and the crude product was purified using flash chromatography (SiO_2_, 0–20% ethyl acetate in petroleum ethers), to yield **3** as a yellow solid (680 mg, 60%). Spectroscopic data are in accordance with the literature;^23^^1^H NMR (400 MHz, chloroform-*d*): δ 7.55 (d, *J* = 7.8 Hz, 1H), 7.47–7.39 (m, 2H), 7.39–7.34 (m, 1H), 7.27 (d, *J* = 5.9 Hz, 2H), 7.15–7.09 (m, 1H), 6.87 (td, *J* = 7.5, 1.3 Hz, 1H), 6.63 (dd, *J* = 8.0, 1.3 Hz, 1H), 4.17 (d, *J* = 9.3 Hz, 1H), 3.78 (dd, *J* = 11.3, 3.2 Hz, 1H), 3.55 (ddd, *J* = 9.3, 4.3, 3.1 Hz, 1H), 3.33 (dd, *J* = 11.3, 4.3 Hz, 1H) ppm; ^13^C{^1^H} (101 MHz, chloroform-*d*): δ 177.6, 175.9, 146.1, 132.1, 130.6, 129.1, 128.7, 128.5, 126.5, 120.2, 116.9, 115.8, 43.4, 41.7, 41.6 ppm.

#### (3a*R*,9b*S*)-2-(4-Fluorophenyl)-3a,4,5,9b-tetrahydro-1*H*-pyrrolo[3,4-*c*]quinoline-1,3(2*H*)-dione (**4**)

Compound **4** was afforded as a yellow solid after purification on a silica column using a gradient of 0–10% ethyl acetate in petroleum ethers (16.2 mg, 54%); mp °C; ^1^H NMR (400 MHz, chloroform-*d*): δ 7.53 (dd, *J* = 7.9, 1.3 Hz, 1H), 7.30–7.23 (m, 2H), 7.11 (dddd, *J* = 8.2, 6.7, 3.3, 1.8 Hz, 3H), 6.88 (td, *J* = 7.5, 1.3 Hz, 1H), 6.63 (dd, *J* = 8.0, 1.2 Hz, 1H), 4.16 (d, *J* = 9.3 Hz, 1H), 3.87–3.73 (m, 2H), 3.54 (ddd, *J* = 9.3, 4.2, 3.0 Hz, 1H), 3.31 (dd, *J* = 11.2, 4.3 Hz, 1H) ppm; ^13^C{^1^H} (101 MHz, chloroform-*d*): δ 177.5, 175.8, 162.2 (d, ^1^*J*_C–F_ = 248.3 Hz), 146.1, 130.6, 128.6, 128.4 (d, ^3^*J*_C–F_ = 8.8 Hz), 127.9 (d, ^4^*J*_C–F_ = 3.3 Hz), 120.4, 116.8, 116.3, 116.1, 115.9, 43.4, 41.7, 41.6 ppm; ^19^F{^1^H} NMR (659 MHz, chloroform-*d*): δ −112.37 ppm; FTIR (ATR) ν: 1369, 1708, 1605, 1509, 1389, 1223, 1773, 820, 757 cm^–1^; HRMS (ESI) *m*/*z*: calcd C_17_H_14_FN_2_O_2_ [M + H]^+^, 297.1039; found, 297.1039.

#### (3a*R*,9b*S*)-2-(4-Chlorophenyl)-3a,4,5,9b-tetrahydro-1*H*-pyrrolo[3,4-*c*]quinoline-1,3(2*H*)-dione (**5**)

Compound **5** was afforded as a yellow solid after purification on the silica column using a gradient of 0–10% ethyl acetate in petroleum ethers (31.5 mg, 87%); mp 188.0–191.5 °C; ^1^H NMR (400 MHz, chloroform-*d*): δ 7.53 (dt, *J* = 7.8, 1.1 Hz, 1H), 7.44–7.37 (m, 2H), 7.26–7.19 (m, 2H), 7.12 (td, *J* = 7.7, 1.5 Hz, 1H), 6.88 (td, *J* = 7.5, 1.3 Hz, 1H), 6.63 (dd, *J* = 8.0, 1.2 Hz, 1H), 4.17 (d, *J* = 9.3 Hz, 1H), 3.77 (dd, *J* = 11.3, 3.1 Hz, 1H), 3.54 (ddd, *J* = 9.3, 4.3, 3.1 Hz, 1H), 3.32 (dd, *J* = 11.3, 4.3 Hz, 1H); ^13^C{^1^H} (101 MHz, chloroform-*d*): δ 177.3, 175.6, 146.1, 134.4, 130.6, 130.5, 129.4, 128.7, 127.7, 120.4, 116.7, 115.9, 43.5, 41.7, 41.6 ppm; FTIR (ATR) ν: 3371, 1710, 1603, 1491, 1387, 1360, 1278, 1195, 1176, 1130, 1089, 1016, 878, 815, 762, 725, 649, 598 cm^–1^; HRMS (ESI) *m*/*z*: calcd C_17_H_14_ClN_2_O_2_ [M + H]^+^, 313.0744; found, 313.0745.

#### (3a*R*,9b*S*)-2-(4-Bromophenyl)-3a,4,5,9b-tetrahydro-1*H*-pyrrolo[3,4-*c*]quinoline-1,3(2*H*)-dione (**6**)

Compound **6** was afforded as a yellow solid after purification on the silica column using a gradient of 0–10% ethyl acetate in petroleum ethers (30 mg, 81%); mp 182.5–184.0 °C; ^1^H NMR (400 MHz, chloroform-*d*): δ 7.61–7.49 (m, 3H), 7.22–7.16 (m, 2H), 7.16–7.08 (m, 1H), 6.88 (td, *J* = 7.5, 1.2 Hz, 1H), 6.63 (dd, *J* = 8.0, 1.2 Hz, 1H), 4.17 (d, *J* = 9.4 Hz, 1H), 3.79 (d, *J* = 3.0 Hz, 1H), 3.76 (t, *J* = 3.0 Hz, 1H), 3.62–3.51 (m, 1H), 3.32 (dd, *J* = 10.8, 4.3 Hz, 1H); ^13^C{^1^H} (101 MHz, chloroform-*d*): δ 177.3, 175.6, 146.1, 132.3, 131.0, 130.6, 128.7, 128.0, 122.5, 120.4, 116.7, 115.9, 43.5, 41.7, 41.6 ppm; FTIR (ATR) ν: 1708, 1601, 1487, 1392, 1353, 1308, 1264, 1196, 1178, 1112, 1069, 1013, 809, 753, 722, 638, 600, 559 cm^–1^; HRMS (ESI) *m*/*z*: calcd C_17_H_14_BrN_2_O_2_ [M + H]^+^, 357.0239; found, 357.0237.

#### (3a*R*,9b*S*)-2-(4-Methoxyphenyl)-3a,4,5,9b-tetrahydro-1*H*-pyrrolo[3,4-*c*]quinoline-1,3(2*H*)-dione (**7**)

Compound **7** was afforded as a white solid after purification on the silica column using a gradient of 0–10% ethyl acetate in petroleum ethers (20 mg, 58%); mp 184.5–186.0 °C; ^1^H NMR (400 MHz, chloroform-*d*): δ 7.54 (d, *J* = 7.6 Hz, 1H), 7.20–7.15 (m, 2H), 7.11 (dddd, *J* = 7.9, 7.3, 1.5, 0.6 Hz, 1H), 6.96–6.91 (m, 2H), 6.87 (td, *J* = 7.5, 1.2 Hz, 1H), 6.63 (dd, *J* = 8.0, 1.2 Hz, 1H), 4.15 (d, *J* = 9.3 Hz, 1H), 3.86–3.73 (m, 5H), 3.56–3.44 (m, 1H), 3.38–3.26 (m, 1H); ^13^C{^1^H} (176 MHz, chloroform-*d*): δ 177.8, 176.1, 159.6, 146.1, 130.7, 128.5, 127.8, 124.8, 120.3, 117.0, 115.9, 114.5, 55.6, 43.3, 41.8, 41.6; FTIR (ATR) ν: 3367, 1707, 1606, 1511, 1392, 1358, 1301, 1250, 1165, 1109, 1029, 819, 757, 582 cm^–1^; HRMS (ESI) *m*/*z*: calcd C_18_H_17_N_2_O_3_ [M + H]^+^, 309.1239; found, 309.1254.

#### (3a*R*,9b*S*)-2-[2-(*tert*-Butyl)phenyl]-3a,4,5,9b-tetrahydro-1*H*-pyrrolo[3,4-*c*]quinoline-1,3(2*H*)-dione (**8**)

Compound **8** was afforded as a white solid after purification on the silica column using a gradient of 0–10% ethyl acetate in petroleum ethers (18.1 mg, 50%); mp 125.0–126.5 °C; ^1^H NMR (400 MHz, chloroform-*d*): δ 7.56 (dd, *J* = 8.1, 1.4 Hz, 1H), 7.52 (dt, *J* = 7.8, 1.1 Hz, 1H), 7.35 (ddd, *J* = 8.1, 7.3, 1.5 Hz, 1H), 7.22–7.16 (m, 1H), 7.13 (dddd, *J* = 7.9, 7.4, 1.5, 0.5 Hz, 1H), 6.87 (td, *J* = 7.5, 1.2 Hz, 1H), 6.68 (dd, *J* = 7.8, 1.5 Hz, 1H), 6.65 (dd, *J* = 8.0, 1.2 Hz, 1H), 4.16 (d, *J* = 9.4 Hz, 1H), 3.83 (s, 1H), 3.76 (dt, *J* = 11.4, 2.6 Hz, 1H), 3.53 (ddd, *J* = 9.4, 4.4, 3.1 Hz, 1H), 3.30 (dd, *J* = 11.2, 4.4 Hz, 1H), 1.35 (s, 9H) ppm; ^13^C{^1^H} (101 MHz, chloroform-*d*): δ 178.9, 176.9, 147.9, 146.3, 131.0, 130.9, 130.6, 129.8, 128.8, 128.5, 127.5, 120.3, 117.1, 115.8, 43.7, 42.0 (2C), 35.8, 31.8 ppm; FTIR (ATR) ν: 3364, 1699, 1489, 1446, 1378, 1318, 1261, 1160, 1122, 882, 810, 750, 728, 633, 582, 555, 541, 529, 501 cm^–1^; HRMS (ESI) *m*/*z*: calcd C_21_H_23_N_2_O_2_ [M + H]^+^, 335.1760; found, 335.1761.

#### (3a*R*,9b*S*)-3a,4,5,9b-Tetrahydro-1*H*-pyrrolo[3,4-*c*]quinoline-1,3(2*H*)-dione (**9**)

Compound **9** was afforded as a white solid after purification on the silica column using a gradient of 0–50% ethyl acetate in petroleum ethers (26.3 mg, 75%). Spectroscopic data are in accordance with the literature;^63^^1^H NMR (400 MHz, acetone-*d*_6_): δ 10.00 (s, 1H), 7.37 (dt, *J* = 7.5, 1.2 Hz, 1H), 7.02 (dddd, *J* = 7.9, 7.2, 1.5, 0.6 Hz, 1H), 6.77–6.65 (m, 2H), 4.04 (d, *J* = 9.2 Hz, 1H), 3.54 (dd, *J* = 11.3, 3.1 Hz, 1H), 3.49 (ddd, *J* = 9.3, 4.4, 3.0 Hz, 1H), 3.14 (ddd, *J* = 11.2, 4.4, 0.8 Hz, 1H) ppm; ^13^C{^1^H} NMR (101 MHz, acetone-*d*_6_): δ 179.8, 178.1, 148.0, 131.1, 128.5, 119.5, 118.5, 116.1, 45.1, 43.4, 41.8 ppm.

#### (3a*R*,9b*S*)-2-Methyl-3a,4,5,9b-tetrahydro-1*H*-pyrrolo[3,4-*c*]quinoline-1,3(2*H*)-dione (**10**)

Compound **10** was afforded as a white solid after purification on the silica column using a gradient of 0–10% ethyl acetate in petroleum ethers (25.6 mg, 60%). Spectroscopic data are in accordance with the literature;^70^^1^H NMR (400 MHz, chloroform-*d*): δ 7.49 (dt, *J* = 7.7, 1.0 Hz, 1H), 7.08 (td, *J* = 7.7, 1.5 Hz, 1H), 6.85 (td, *J* = 7.5, 1.2 Hz, 1H), 6.58 (dd, *J* = 8.0, 1.2 Hz, 1H), 4.00 (d, *J* = 9.1 Hz, 1H), 3.69 (dd, *J* = 11.3, 2.9 Hz, 1H), 3.36 (ddd, *J* = 9.2, 4.3, 2.9 Hz, 1H), 3.23 (dd, *J* = 11.3, 4.3 Hz, 1H), 2.98 (s, 3H) ppm; ^13^C{^1^H} NMR (101 MHz, chloroform-*d*): δ 178.7, 176.9, 146.1, 130.5, 128.4, 120.2, 117.1, 115.8, 43.4, 41.54, 41.48, 25.4 ppm.

#### (3a*R*,9b*S*)-2-Ethyl-3a,4,5,9b-tetrahydro-1*H*-pyrrolo[3,4-*c*]quinoline-1,3(2*H*)-dione (**11**)

Compound **11** was afforded as a white solid after purification on the silica column using a gradient of 0–10% ethyl acetate in petroleum ethers (17.4 mg, 67%). Spectroscopic data are in accordance with the literature;^23^^1^H NMR (400 MHz, chloroform-*d*): δ = 7.50 (dt, *J* = 7.7, 1.1 Hz, 1H), 7.14–7.07 (m, 1H), 6.86 (td, *J* = 7.5, 1.2 Hz, 1H), 6.59 (dd, *J* = 8.0, 1.2 Hz, 1H), 3.98 (d, *J* = 9.2 Hz, 1H), 3.73 (d, *J* = 3.0 Hz, 1H), 3.66 (dt, *J* = 11.2, 3.0 Hz, 1H), 3.55 (qd, *J* = 7.2, 5.8 Hz, 2H), 3.35 (dddd, *J* = 8.9, 4.2, 3.1, 1.0 Hz, 1H), 3.29–3.21 (m, 1H), 1.14 (t, *J* = 7.2 Hz, 3H) ppm; ^13^C{^1^H} NMR (101 MHz, chloroform-*d*): δ 178.4, 176.6, 146.1, 130.5, 128.3, 120.2, 117.2, 115.8, 43.3, 41.7, 41.4, 34.3, 13.1 ppm.

#### (3a*R*,9b*S*)-2-Propyl-3a,4,5,9b-tetrahydro-1*H*-pyrrolo[3,4-*c*]quinoline-1,3(2*H*)-dione (**12**)

Performed on a 0.19 mmol scale. Compound **12** was afforded as a white solid after purification on the silica column using a gradient of 0–10% ethyl acetate in petroleum ethers (33.9, 74%); mp 105.5–107.0 °C; ^1^H NMR (400 MHz, chloroform-*d*): δ 7.53–7.47 (m, 1H), 7.13–7.05 (m, 1H), 6.86 (td, *J* = 7.5, 1.2 Hz, 1H), 6.60 (d, *J* = 8.0 Hz, 1H), 3.98 (d, *J* = 9.1 Hz, 1H), 3.66 (dd, *J* = 11.3, 3.2 Hz, 1H), 3.53–3.39 (m, 2H), 3.38–3.32 (m, 1H), 3.24 (dd, *J* = 11.3, 4.4 Hz, 1H), 1.56 (h, *J* = 7.3 Hz, 2H), 0.81 (t, *J* = 7.4 Hz, 3H) ppm; ^13^C{^1^H} NMR (101 MHz, chloroform-*d*): δ 178.6, 176.9, 145.9, 130.5, 128.4, 120.3, 117.4, 115.8, 43.2, 41.7, 41.4, 40.9, 21.0, 11.2 ppm; FTIR (ATR) ν: 3332, 1771, 1686, 1604, 1489, 1434, 1398, 1339, 1310, 1257, 1204, 1138, 1105, 813, 759, 731, 687, 606, 508 cm^–1^; HRMS (ESI) *m*/*z*: calcd C_14_H_17_N_2_O_2_ [M + H]^+^, 245.1290; found, 245.1293.

#### (3a*R*,9b*S*)-2-Cyclohexyl-3a,4,5,9b-tetrahydro-1*H*-pyrrolo[3,4-*c*]quinoline-1,3(2*H*)-dione (**13**)

Compound **13** was afforded as a white solid after purification on the silica column using a gradient of 0–10% ethyl acetate in petroleum ethers (27.5 mg, 90%); mp 150.5–152 °C; ^1^H NMR (400 MHz, chloroform-*d*): δ 7.48 (dt, *J* = 7.7, 1.1 Hz, 1H), 7.08 (td, *J* = 7.7, 1.5 Hz, 1H), 6.85 (td, *J* = 7.5, 1.2 Hz, 1H), 6.58 (dd, *J* = 8.0, 1.2 Hz, 1H), 4.03–3.86 (m, 2H), 3.72 (s, 1H), 3.61 (dd, *J* = 11.0, 3.1 Hz, 1H), 3.34–3.11 (m, 2H), 2.23–1.98 (m, 2H), 1.87–1.72 (m, 2H), 1.67–1.45 (m, 3H), 1.35–1.12 (m, 3H) ppm; ^13^C{^1^H} NMR (101 MHz, chloroform-*d*): δ 178.6, 176.9, 146.0, 130.6, 128.9, 120.1, 117.4, 115.7, 52.3, 42.9, 41.9, 41.2, 29.0, 28.9, 25.94, 25.89, 25.2 ppm; FTIR ν: 1734, 1700, 1653, 1635, 1559, 1540, 1521, 1506, 1473, 1457, 1374, 668 cm^–1^; HRMS (ESI) *m*/*z*: calcd C_17_H_21_N_2_O_2_ [M + H]^+^, 285.1603; found, 285.1606.

#### (3a*R*,9b*S*)-2-*tert*-Butyl-3a,4,5,9b-tetrahydro-1*H*-pyrrolo[3,4-*c*]quinoline-1,3(2*H*)-dione (**14**)

Compound **14** was afforded as a white solid after simple extraction of excess amino acids and drying (26.2 mg, 95%); mp 163.0–164.0 °C; ^1^H NMR (400 MHz, chloroform-*d*): δ 7.46 (dt, *J* = 7.6, 1.1 Hz, 1H), 7.12–7.05 (m, 1H), 6.84 (td, *J* = 7.5, 1.2 Hz, 1H), 6.59 (dd, *J* = 7.9, 1.2 Hz, 1H), 3.83 (d, *J* = 8.8 Hz, 1H), 3.63–3.56 (m, 1H), 3.25–3.16 (m, 2H), 1.54 (s, 9H) ppm; ^13^C{^1^H} NMR (101 MHz, chloroform-*d*): δ 179.5, 178.0, 145.9, 130.6, 128.3, 120.0, 117.5, 115.7, 58.9, 42.9, 41.8, 41.6, 28.5 ppm; FTIR (ATR) ν: 1695, 1602, 1488, 1364, 1340, 1265, 1218, 1165, 1124, 1105, 1047, 813, 751, 721, 692, 651, 616, 563, 519, 471 cm^–1^; HRMS (ESI) *m*/*z*: calcd C_15_H_19_N_2_O_2_ [M + H]^+^, 259.1447; found, 259.1449.

#### (3a*R*,9b*S*)-2-Benzyl-3a,4,5,9b-tetrahydro-1*H*-pyrrolo[3,4-*c*]quinoline-1,3(2*H*)-dione (**15**)

Compound **15** was afforded as a white solid after purification on the silica column using a gradient of 0–10% ethyl acetate in petroleum ethers (25.9 mg, 81%). Spectroscopic data are in accordance with the literature;^23^^1^H NMR (400 MHz, chloroform-*d*): δ 7.47 (dt, *J* = 7.6, 1.1 Hz, 1H), 7.32–7.19 (m, 5H), 7.07 (td, *J* = 7.6, 1.5 Hz, 1H), 6.84 (td, *J* = 7.5, 1.2 Hz, 1H), 6.57 (dd, *J* = 8.0, 1.2 Hz, 1H), 4.63 (q, *J* = 14.3 Hz, 2H), 3.97 (d, *J* = 9.1 Hz, 1H), 3.75 (s, 1H), 3.62 (dt, *J* = 11.2, 3.0 Hz, 1H), 3.33 (dt, *J* = 9.6, 3.8 Hz, 1H), 3.21 (dd, *J* = 11.3, 4.4 Hz, 1H) ppm; ^13^C{^1^H} NMR (101 MHz, chloroform-*d*): δ 178.2, 176.5, 146.1, 135.7, 130.5, 128.7, 128.5, 128.4, 127.9, 120.2, 117.0, 115.8, 43.4, 42.9, 41.7, 41.5 ppm.

#### (3a*R*,9b*S*)-8-Methoxy-2-phenyl-3a,4,5,9b-tetrahydro-1*H*-pyrrolo[3,4-*c*]quinoline-1,3(2*H*)-dione (**16**)

Compound **16** was afforded as a white solid after purification on the silica column using a gradient of 0–10% ethyl acetate in petroleum ethers (27.6 mg, 75%); mp 136.0–138.0 °C; ^1^H NMR (400 MHz, chloroform-*d*): δ 7.47–7.40 (m, 2H), 7.39–7.33 (m, 1H), 7.30–7.24 (m, 2H), 7.12 (d, *J* = 2.8 Hz, 1H), 6.73 (dd, *J* = 8.7, 2.8 Hz, 1H), 6.58 (d, *J* = 8.7 Hz, 1H), 4.16–4.07 (m, 1H), 3.78 (s, 3H), 3.74 (dd, *J* = 11.3, 3.2 Hz, 1H), 3.54–3.47 (m, 1H), 3.27 (dd, *J* = 11.4, 4.3 Hz, 1H) ppm; ^13^C{^1^H} NMR (101 MHz, chloroform-*d*): δ 177.6, 175.8, 153.7, 140.0, 132.1, 129.2, 128.7, 126.5, 117.7, 116.8, 115.4, 114.9, 55.9, 43.3, 42.4, 41.9 ppm; FTIR (ATR) ν: 3356, 1711, 1509, 1386, 1250, 1190, 1033, 827, 694 cm^–1^; HRMS (ESI) *m*/*z*: calcd C_18_H_17_N_2_O_3_ [M + H]^+^, 309.1239; found, 309.1251.

#### (3a*R*,9b*S*)-8-Methyl-2-phenyl-3a,4,5,9b-tetrahydro-1*H*-pyrrolo[3,4-*c*]quinoline-1,3(2*H*)-dione (**17**)

Compound **17** was afforded as a white solid after purification on the silica column using a gradient of 0–10% ethyl acetate in petroleum ethers (35 mg, 87%). Spectroscopic data are in accordance with the literature;^23^^1^H NMR (400 MHz, chloroform-*d*): δ 7.47–7.39 (m, 2H), 7.39–7.31 (m, 2H), 7.30–7.22 (m, 2H), 6.93 (dt, *J* = 7.9, 1.5 Hz, 1H), 6.55 (d, *J* = 8.1 Hz, 1H), 4.13 (d, *J* = 9.3 Hz, 1H), 3.76 (dd, *J* = 11.1, 3.1 Hz, 1H), 3.70 (s, 1H), 3.52 (ddd, *J* = 9.0, 4.2, 3.1 Hz, 1H), 3.33–3.25 (m, 1H), 2.28 (s, 3H) ppm; ^13^C{^1^H} NMR (101 MHz, chloroform-*d*): δ 177.7, 176.0, 143.8, 132.1, 130.8, 129.5, 129.2, 129.1, 128.6, 126.5, 116.8, 115.8, 43.4, 42.0, 41.6, 20.7 ppm.

#### (3a*R*,9b*S*)-8-Bromo-2-phenyl-3a,4,5,9b-tetrahydro-1*H*-pyrrolo[3,4-*c*]quinoline-1,3(2*H*)-dione (**18**)

Compound **18** was afforded as a white solid after purification on the silica column using a gradient of 0–10% ethyl acetate in petroleum ethers (41.2 mg, 77%); mp 182.5–183.0 °C; ^1^H NMR (400 MHz, chloroform-*d*): δ 7.70–7.65 (m, 1H), 7.47–7.41 (m, 2H), 7.39–7.33 (m, 1H), 7.29–7.23 (m, 2H), 7.20 (dd, *J* = 8.5, 2.2 Hz, 1H), 6.53 (d, *J* = 8.5 Hz, 1H), 4.11 (d, *J* = 9.3 Hz, 1H), 3.76 (dd, *J* = 11.4, 3.2 Hz, 1H), 3.53 (ddd, *J* = 9.3, 4.4, 3.2 Hz, 1H), 3.31 (dd, *J* = 11.4, 4.4 Hz, 1H) ppm; ^13^C{^1^H} NMR (101 MHz, chloroform-*d*): δ 177.1, 175.2, 145.1, 133.1, 131.9, 131.4, 129.2, 128.8, 126.5, 118.6, 117.4, 111.9, 43.0, 41.5, 41.3 ppm; FTIR (ATR) ν: 3391, 1699, 1496, 1381, 1319, 1287, 1267, 1193, 1162, 1134, 883, 806, 749, 698, 622, 568, 537, 510, 492 cm^–1^; HRMS (ESI) *m*/*z*: calcd C_17_H_14_BrN_2_O_2_ [M + H]^+^, 357.0239; found, 357.0241.

#### (3a*R*,9b*S*)-6,8-Dimethyl-2-phenyl-3a,4,5,9b-tetrahydro-1*H*-pyrrolo[3,4-*c*]quinoline-1,3(2*H*)-dione (**20**)

Compound **20** was afforded as a white solid after purification on the silica column using a gradient of 0–10% ethyl acetate in petroleum ethers (27.6 mg, 59%). mp 198.5–200.0 °C; ^1^H NMR (400 MHz, chloroform-*d*): δ 7.46–7.39 (m, 2H), 7.39–7.32 (m, 1H), 7.30–7.23 (m, 3H), 6.88–6.82 (m, 1H), 4.14 (d, *J* = 9.3 Hz, 1H), 3.81 (d, *J* = 11.3 Hz, 1H), 3.73 (s, 1H), 3.52 (ddd, *J* = 9.3, 4.2, 3.1 Hz, 1H), 3.27 (dd, *J* = 11.3, 4.3 Hz, 1H), 2.26 (s, 3H), 2.11 (s, 3H) ppm; ^13^C{^1^H} NMR (101 MHz, chloroform-*d*): δ 177.8, 176.1, 142.0, 132.1, 130.6, 129.1(2C), 128.7, 128.6, 126.5, 122.7, 116.2, 43.6, 42.0, 41.9, 20.7, 16.9 ppm; FTIR (ATR) ν: 3423, 1768, 1699, 1501, 1389, 1332, 1257, 1194, 1177, 1161, 862, 745, 732, 705, 691, 528 cm^–1^; HRMS (ESI) *m*/*z*: calcd C_19_H_19_N_2_O_2_ [M + H]^+^, 307.1447; found, 307.1447.

#### (3a*R*,9b*S*)-9-Methyl-2-phenyl-3a,4,5,9b-tetrahydro-1*H*-pyrrolo[3,4-*c*]quinoline-1,3(2*H*)-dione (**21**) and (3a*R*,9b*S*)-7-Methyl-2-phenyl-3a,4,5,9b-tetrahydro-1*H*-pyrrolo[3,4-*c*]quinoline-1,3(2*H*)-dione (**21′**)

Compounds **21** and **21′** as an inseparable mixture were obtained as a colorless oil after purification on the silica column using a gradient of 0–10% ethyl acetate in petroleum ethers (30.4 mg, 95%). mp 152.0–156.0 °C; ^1^H NMR (400 MHz, chloroform-*d*): δ 7.47–7.40 (m, 6H), 7.39–7.32 (m, 2H), 7.30–7.24 (m, 5H), 7.01 (t, *J* = 7.7 Hz, 1H), 6.77 (dt, *J* = 7.4, 1.0 Hz, 1H), 6.70 (ddd, *J* = 7.8, 1.7, 0.8 Hz, 1H), 6.55–6.48 (m, 1H), 6.45 (t, *J* = 1.2 Hz, 1H), 4.50 (d, *J* = 9.6 Hz, 1.33H, major), 4.13 (d, *J* = 9.3 Hz, 1H, minor), 3.84–3.67 (m, 5H), 3.54 (ddt, *J* = 12.6, 7.7, 2.6 Hz, 2H), 3.30 (dd, *J* = 10.7, 4.3 Hz, 1H), 3.16 (dd, *J* = 11.1, 4.6 Hz, 1H), 2.59 (s, 4H, major), 2.26 (s, 3H, minor) ppm; ^13^C{^1^H} NMR (101 MHz, chloroform-*d*): δ 178.4, 177.7, 176.1, 175.7, 147.7, 146.0, 139.4, 138.6, 132.2, 132.1, 130.4, 129.2, 129.1, 128.7, 128.6, 128.1, 126.6, 126.5, 123.0, 121.3, 117.9, 116.4, 114.1, 11 4.0, 44.9, 43.8, 43.4, 41.8, 41.4, 39.4, 21.3, 20.3 ppm; FTIR (ATR) ν: 1699, 1592, 1479, 1455, 1381, 1273, 1240, 1178, 1037, 873, 789, 747, 691, 620, 605, 570, 540, 510, 485, 470 cm^–1^; HRMS (ESI) *m*/*z*: calcd C_18_H_17_N_2_O_2_ [M + H]^+^, 293.1290; found, 293.1291.

#### (3a*R*,9b*S*)-4-Methyl-2-phenyl-3a,4,5,9b-tetrahydro-1*H*-pyrrolo[3,4-*c*]quinoline-1,3(2*H*)-dione (**22**)

Compound **22** was afforded as an inseparable mixture of diastereomers after purification on the silica column using a gradient of 0–10% ethyl acetate in petroleum ethers (24.1 mg, 79%). Spectroscopic data are in accordance with the literature;^59^^1^H NMR (400 MHz, chloroform-*d*): δ 7.66–7.59 (m, 1H), 7.56 (dd, *J* = 7.7, 2.1 Hz, 1H), 7.49–7.30 (m, 6H), 7.30–7.19 (m, 5H), 7.12 (td, *J* = 7.6, 1.8 Hz, 2H), 6.90–6.81 (m, 2H), 6.62 (dt, *J* = 7.9, 1.6 Hz, 2H), 4.20–4.15 (m, 1H), 4.13–4.08 (m, 1H), 3.72–3.63 (m, 1H), 3.62–3.53 (m, 1H), 3.50–3.42 (m, 1H), 3.17 (ddd, *J* = 8.7, 6.1, 1.8 Hz, 1H), 1.60 (dd, *J* = 6.7, 1.8 Hz, 4H), 1.43 (dd, *J* = 6.5, 1.8 Hz, 3H) ppm; ^13^C{^1^H} (101 MHz, chloroform-*d*): δ 176.5, 175.86, 175.85, 175.8, 146.2, 143.8, 132.0, 130.4, 129.2, 129.1, 128.7, 128.6, 128.5, 126.6, 126.5, 120.2, 119.7, 117.0, 115.77, 115.75, 115.6, 49.2, 47.8, 47.3, 47.1, 43.0, 40.6, 20.2, 18.2 ppm.

#### (3a*R*,9b*S*)-4-Isobutyl-2-phenyl-3a,4,5,9b-tetrahydro-1*H*-pyrrolo[3,4-*c*]quinoline-1,3(2*H*)-dione (**23**)

Compound **23** as an inseparable mixture of diastereomers was afforded as a colorless oil after purification on the silica column using a gradient of 0–10% ethyl acetate in petroleum ethers (16.8 mg, 42%). ^1^H NMR (400 MHz, chloroform-*d*): δ 7.57–7.49 (m, 2H), 7.46–7.37 (m, 5H), 7.37–7.29 (m, 2H), 7.27–7.18 (m, 6H), 7.14–7.06 (m, 3H), 6.84 (dtd, *J* = 15.2, 7.5, 1.2 Hz, 3H), 6.62 (dd, *J* = 7.9, 1.2 Hz, 2H), 6.56 (dd, *J* = 8.0, 1.2 Hz, 1H), 4.18 (d, *J* = 9.1 Hz, 1H), 4.08 (d, *J* = 9.0 Hz, 1H), 3.90–3.81 (m, 2H), 3.69 (s, 1H), 3.52 (dd, *J* = 9.1, 3.8 Hz, 2H), 3.44 (ddd, *J* = 7.8, 6.3, 3.8 Hz, 2H), 3.26 (dd, *J* = 9.0, 3.5 Hz, 1H), 1.96 (dt, *J* = 12.9, 6.5 Hz, 1H), 1.90–1.73 (m, 3H), 1.73–1.35 (m, 4H), 0.98 (d, *J* = 6.4 Hz, 11H), 0.92 (d, *J* = 6.4 Hz, 3H) ppm; ^13^C{^1^H} NMR (101 MHz, chloroform-*d*): δ 177.2, 176.0 (2C), 175.87, 146.4, 143.2, 132.04, 131.99, 130.4, 130.2, 129.2, 129.1, 128.8, 128.63, 128.61, 128.5, 126.6, 126.5, 120.2, 119.7, 117.3, 116.2, 116.1, 115.8, 51.9, 48.6, 47.3, 46.1, 43.3, 41.5, 40.6, 40.3, 25.2, 24.9, 23.5, 23.0, 22.6, 21.8 ppm; FTIR (ATR) ν: 3365, 2955, 1776, 1704, 1602, 1497, 1378, 1257, 1177, 910, 808, 750, 711, 690, 623, 498 cm^–1^; HRMS (ESI) *m*/*z*: calcd C_21_H_22_N_2_O_2_ [M + H]^+^, 335.1760; found, 335.1760.

#### (3a*R*,4*S*,9b*S*)-4-Isopropyl-2-phenyl-3a,4,5,9b-tetrahydro-1*H*-pyrrolo[3,4-*c*]quinoline-1,3(2*H*)-dione (**24**, Less Polar Isomer)

Compound **24** was afforded as a colorless oil after purification on the silica column using a gradient of 0–5% ethyl acetate in petroleum ethers (10.3 mg, 29%). ^1^H NMR (400 MHz, chloroform-*d*): δ 7.54 (d, *J* = 7.7 Hz, 1H), 7.43–7.36 (m, 2H), 7.36–7.29 (m, 1H), 7.24–7.18 (m, 2H), 7.16–7.07 (m, 1H), 6.86 (td, *J* = 7.4, 1.1 Hz, 1H), 6.65 (d, *J* = 8.0 Hz, 1H), 4.20 (d, *J* = 9.1 Hz, 1H), 3.73 (dd, *J* = 9.2, 3.1 Hz, 1H), 2.87 (dd, *J* = 10.1, 3.1 Hz, 1H), 2.72–2.57 (m, 1H), 1.20 (d, *J* = 6.6 Hz, 3H), 1.12 (d, *J* = 6.6 Hz, 3H) ppm; ^13^C{^1^H} NMR (176 MHz, chloroform-*d*): δ 175.9, 175.6, 146.8, 132.0, 130.2, 129.0, 128.6, 128.5, 126.6, 120.4, 117.2, 115.9, 61.0, 44.8, 44.0, 28.6, 20.8, 19.9 ppm; FTIR (ATR) ν: 2962, 1710, 1604, 1498, 1381, 1182, 1134, 751, 691, 624 cm^–1^; HRMS (ESI) *m*/*z*: calcd C_20_H_21_N_2_O_2_ [M + H]^+^, 321.1603; found, 321.1615.

#### (3a*R*,4*R*,9b*S*)-4-Isopropyl-2-phenyl-3a,4,5,9b-tetrahydro-1*H*-pyrrolo[3,4-*c*]quinoline-1,3(2*H*)-dione (**24′**, More Polar Isomer)

Compound **24′** was afforded as a colorless oil after purification on the silica column using a gradient of 0–5% ethyl acetate in petroleum ethers (4.5 mg, 12%). ^1^H NMR (400 MHz, chloroform-*d*): δ 7.50 (d, *J* = 7.7 Hz, 1H), 7.45–7.39 (m, 2H), 7.38–7.31 (m, 1H), 7.26 (s, 2H), 7.10 (td, *J* = 7.8, 1.5 Hz, 1H), 6.81 (t, *J* = 7.5 Hz, 1H), 6.58 (d, *J* = 8.0 Hz, 1H), 4.09 (d, *J* = 9.1 Hz, 1H), 3.58 (dd, *J* = 9.1, 2.8 Hz, 1H), 3.45 (dd, *J* = 9.1, 2.8 Hz, 1H), 2.02–1.89 (m, 1H), 1.06 (d, *J* = 6.7 Hz, 3H), 0.97 (d, *J* = 6.7 Hz, 3H) ppm; ^13^C{^1^H} NMR (201 MHz, chloroform-*d*): δ 177.7, 176.0, 143.3, 132.1, 130.0, 129.1, 128.8, 128.6, 126.5, 119.7, 116.8, 116.0, 56.8, 45.0, 40.8, 29.2, 20.0, 19.0 ppm; FTIR (ATR) ν: 2961, 1707, 1602, 1497, 1382, 1260, 1178, 911, 810, 735, 712, 691, 624, 483 cm^–1^; HRMS (ESI) *m*/*z*: calcd C_20_H_21_N_2_O_2_ [M + H]^+^, 321.1603; found, 321.1610.

#### (3a*R*,9b*S*)-5-Methyl-2-phenyl-3a,4,5,9b-tetrahydro-1*H*-pyrrolo[3,4-*c*]quinoline-1,3(2*H*)-dione (**25**)

Compound **25** was afforded as a white solid after purification on the silica column using a gradient of 0–10% ethyl acetate in petroleum ethers (28 mg, 76%). Spectroscopic data are in accordance with the literature;^20^^1^H NMR (400 MHz, chloroform-*d*): δ 7.53 (dt, *J* = 7.5, 1.3 Hz, 1H), 7.47–7.39 (m, 2H), 7.39–7.33 (m, 1H), 7.31–7.20 (m, 3H), 6.91 (td, *J* = 7.5, 1.2 Hz, 1H), 6.75 (dd, *J* = 8.2, 1.2 Hz, 1H), 4.16 (d, *J* = 9.6 Hz, 1H), 3.62 (dd, *J* = 11.4, 2.8 Hz, 1H), 3.54 (ddd, *J* = 9.6, 4.4, 2.7 Hz, 1H), 3.13 (dd, *J* = 11.5, 4.4 Hz, 1H), 2.84 (s, 3H) ppm; ^13^C{^1^H} NMR (101 MHz, chloroform-*d*): δ 177.8, 175.9, 148.7, 132.1, 130.5, 129.1, 128.8, 128.6, 126.5, 119.8, 118.7, 112.7, 50.8, 43.7, 42.3, 39.6 ppm.

### Synthesis of *N*-Acylated Product **28**

Following a modified literature procedure,^71^ to a Biotage 2–5 mL microwave vial were added **3** (32.1 mg, 0.12 mmol, 1 equiv) and dichloromethane (DCM) (2 mL) followed by pyridine (12 μL, 0.15 mmol, 1.2 equiv). Acetyl chloride (17.2 mg, 0.21 mmol, 1.9 equiv) in 1 mL of DCM was then added dropwise. The reaction mixture was stirred at 23 °C until full conversion (ca 2 h). The reaction mixture was then washed with aqueous saturated ammonium chloride, and the solvent was removed under reduced pressure. The crude product was purified using flash chromatography (SiO_2_, 0–5% methanol in DCM).

#### (3a*R*,9b*S*)-5-Acetyl-2-phenyl-3a,4,5,9b-tetrahydro-1*H*-pyrrolo[3,4-*c*]quinoline-1,3(2*H*)-dione (**28**)

Compound **28** was afforded as a yellow solid (36.0 mg, 97%). mp 142.5–144 °C; ^1^H NMR (800 MHz, chloroform-*d*): δ 7.61–7.57 (m, 1H), 7.43 (t, *J* = 7.7 Hz, 2H), 7.40–7.34 (m, 2H), 7.34–7.28 (m, 1H), 7.24–7.11 (m, 3H), 5.13 (s, 1H), 4.22 (d, *J* = 9.2 Hz, 1H), 3.62 (s, 1H), 3.33 (s, 1H), 2.10 (s, 3H); ^13^C{^1^H} NMR (101 MHz, chloroform-*d*): δ 176.4, 175.0, 169.5, 140.5, 131.7, 130.8, 129.3, 129.0, 128.8, 128.7, 127.2, 126.4, 124.9, 44.4, 43.6, 29.8, 22.2 ppm. FTIR (ATR) ν: 1708, 1652, 1582, 1492, 1458, 1382, 1307, 1185, 1158, 1120, 1060, 817, 779, 760, 691, 629, 616, 524 cm^–1^; HRMS (ESI) *m*/*z*: calcd C_19_H_17_N_2_O_3_ [M + H]^+^, 321.1239; found, 321.1254.

### Synthesis of *N*-Benzoylated Product **29**

Following a modified literature procedure,^72^ to a Biotage 2–5 mL microwave vial were added **3** (29.1 mg, 0.10 mmol, 1 equiv) and toluene (2 mL) followed by triethyl amine (30 μL, 0.2 mmol, 2.1 equiv) and toluoyl chloride (20 mg, 0.13 mmol, 1.2 equiv). The reaction mixture was stirred at 40 °C for 18 h. The solvent was then removed under reduced pressure, and the crude was purified using flash chromatography (SiO_2_, 0–40% EtOAc in *n*-pentane).

#### (3a*R*,9b*S*)-5-(4-Methylbenzoyl)-2-phenyl-3a,4,5,9b-tetrahydro-1*H*-pyrrolo[3,4-*c*]quinoline-1,3(2*H*)-dione (**29**)

Compound **29** was afforded as a white solid (38.0 mg, 92%). mp 87.0–89.5 °C; ^1^H NMR (400 MHz, chloroform-*d*): δ 7.63–7.56 (m, 1H), 7.44–7.29 (m, 3H), 7.23–7.12 (m, 5H), 7.07–6.99 (m, 3H), 6.64 (d, *J* = 8.1 Hz, 1H), 5.27 (d, *J* = 13.1 Hz, 1H), 4.32 (d, *J* = 9.4 Hz, 1H), 3.72 (ddd, *J* = 9.4, 5.2, 2.1 Hz, 1H), 3.42 (dd, *J* = 13.2, 5.2 Hz, 1H), 2.30 (s, 3H); ^13^C{^1^H} NMR (101 MHz, chloroform-*d*): δ 176.3, 175.2, 169.7, 141.6, 140.8, 131.7, 131.6, 130.7, 129.4, 129.1, 129.0, 128.98, 128.2, 126.4, 126.0, 125.2, 124.5, 44.6, 44.1, 43.5, 21.6 ppm. FTIR (ATR) ν: 1712, 1636, 1375, 1176, 1148, 1077, 901, 831, 814, 753, 744, 729, 610, 526 cm^–1^; HRMS (ESI) *m*/*z*: calcd C_25_H_21_N_2_O_3_ [M + H]^+^, 397.1552; found, 397.1559.

### Reaction of **3** with Mesyl Chloride

Following a published procedure,^73^**3** (29.3 mg, 0.105 mmol, 1 equiv) was dissolved in toluene (1 mL) in a 2–5 mL Biotage microwave vial. Pyridine (15 μL, 0.19 mmol, 1.8 equiv) and mesyl chloride (30 μL, 0.39 mmol, 3.7 equiv) were then added, and the vial was then capped and stirred at 90 °C until full conversion of **3** (as determined by TLC, 5 h reaction time). The solvent was then removed under reduced pressure, and the product was isolated after flash chromatography (SiO_2_, 10–50% EtOAc in petroleum ethers).

#### (3a*R*,9b*S*)-5-(Methylsulfonyl)-2-phenyl-3a,4,5,9b-tetrahydro-1*H*-pyrrolo[3,4-*c*]quinoline-1,3(2*H*)-dione (**30**)

Compound **30** was afforded as a yellow oil (31.0 mg, 83%). ^1^H NMR (400 MHz, chloroform-*d*): δ 7.71 (ddd, *J* = 7.6, 1.8, 0.9 Hz, 1H), 7.58 (dd, *J* = 8.1, 1.3 Hz, 1H), 7.48–7.42 (m, 2H), 7.42–7.32 (m, 2H), 7.31–7.26 (m, 3H), 4.39 (dd, *J* = 13.8, 4.2 Hz, 1H), 4.24 (d, *J* = 9.5 Hz, 1H), 3.79 (dd, *J* = 13.8, 5.6 Hz, 1H), 3.67 (ddd, *J* = 9.7, 5.5, 4.2 Hz, 1H), 3.02 (s, 3H) ppm; ^13^C{^1^H} NMR (101 MHz, chloroform-*d*): δ 175.7, 174.7, 137.5, 131.7, 131.2, 129.4, 129.0, 128.9, 126.40, 126.35, 124.2, 122.9, 45.4, 43.2, 41.9, 40.7 ppm. FTIR (ATR) ν: 1708, 1491, 1457, 1385, 1328, 1150, 1039, 993, 960, 910, 838, 798, 726, 693, 648 cm^–1^; HRMS (ESI) *m*/*z*: calcd C_18_H_17_N_2_O_4_S [M + H]^+^, 357.0909; found, 357.0914.

### Oxidation of **3** to Quinoline **31**

Following a modified published procedure,^59^**3** (32.1 mg, 0.115 mmol, 1 equiv) and 2,3-dichloro-5,6-dicyanobenzoquinone (DDQ) (55.2 mg, 0.24 mmol, 2.1 equiv) were dissolved in toluene (2 mL) in a 2–5 mL Biotage microwave vial and heated at 90 °C for 18 h using a heating block. The solvent was then removed under reduced pressure, and the crude was passed through a short silica plug and eluted with 100% DCM to yield the quinoline **31**.

#### 2-Phenyl-1*H*-pyrrolo[3,4-*c*]quinoline-1,3(2*H*)-dione (**31**)

Compound **31** was afforded as white crystals (29.1 mg, 92%). Spectroscopic data are in accordance with the literature;^74^^1^H NMR (400 MHz, chloroform-*d*): δ 9.45 (s, 1H), 8.89 (ddd, *J* = 8.4, 1.4, 0.7 Hz, 1H), 8.29 (dt, *J* = 8.6, 0.9 Hz, 1H), 7.96 (ddd, *J* = 8.5, 6.9, 1.5 Hz, 1H), 7.82 (ddd, *J* = 8.2, 6.9, 1.2 Hz, 1H), 7.60–7.51 (m, 2H), 7.51–7.42 (m, 3H) ppm; ^13^C{^1^H} NMR (101 MHz, chloroform-*d*): δ 167.3, 166.8, 152.3, 143.9, 135.2, 133.0, 131.3, 130.5, 130.4, 129.4, 128.6, 126.7, 125.2, 123.5, 121.6 ppm.

### Synthesis of Quinoline **34**

Following the general procedure, maleimide **33** (55.4 mg, 0.30 mmol, 1 equiv) and amino acid **32** (168.6 mg, 1.0 mmol, 3.4 equiv) were dissolved in a mixture of 1 mL of water and 2 mL of methanol and irradiated for 18 h. The reaction mixture was then mixed with ethyl acetate (15 mL) and the organic layer washed with saturated sodium bicarbonate solution (2 × 10 mL) to remove the excess amino acid. The organic layer was then dried over sodium sulphate and concentrated under reduced pressure to yield **33** that was used without further purification. In the next step, **33** was dissolved in 1 mL of toluene, and DDQ (90.4 mg, 0.40 mmol) was added, and the mixture was stirred at 90 °C for 18 h. The solvent was then removed under reduced pressure, and the product was isolated using flash chromatography (SiO_2_, 0–10% EtOAc in petroleum ethers).

#### 2-(4-Methyl-1,3-dioxo-1,3-dihydro-2*H*-pyrrolo[3,4-*c*]quinolin-2-yl)ethyl Acetate (**34**)

Compound **34** was afforded as off-white crystals (54.8 mg, 61% over two steps). Spectroscopic data are in accordance with the literature;^75^^1^H NMR (400 MHz, chloroform-*d*): δ 8.79 (ddd, *J* = 8.4, 1.5, 0.7 Hz, 1H), 8.13 (d, *J* = 8.6 Hz, 1H), 7.88 (ddd, *J* = 8.6, 6.9, 1.5 Hz, 1H), 7.71 (ddd, *J* = 8.2, 6.9, 1.2 Hz, 1H), 4.36 (dd, *J* = 5.8, 4.8 Hz, 2H), 4.01 (dd, *J* = 5.8, 4.8 Hz, 2H), 3.05 (s, 3H), 2.03 (s, 3H) ppm; ^13^C{^1^H} NMR (101 MHz, chloroform-*d*): δ 171.1, 168.3, 168.1, 155.1, 151.6, 136.1, 132.9, 129.3, 129.1, 125.0, 122.0, 120.7, 61.6, 37.3, 22.2, 20.9 ppm.

## Data Availability

The data underlying this study are available in the published article and its online Supporting Information material.

## Supporting Information Available

UV–vis studies, emission spectrum of the irradiation source, mechanistic investigations, and NMR spectra (PDF)
