# Supplementary material for: Overcoming Back Electron Transfer in the Electron Donor–Acceptor Complex-Mediated Visible Light-Driven Generation of α-Aminoalkyl Radicals from Secondary Anilines
Source: J Org Chem. 2022 Dec 8;88(1):462–74. doi: 10.1021/acs.joc.2c02448 (PMC9830629; doi:10.1021/acs.joc.2c02448)
Supplement: Supplementary file 1 — jo2c02448_si_001.pdf [file jo2c02448_si_001.pdf]

Supporting Information for

Overcoming Back Electron Transfer in the EDA Complex Mediated Visible Light Driven Generation of α-Aminoalkyl Radicals from Secondary Anilines

August Runemark[a] and Henrik Sundén[a, b*]

[a]Department of Chemistry and Chemical Engineering, Chalmers University of Technology, Kemivägen 10, 41296 Gothenburg, Sweden
[b]Chemistry and Molecular Biology, University of Gothenburg, Kemivägen 10, 412 96, Gothenburg, Sweden.

Contents

1.1 UV-vis spectrum of *N*-Phenyl glycine, *N*-phenyl maleimide and their mixtures S2

1.2 UV-vis spectrum of *N*-Phenyl glycine, *N*-phenyl maleimide and their mixtures in different solvents S2

1.3 Emission spectra of the irradiation sources S3

1.4 Investigation of the EDA complex stoichiometry S3

1.5 Determination of the association constants of the EDA complexes between maleimides and *N*-aryl glycines S3

1.6 Competition experiments S8

1.7 Quantum yield determination S9

1.8 References S12

1.9 NMR spectra S13

1.1 UV-vis spectrum of *N*-Phenyl glycine, *N*-phenyl maleimide and their mixtures

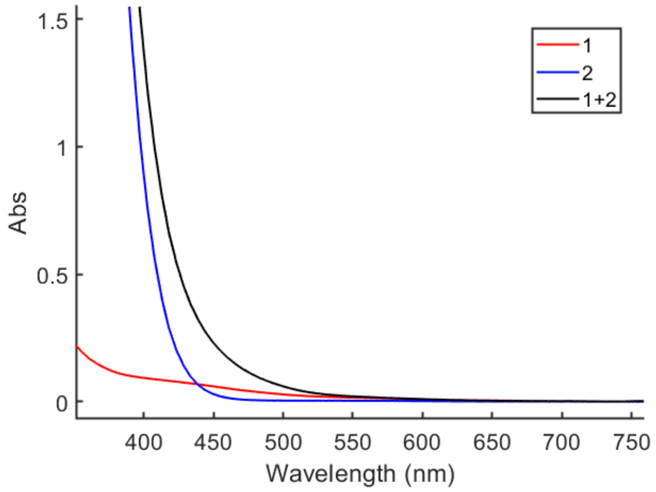

*Figure S1.UV-vis spectrum of **1** (0.09 M), **2** (0.03 M) and their mixture at the same concentrations in methanol-water mixture (2:1). Solutions prepared in 3 mL solvent and measurements carried out in a 1x1 cm quartz cuvette. Absorption corrected for the absorption of the pure solvent.*

1.2 UV-vis spectrum of *N*-Phenyl glycine, *N*-phenyl maleimide and their mixtures in different solvents

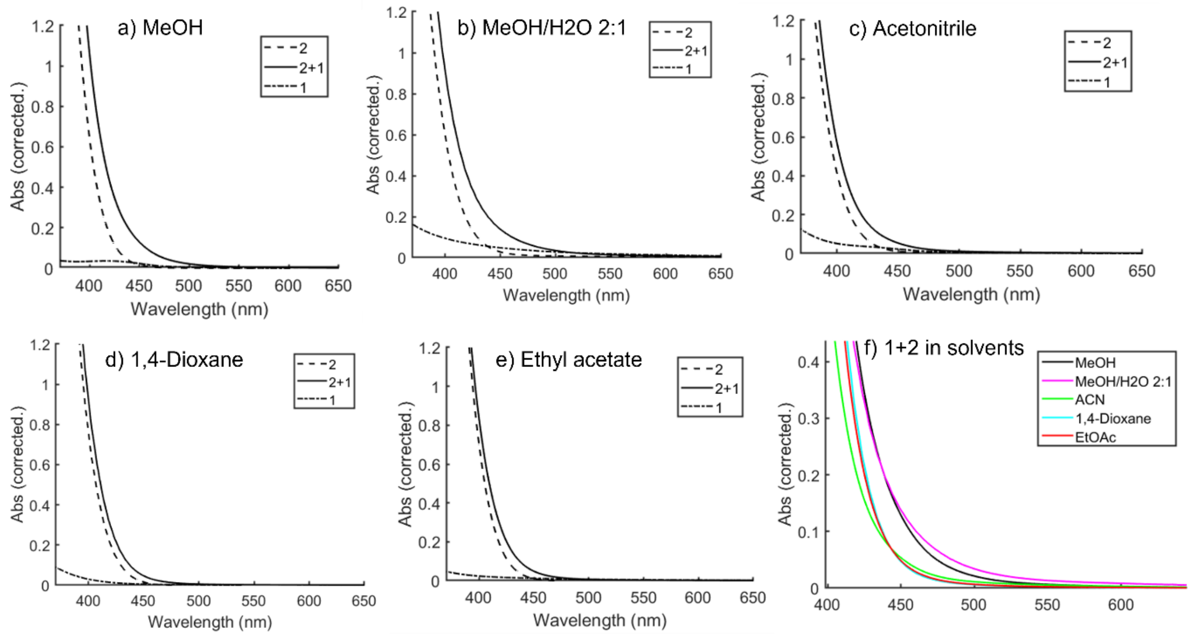

*Figure S2. UV-vis spectrum (370 – 650 nm) of **1** (0.15 M), **2** (0.15 M) and their mixture, at the same concentrations in a) methanol, b) methanol-water mixture 2:1, c) acetonitrile, d) 1,4-dioxane, e) ethyl acetate, and f) overlaying absorption of the mixtures of **1** and **2** in the different solvents. All solutions were prepared in 1 mL solvent and measured in a quartz cuvette with 2 mm path length. All spectra are corrected according to the absorbance of the pure solvent.*

1.3 Emission spectra of the irradiation sources

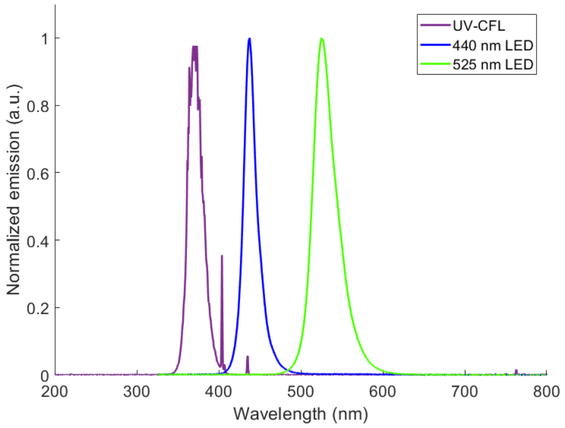

Figure S3. Normalized emission of the UV-CFL (λ_{max} = 370 nm), the 440 nm Kessil LED (λ_{max} = 438 nm) and 525 nm Kessil LED (λ_{max} = 525 nm).

1.4 Investigation of the EDA complex stoichiometry

To elucidate the binding stoichiometry of the amine-maleimide complex, the absorbance of the CT-band was recorded at different molar ratios of amine: maleimide (Figure S4).

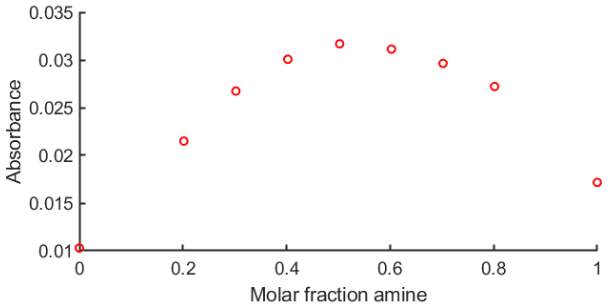

Figure S4. Job's plot of the EDA complex between maleimide and aryl glycine.

The maximal absorbance was observed when the donor and acceptor were mixed in 1:1 molar ratio. This result indicates that the EDA complex forms according to a 1:1 binding model, and for the subsequent investigations this is assumed to be the case.

1.5 Determination of the association constants of the EDA complexes between maleimides and *N*-aryl glycines

The association constants were calculated using the Benesi-Hildebrand method and additionally fitted using a program developed by Thordarson.[1,2]

*Scheme S1. Structures of the amine **35**, maleimide **36** and their EDA complex **37**.*

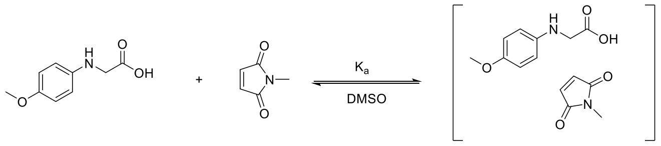

35 **36** **37**

To a solution of amine (**35**) in DMSO (0.0972 M) was added varying amounts of methyl maleimide (**36**) (0.19 – 1.9 M, 2 – 20 equivalents), inducing the formation of the complex **37** (Scheme S1. Structures of the amine **35**, maleimide **36** and their EDA complex **37**.Scheme S1). The absorbance was recorded for each sample (Figure S5a) in a 0.2 cm cuvette. The molar absorptivity of methyl maleimide in DMSO was also determined (Figure S5b).

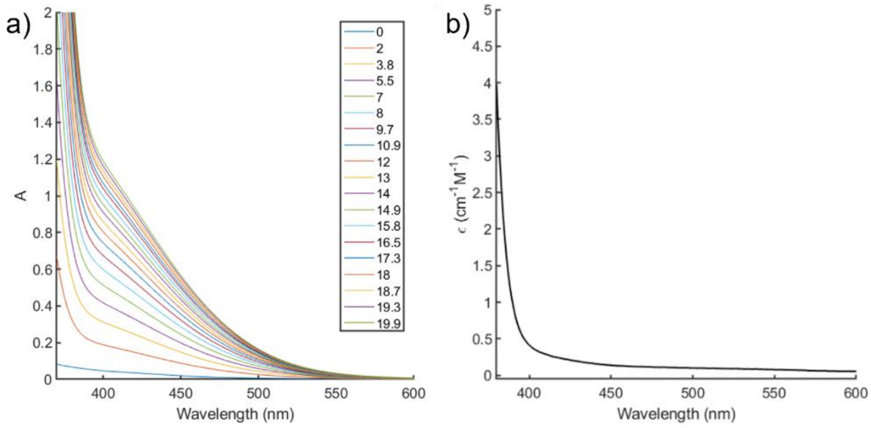

*Figure S5. a) Absorbance of mixture of amine **35** and maleimide **36** with increasing equivalents of maleimide **36** in DMSO; b) molar absorptivity of maleimide **36** in DMSO*

Due to the methyl maleimide having some absorption (ϵ < 0.2 cm^{-1}M^{-1} above 450 nm) overlapping with the formed EDA complex and therefore interferes with the titration, its contribution to the total absorption needs to be considered in the determination of the association constant (eq. 1).

$$A_{tot}(wavelength) = \varepsilon_{35}(wavelength)[\mathbf{35}] + \varepsilon_{36}(wavelength)[\mathbf{36}] + \varepsilon_{37}(wavelength)[\mathbf{37}] \qquad (1)$$

Where $\varepsilon_i(wavelength)$, is the molar absorptivity of compound i, at a certain wavelength of interest. The concentration of **35** is held constant during the titration, and it is assumed that its contribution to the total absorbance of the mixture is constant. It is also assumed that $[\mathbf{35}] \approx [\mathbf{35}]_0$ and $[\mathbf{36}] \approx [\mathbf{36}]_0$ (where index 0 indicates the concentration prior to complexation). With these assumptions we can rearrange eq. 1 to eq. 2, and attribute the change in absorption, ΔA, only to the formation of **37**:

$$\varepsilon_{37}(wavelength)[\mathbf{37}] = \Delta A = A_{tot}(wavelength) - \varepsilon_{35}(wavelength)[\mathbf{35}]_0 - \varepsilon_{36}(wavelength)[\mathbf{36}]_0 \quad (2)$$

Where $\varepsilon_{35}(wavelength)[\mathbf{35}]_0$ is determined simply as the absorbance of the amine **35** prior to addition of maleimide, and $\varepsilon_{36}(wavelength)[\mathbf{36}]_0$ is calculated using the data from Figure S5b. The ΔA at a certain set of wavelength (460, 470, 480 and 500 nm) is then plotted as a function of the ratio

[36]$_0$/**[35]**$_0$ to construct titration curves that can be simultaneously fit to extract the $\varepsilon_{37}(wavelength)$ and K_a (Figure S6).[1]

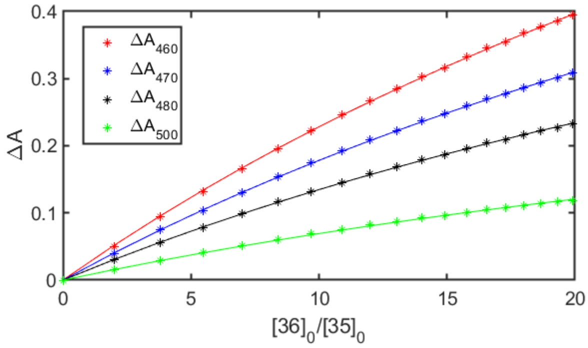

*Figure S6. Titration curves and non-linear fitting for the complex **37** .*

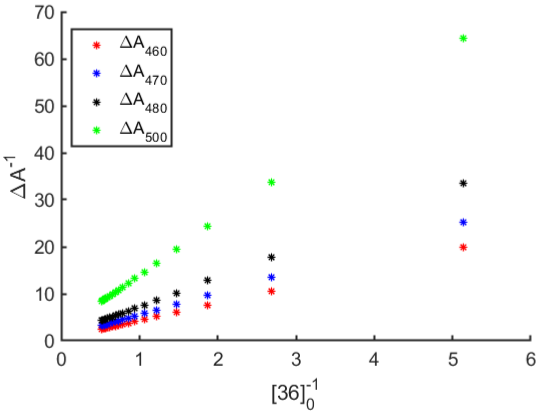

*Figure S7. Benesi-Hildebrand analysis of the titration curve for complex **37**.*

*Table S1. Molar absorptivities of the EDA complex between **35** and **36** at different wavelengths and association constant K_a for the EDA complex between **35** and **36** estimated using non-linear fitting and Benesi-Hildebrand method.*

	Non-linear fitting	Benesi-Hildebrand
K_a (M^{-1})	0.19	0.16
ε_{37}(460) ($M^{-1}cm^{-1}$)	77	90
ε_{37}(470) ($M^{-1}cm^{-1}$)	60	83
ε_{37}(480) ($M^{-1}cm^{-1}$)	46	52
ε_{37}(500) ($M^{-1}cm^{-1}$)	24	26

Following the same methodology as outlined above, the association constant for the complex between aniline **35** and *N*-phenyl maleimide **2** was then determined (Scheme 2).

*Scheme 2. Structures of the amine **35**, maleimide **2** and their EDA complex **38**.*

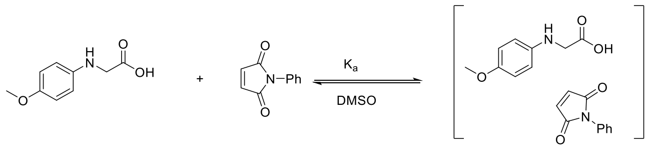

35 **2** **38**

To a solution of amine (**35**) in DMSO (0.0966 M) was added varying amounts of phenyl maleimide (**2**) (0.1 – 2.7 M, 2 – 28 equivalents), inducing the formation of the complex **38**. The absorbance was recorded for each sample (Figure S8a) in a 0.2 cm cuvette. The molar absorptivity of **2** in DMSO was also determined (Figure S8b).

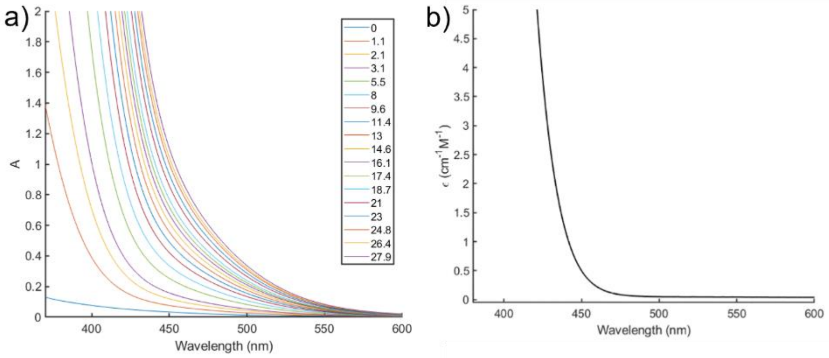

*Figure S8 a) Absorbance of mixture of amine **35** and maleimide **2** with increasing equivalents of maleimide **2** in DMSO; b) molar absorptivity of maleimide **2** in DMSO*

Following the same procedures as for the complex between **35** and **36**, the association constant was determined using global fitting and with Benesi-Hildebrand method (Figure S9 and Figure S10).

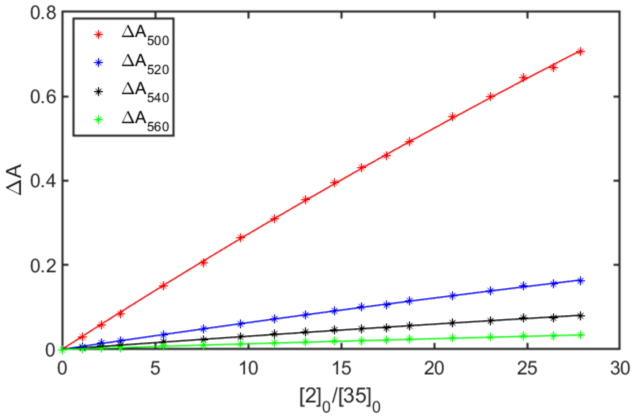

*Figure S9. Titration curves and non-linear fitting for the complex **38**.*

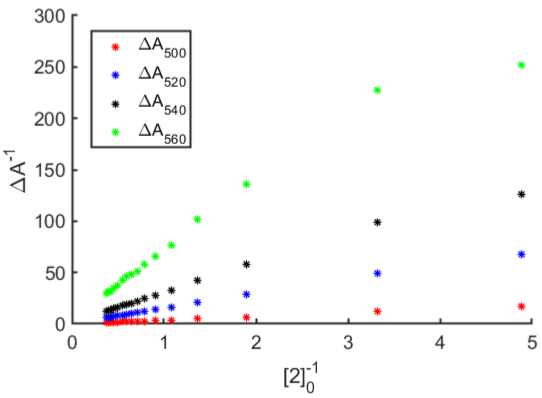

*Figure S10. Benesi-Hildebrand analysis of the titration curve for complex **38**.*

Table S2. Molar absorptivities of the EDA complex between 35 and 2 at different wavelengths and association constant Ka for the EDA complex between 35 and 2 estimated using non-linear fitting and Benesi-Hildebrand method.

	Non-linear fitting	Benesi-Hildebrand
K_a (M^{-1})	0.05	0.06
ε_{38}(500) ($M^{-1}cm^{-1}$)	127	560
ε_{38}(520) ($M^{-1}cm^{-1}$)	71	94
ε_{38}(540) ($M^{-1}cm^{-1}$)	35	32
ε_{38}(560) ($M^{-1}cm^{-1}$)	15	7

1.6 Competition experiments

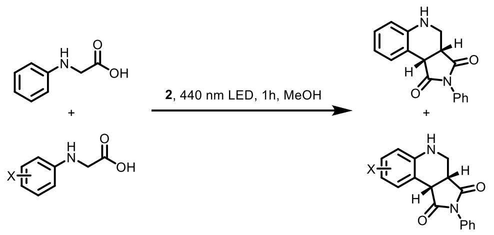

To investigate the impact of substituents on the amine reaction partner, four reactions were set up according to the following procedure: To a 2-5 mL Biotage microwave vial was added **2** (0.05 mmol), **1** (0.2 mmol), substituted *N*-phenyl glycines (4'-OMe, 4'-Me, 4'-Br and 3'-Me respectively) (0.2 mmol) and 2 mL methanol. The mixtures were then irradiated with a 440 nm LED for 1 hour. The crude reaction mixture was then analyzed by ^{1}H NMR. The ratio between the two competitive products from were determined and to establish the electronic effect, the logarithm of the ratios were plotted against the Hammett σ_p parameter (Figure 11).[3] Average ratios from two different runs were used to construct the plot.

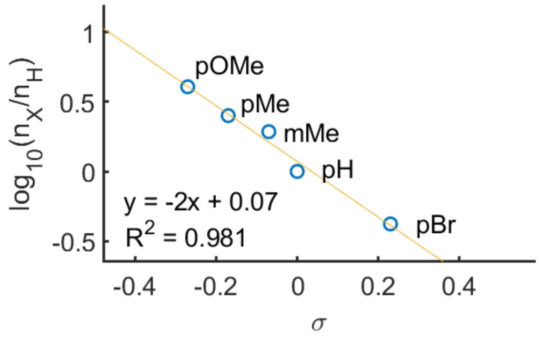

Figure 11. Hammett plot.

1.7 Quantum yield determination

In order to determine the quantum yield of the reaction, i.e., the ratio between the molar amount of product formed and the molar amount of photons absorbed by the reaction mixture, potassium ferrioxalate actinometry was applied.[2]

Three solutions where prepared accordingly:

An actinometer solution was made by dissolving potassium ferrioxalate (590 mg) and 280 µL concentrated sulfuric acid in 100 mL deionized water in a volumetric flask. All preparations, and subsequent storage, of this solution were carried out in the dark.

A buffer solution was separately prepared from sodium acetate (4.9 g) and concentrated sulfuric acid (1 mL) in 100 mL deionized water in a volumetric flask.

Finally, anhydrous 1,10-phenanthroline (199 mg) was dissolved in 100 mL water in a separate volumetric flask.

To determine the photon flux of the irradiation source, actinometer solution (2 mL) in a 1x1 cm quartz cuvette placed in a sample holder was irradiated by a Kessil PR160L-456 (set at 25% output) located 28 cm from the sample holder. In total 6 different samples were irradiated during a set time period of 5s, 10s, 20s ,40s, 60s and 300s. After the set time, the solution was transferred to a 10 mL volumetric flask containing 2 mL buffer solution and 0.5 mL 1,10-phenanthroline solution. Deionized water was used to dilute the solution to 10 mL in total. Protected from light, the prepared solutions were then stored for 30 minutes before their respective UV-vis spectrum was recorded (using a 1x1 cm quartz cuvette). A blank sample, based on the non-radiated actinometer solution, was prepared identically. The difference in absorbance between each of the irradiated sample and the blank was then plotted (Figure S12).

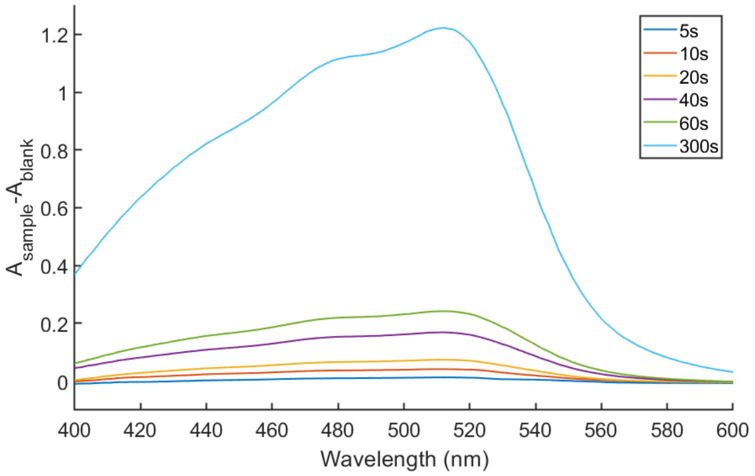

Figure S12. UV-vis spectra of each irradiated sample, compared to the non-irradiated blank.

For each sample, the absorbance at 510 nm versus the blank sample was noted. Using the molar absorptivity at 510 nm of the formed Fe(II)phen$_3$ complex, the molar amount of iron(II) formed during the irradiation was determined according to equation (3).

$$moles\ Fe(II) = \frac{V1*V3*\Delta A_{510\ nm}}{V2*l*\varepsilon_{510\ nm}} \qquad (3)$$

Where V1 is the volume of the irradiated sample, V2 is the volume of the irradiated sample taken for the determination of amount of Fe(II), V3 is the final volume of the sample measured, l is the path

length of light (1 cm), $\Delta A_{510\,nm}$ is the difference in absorption between the irradiated samples and the reference sample kept in dark, and $\varepsilon_{510\,nm}$ is the molar absorptivity of the iron(II) phenanthroline complex (11 100 L mol^{-1} cm^{-1}) at 510 nm. For each sample with increasing irradiation time, the molar amount of iron was determined to be 0.012, 0.037, 0.067, 0.15, 0.22 and 1.1 µmol respectively.

The amount of iron(II) was then plotted as a function of irradiation time (Figure S13).

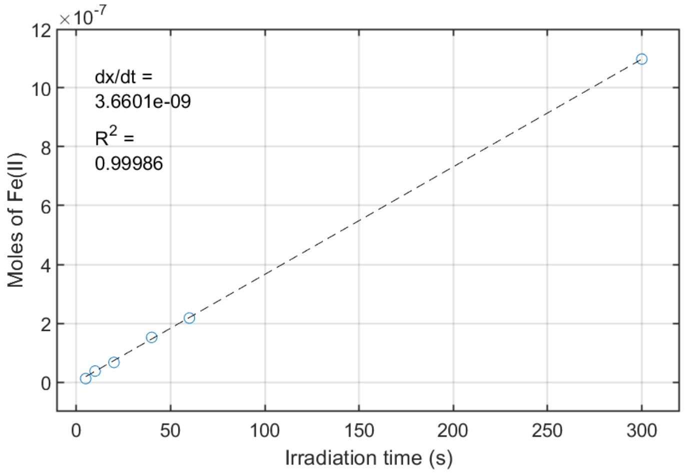

Figure S13. Molar amount of iron(II) formed as a function of time

and from the slope of the fitted data, the photon flux could be calculated according to eq. 4,

$$photon\ flux = \frac{\frac{dx}{dt}}{\phi(456\ nm)*(1-10^{-A(456\ nm)})} \qquad (4)$$

where dx/dt is the rate of formation of iron(II), $\phi(456\ nm)$ is the quantum yield for the ferrioxalate actinometer at 456 nm excitation (0.9) and A(456 nm) is the absorbance of the ferrioxalate actinometer at the irradiation wavelength (0.19).[4] Consequently, the photon flux was determined to be $1.1*10^{-8}$ moles of photons s^{-1}.

The model reaction (prepared using 0.06 mmol *N*-phenyl maleimide, 0.24 mmol *N*-phenyl glycine in 2 mL methanol in a 1x1 cm quartz cuvette) was then irradiated using the identical setup during varying times (3.6 h, 5.3 h, 6.3 h and 7.3 h). The molar amount of product formed was determined using GC-FID with chlorobenzene as the internal standard. The molar amount of product formed was then plotted as a function of the theoretical number of photons absorbed by the reaction mixture (A_{456} = 0.22) for each irradiation time (Figure S14).

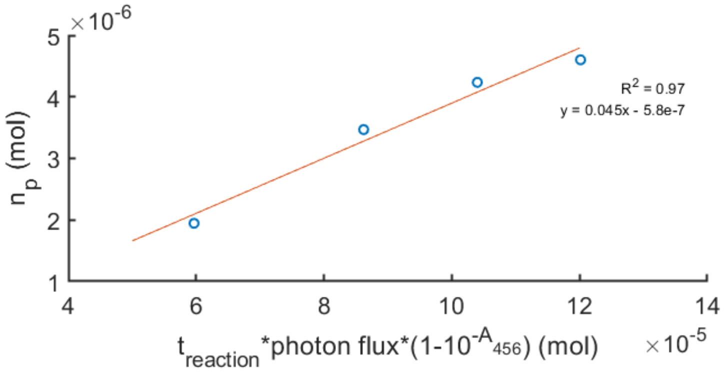

Figure S14. Molar amount of product formed as a function of molar amount of photons absorbed by the reaction mixture.

Using linear fitting, the quantum yield of the reaction (the slope of the line in Figure S14) could be determined to be **0.05**.

1.8 References

(1) Thordarson, P. Determining Association Constants from Titration Experiments in Supramolecular Chemistry. *Chem. Soc. Rev.* **2011**, *40* (3), 1305–1323. https://doi.org/10.1039/C0CS00062K.

(2) Kandukuri, S. R.; Bahamonde, A.; Chatterjee, I.; Jurberg, I. D.; Escudero-Adán, E. C.; Melchiorre, P. X-Ray Characterization of an Electron Donor–Acceptor Complex That Drives the Photochemical Alkylation of Indoles. *Angew. Chemie Int. Ed.* **2015**, *54* (5), 1485–1489. https://doi.org/10.1002/anie.201409529.

(3) Hansch, C.; Leo, A.; Taft, R. W. A Survey of Hammett Substituent Constants and Resonance and Field Parameters. *Chem. Rev.* **1991**, *91* (2), 165–195. https://doi.org/10.1021/cr00002a004.

(4) Rabani, J.; Mamane, H.; Pousty, D.; Bolton, J. R. Practical Chemical Actinometry—A Review. *Photochem. Photobiol.* **2021**, *97* (5), 873–902. https://doi.org/https://doi.org/10.1111/php.13429.

1.9 NMR spectra

Starting materials

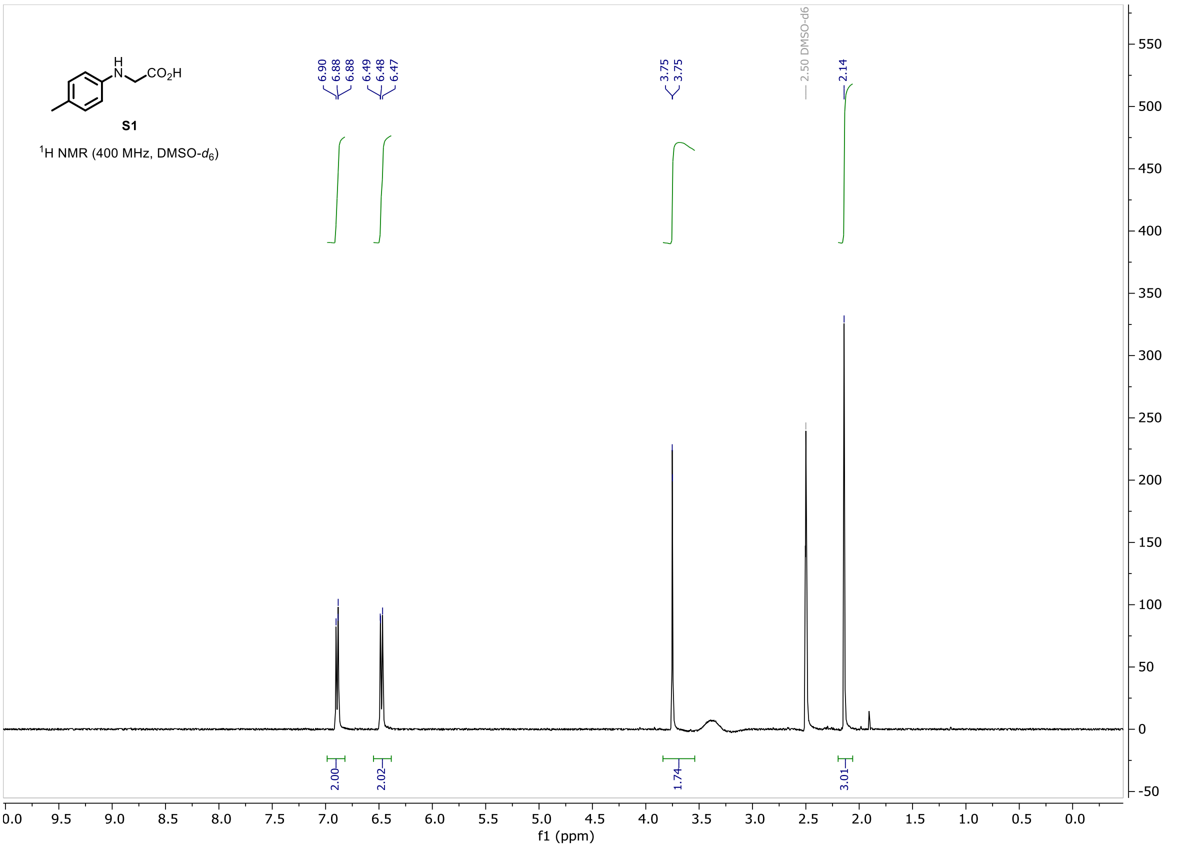

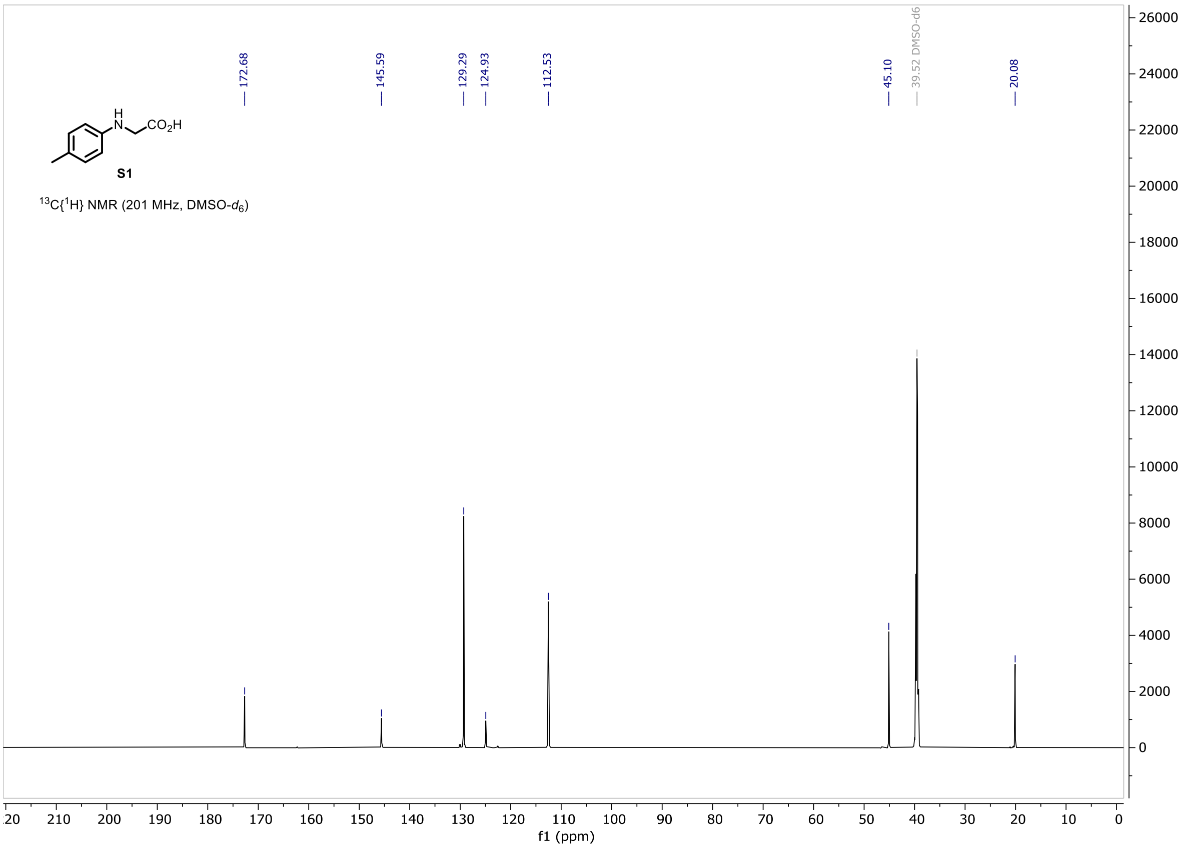

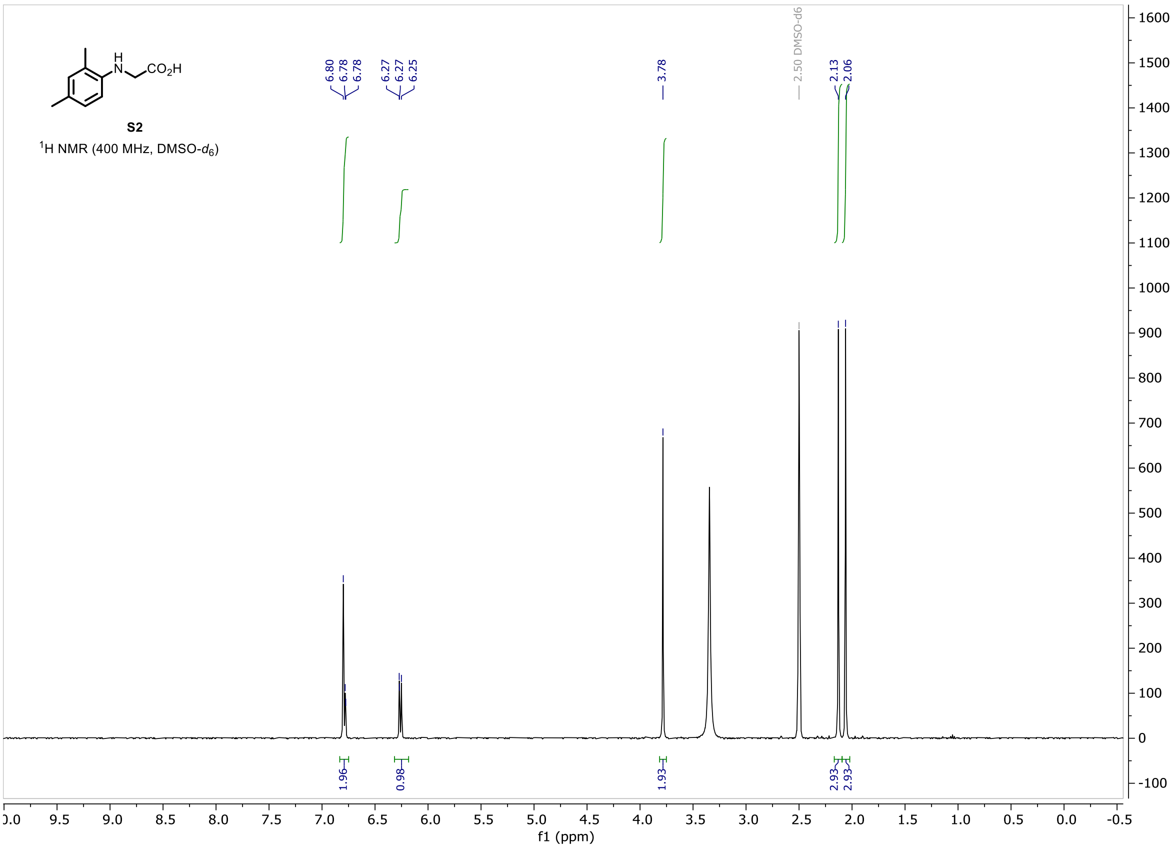

S2
^{1}H NMR (400 MHz, DMSO-d_6)
6.80
6.78
6.78
6.27
6.27
6.25
3.78
2.50 DMSO-d6
2.13
2.06
1.96
0.98
1.93
2.93
2.93
f1 (ppm)

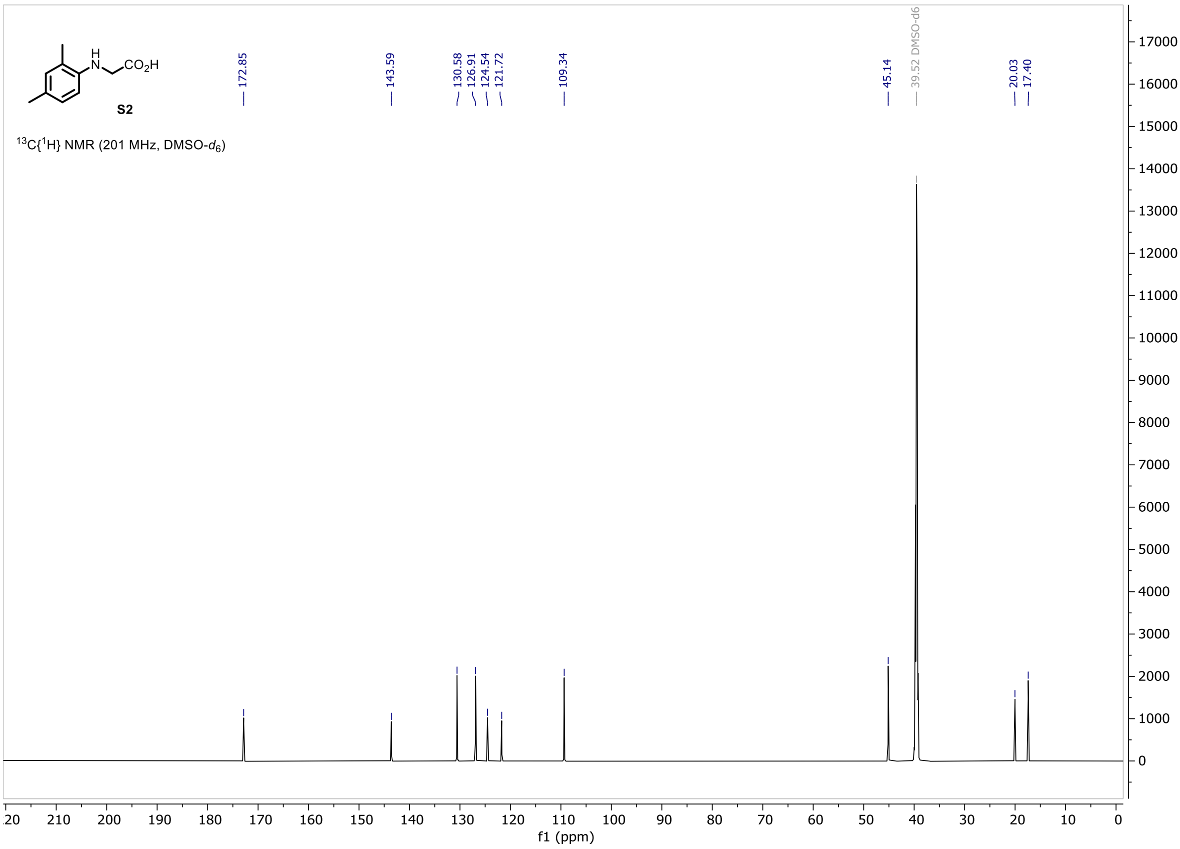

S2
^{13}C{^{1}H} NMR (201 MHz, DMSO-d_6)
172.85
143.59
130.58
126.91
124.54
121.72
109.34
45.14
39.52 DMSO-d6
20.03
17.40
f1 (ppm)

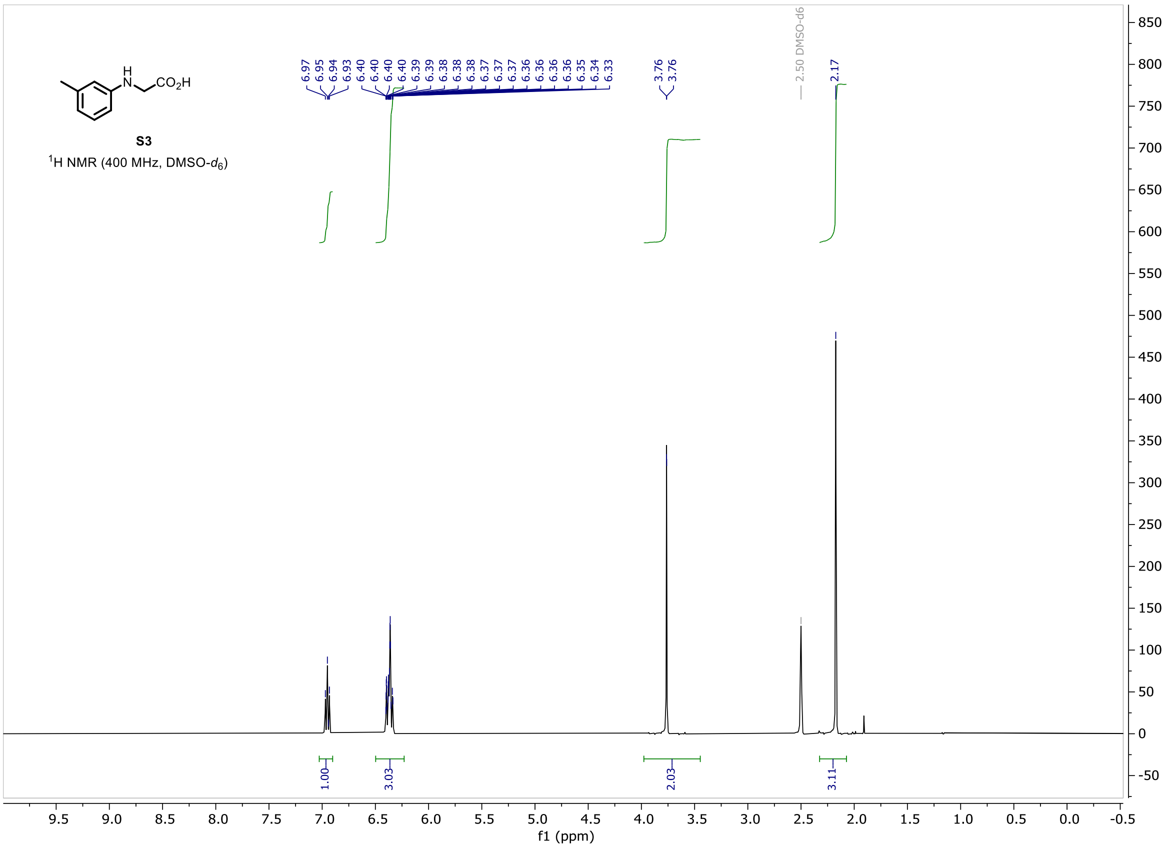
S3
^{1}H NMR (400 MHz, DMSO-d_6)
6.97
6.95
6.94
6.93
6.40
6.40
6.40
6.40
6.39
6.39
6.38
6.38
6.38
6.37
6.37
6.37
6.36
6.36
6.36
6.36
6.35
6.34
6.33
3.76
3.76
2.50 DMSO-d6
2.17
1.00
3.03
2.03
3.11
f1 (ppm)

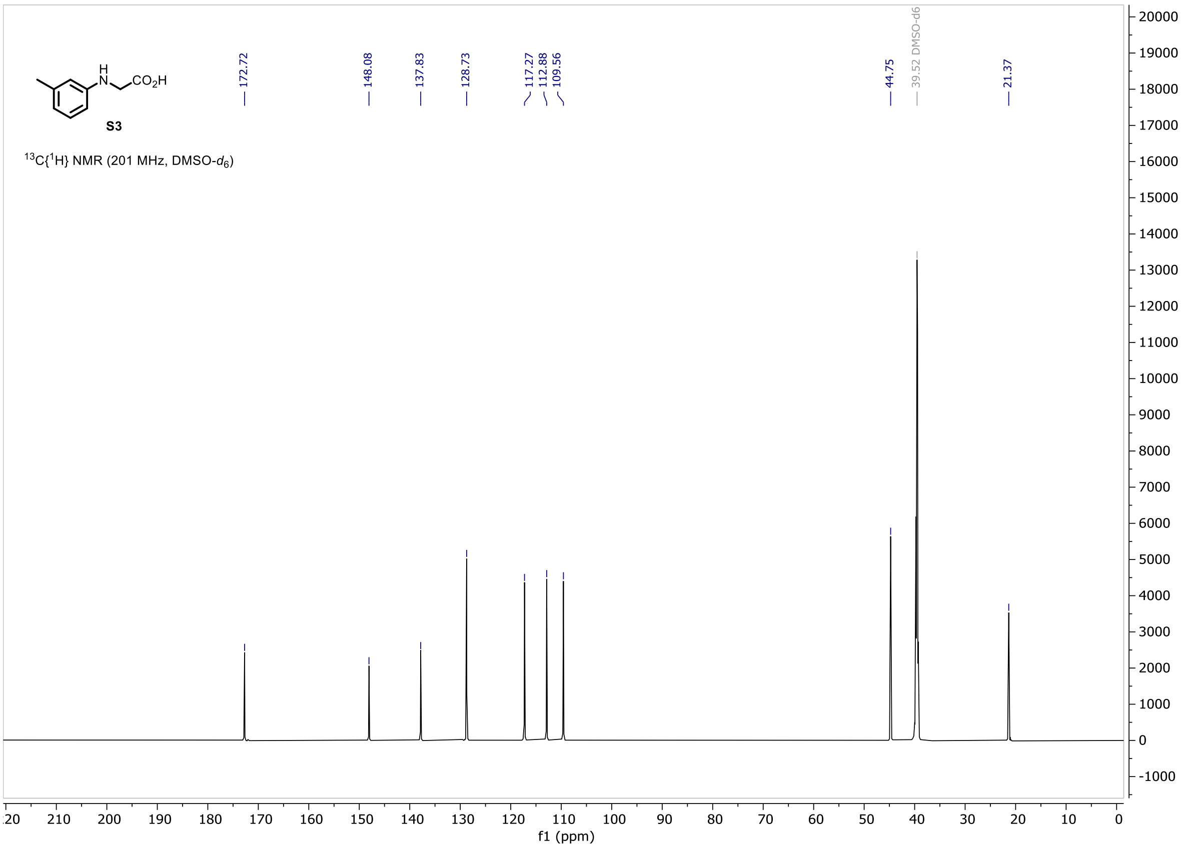
S3
^{13}C{^{1}H} NMR (201 MHz, DMSO-d_6)
172.72
148.08
137.83
128.73
117.27
112.88
109.56
44.75
39.52 DMSO-d6
21.37
f1 (ppm)

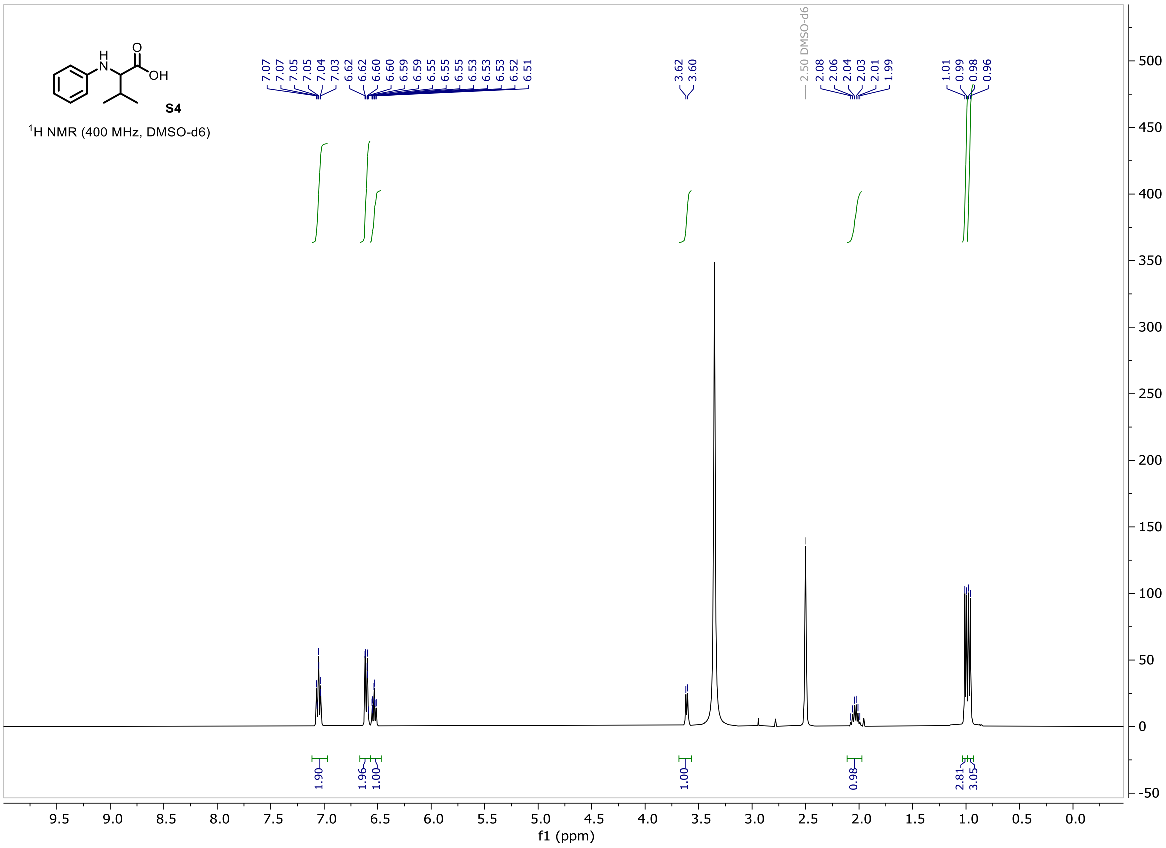
S4
1H NMR (400 MHz, DMSO-d6)
7.07
7.07
7.05
7.04
7.03
6.62
6.62
6.60
6.60
6.59
6.59
6.55
6.55
6.53
6.53
6.53
6.52
6.51
3.62
3.60
2.50 DMSO-d6
2.08
2.06
2.04
2.03
2.01
1.99
1.01
0.99
0.98
0.96
1.90
1.96
1.00
1.00
0.98
2.81
3.05
f1 (ppm)

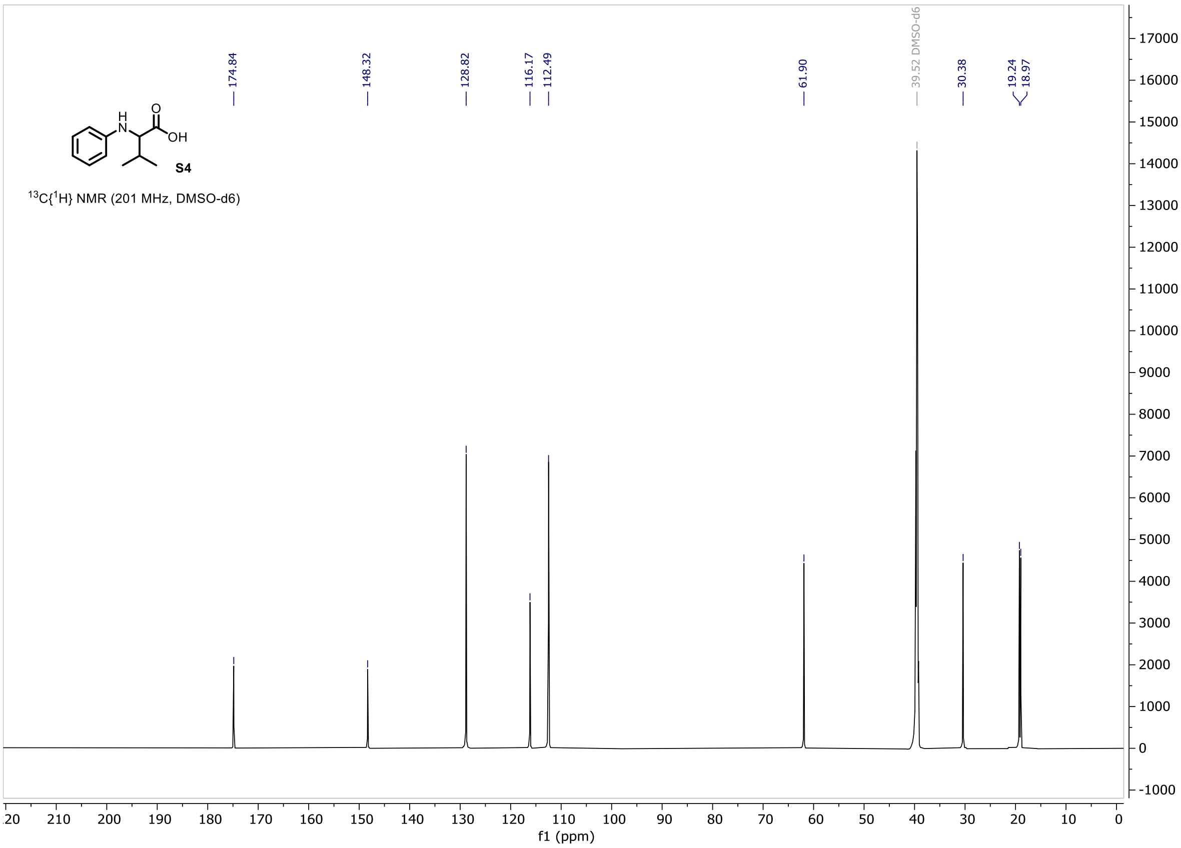
S4
13C{1H} NMR (201 MHz, DMSO-d6)
174.84
148.32
128.82
116.17
112.49
61.90
39.52 DMSO-d6
30.38
19.24
18.97
f1 (ppm)

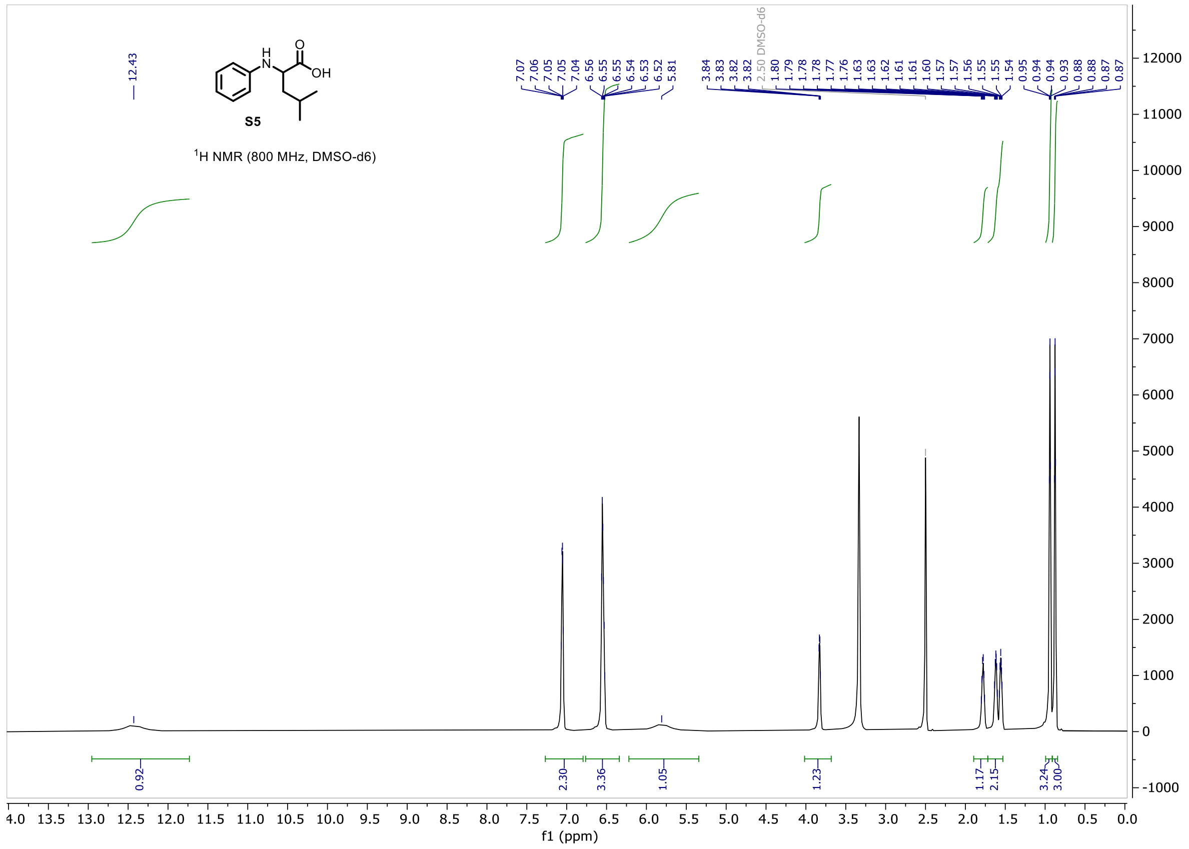
S5
^{1}H NMR (800 MHz, DMSO-d6)
f1 (ppm)

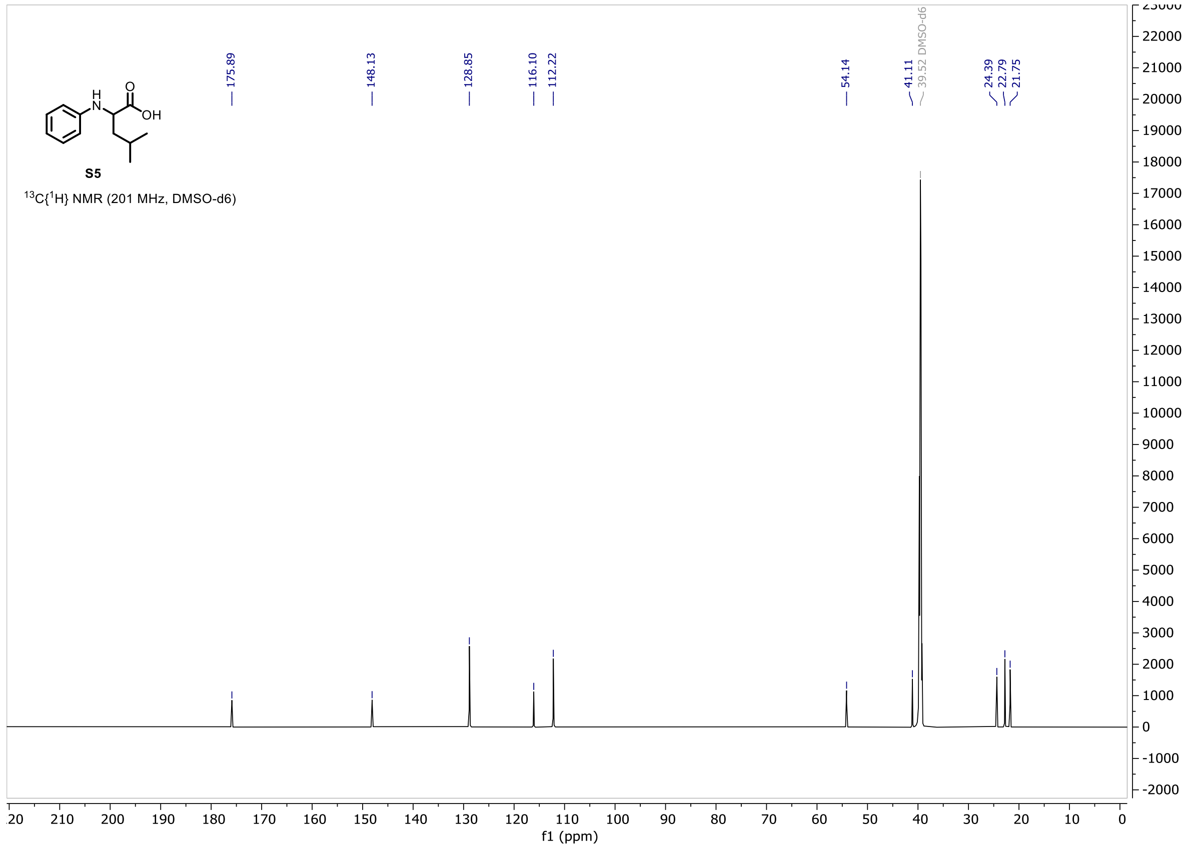
S5
^{13}C{^{1}H} NMR (201 MHz, DMSO-d6)
f1 (ppm)

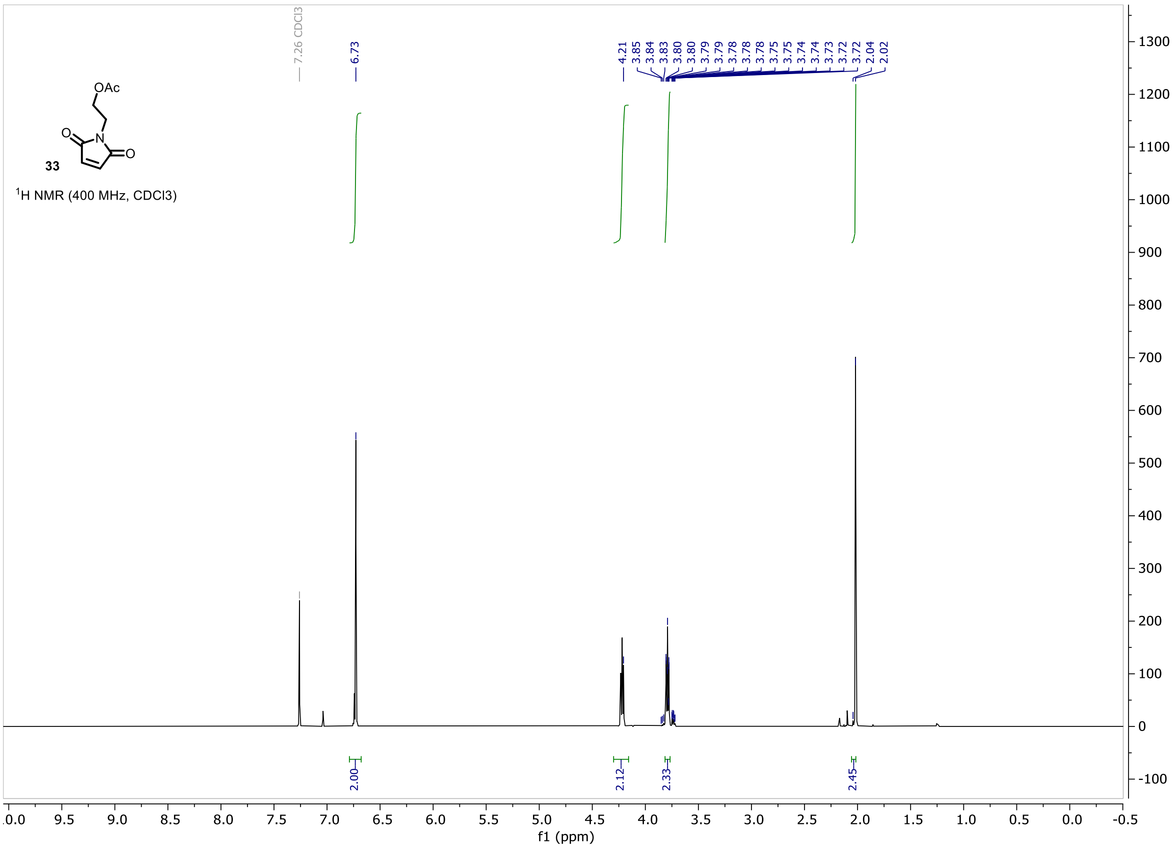

OAc
33
1H NMR (400 MHz, CDCl3)
7.26 CDCl3
6.73
4.21
2.04
2.02
2.00
2.12
2.33
2.45
f1 (ppm)

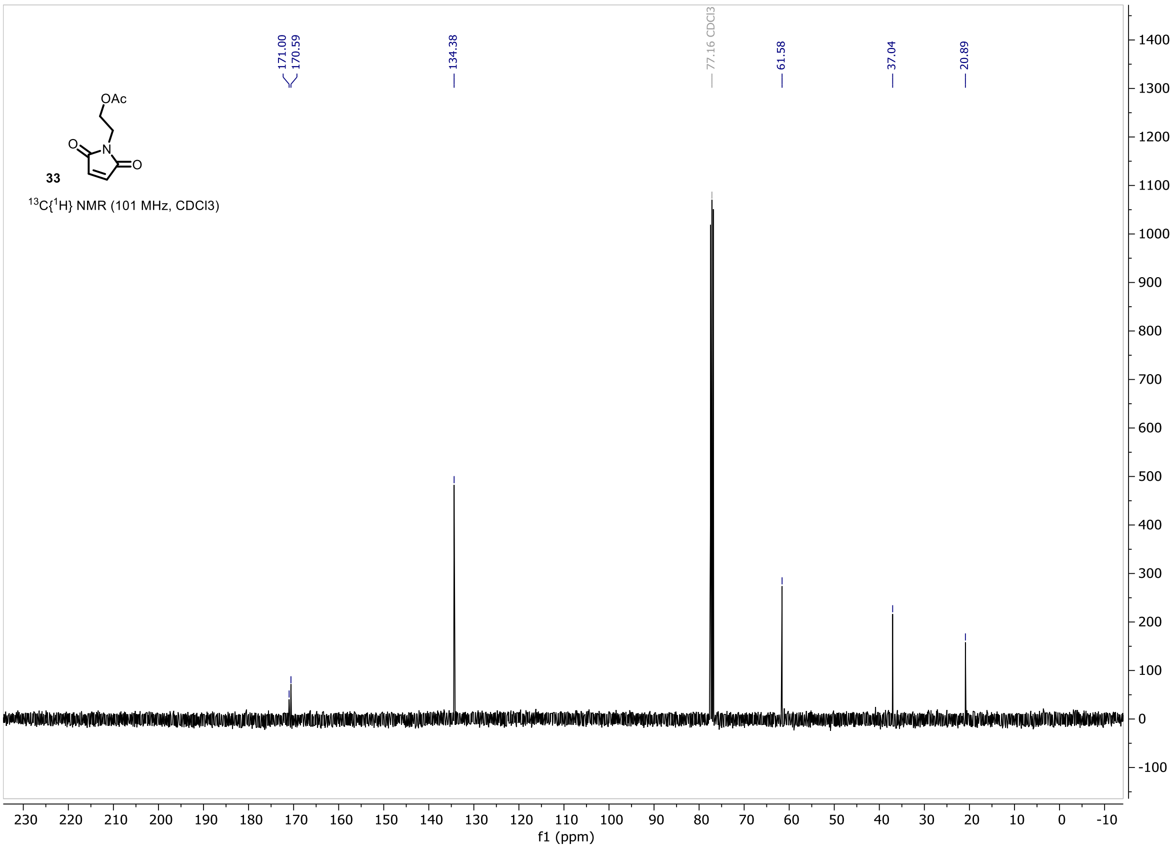

OAc
33
13C{1H} NMR (101 MHz, CDCl3)
171.00
170.59
134.38
77.16 CDCl3
61.58
37.04
20.89
f1 (ppm)

Tetrahydroquionoline products

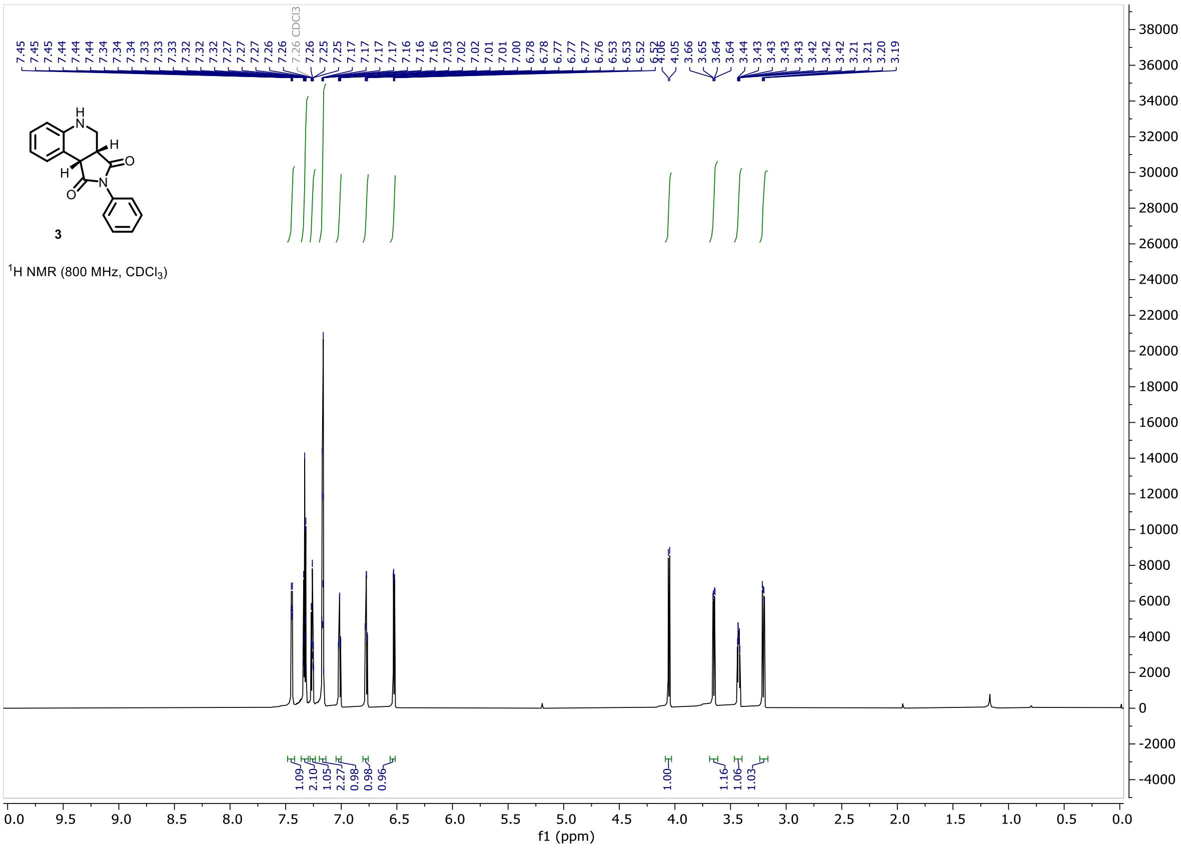

3

^{1}H NMR (800 MHz, $CDCl_3$)

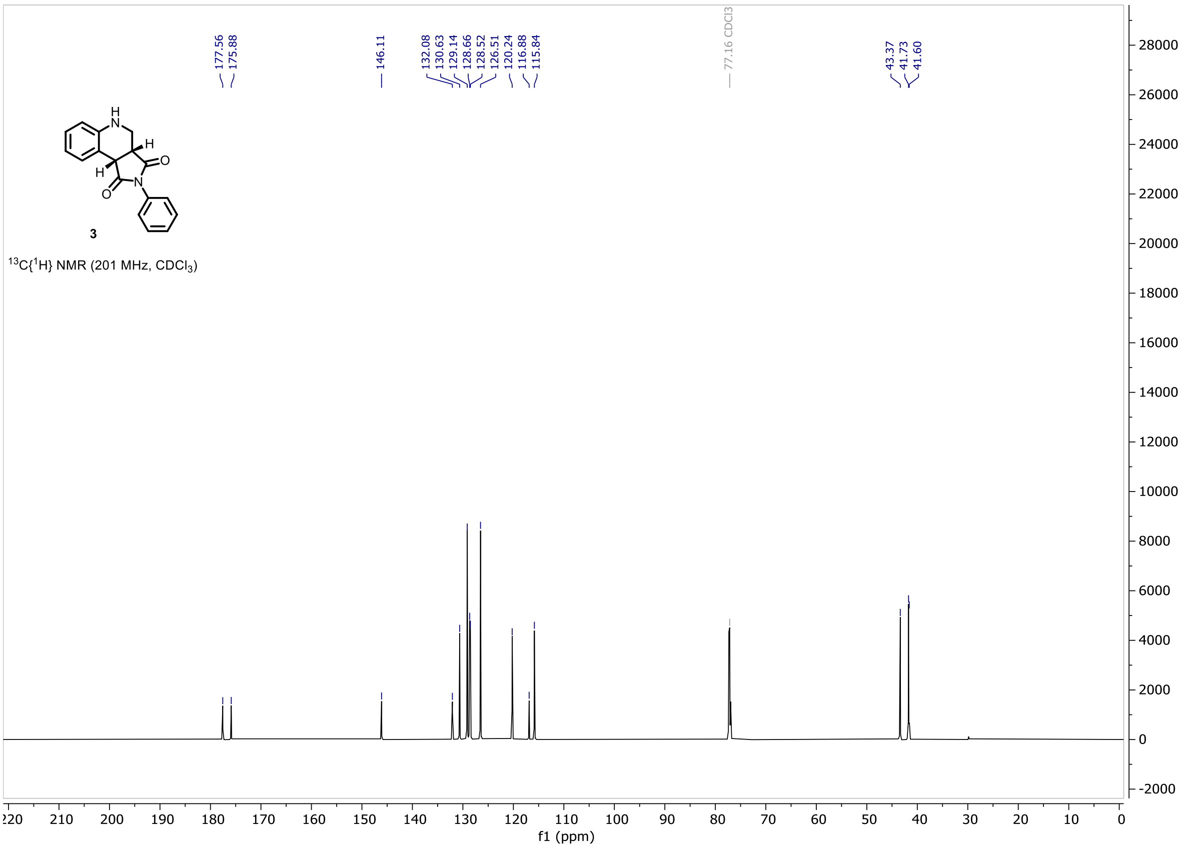

3

^{13}C{^{1}H} NMR (201 MHz, $CDCl_3$)

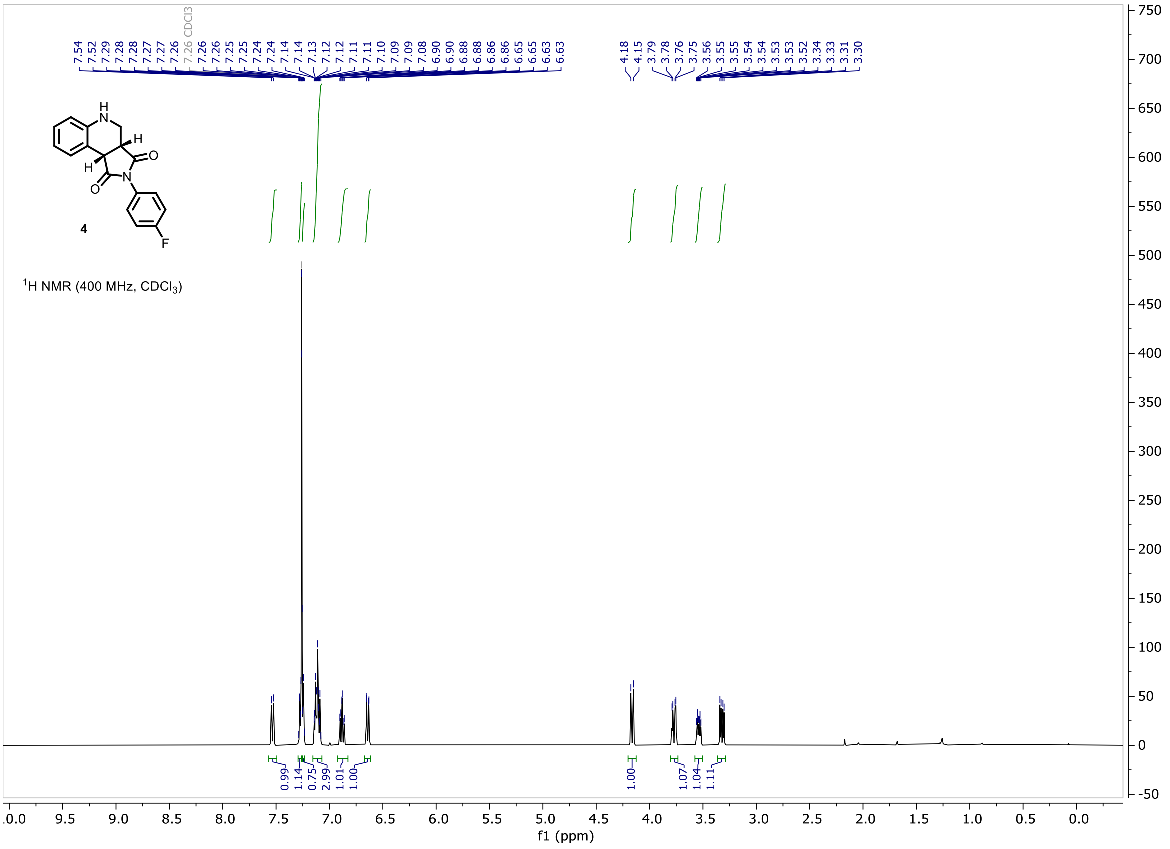
4
1H NMR (400 MHz, CDCl3)
f1 (ppm)

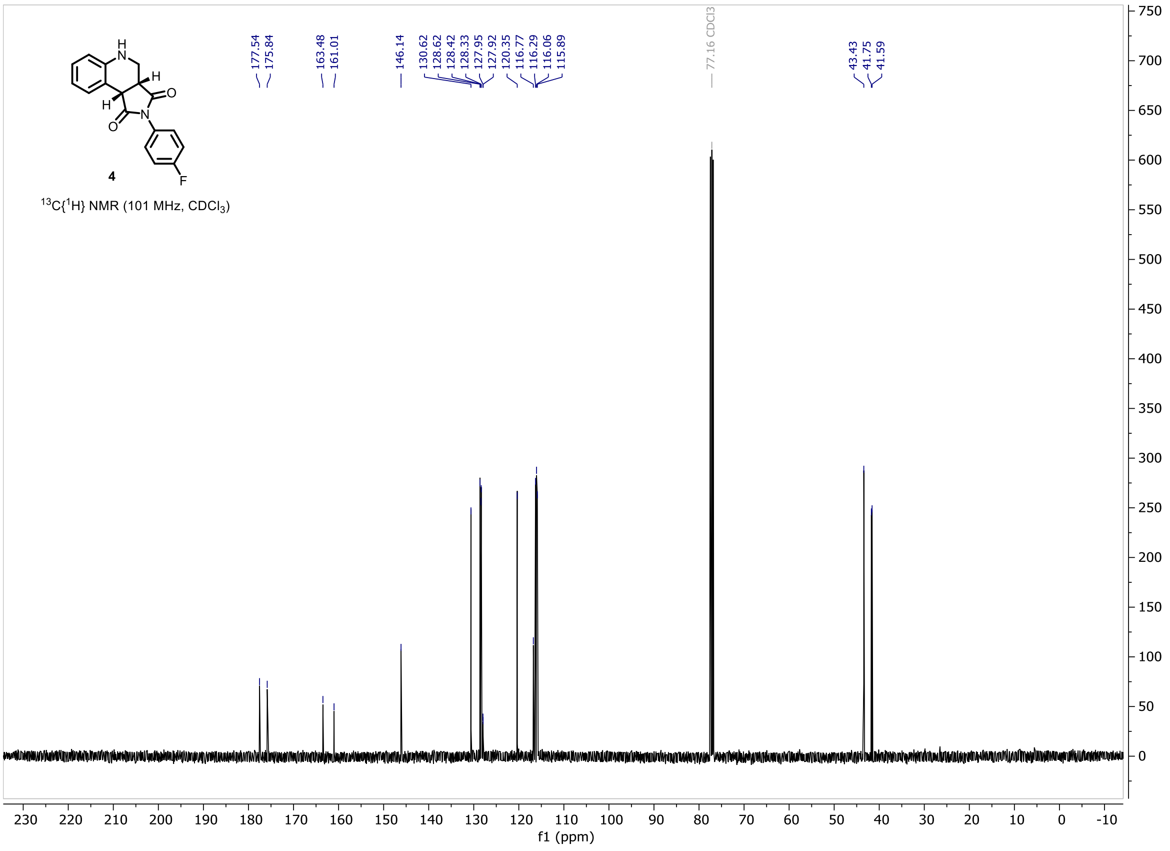
4
13C{1H} NMR (101 MHz, CDCl3)
f1 (ppm)

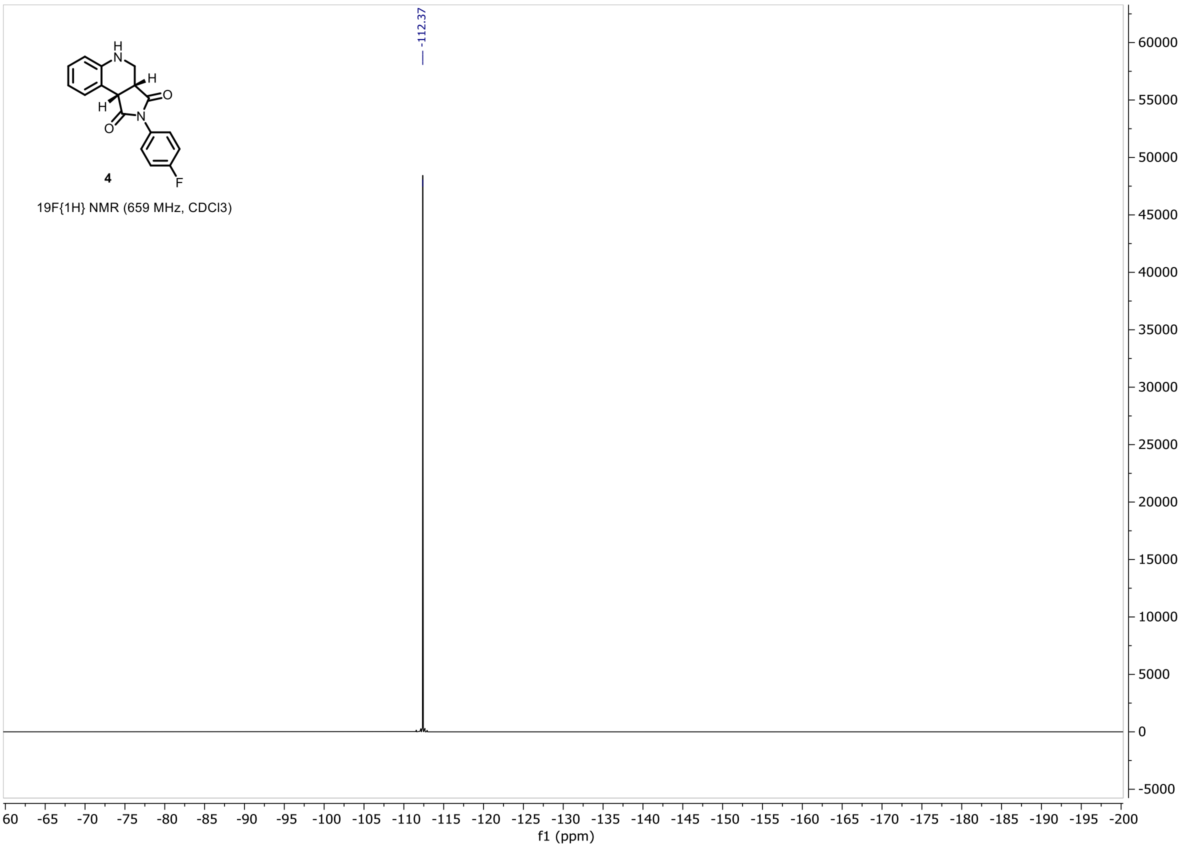
-112.37
4
19F{1H} NMR (659 MHz, CDCl3)
60000
55000
50000
45000
40000
35000
30000
25000
20000
15000
10000
5000
0
-5000
60 -65 -70 -75 -80 -85 -90 -95 -100 -105 -110 -115 -120 -125 -130 -135 -140 -145 -150 -155 -160 -165 -170 -175 -180 -185 -190 -195 -200
f1 (ppm)

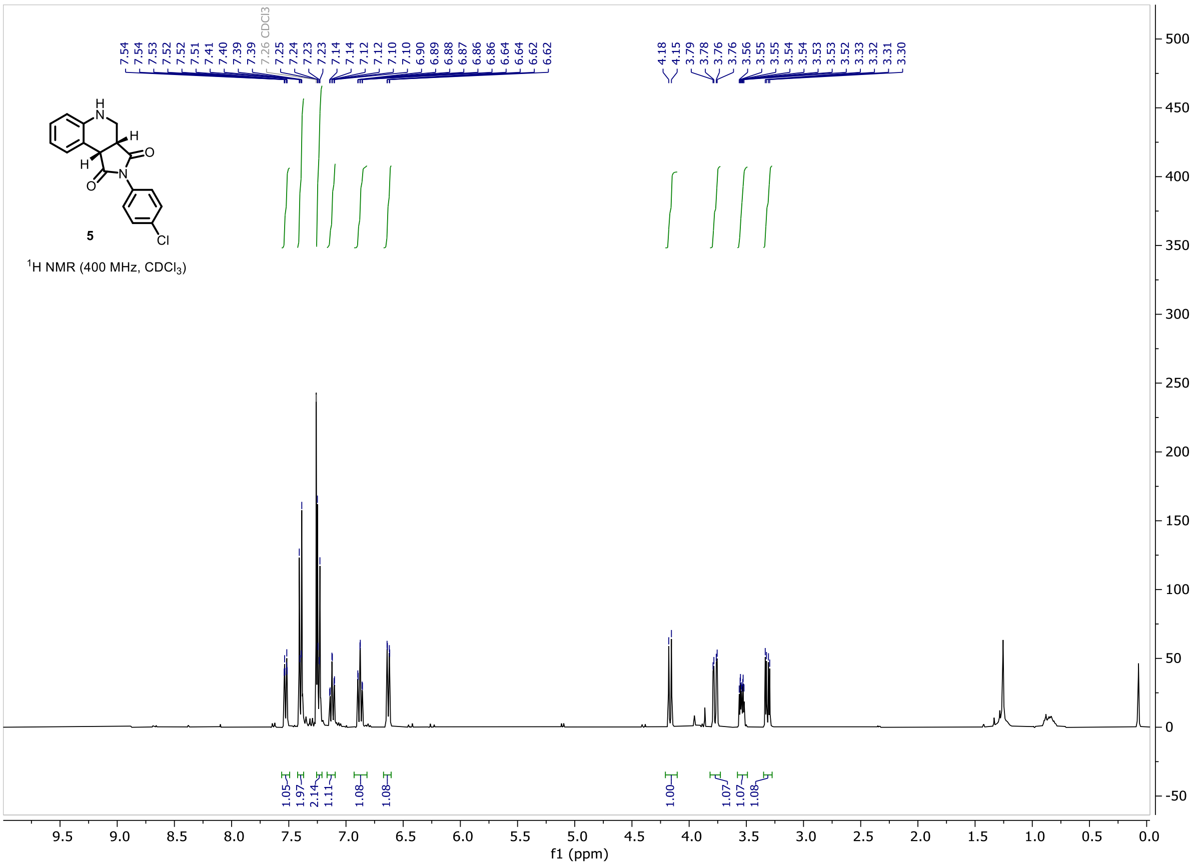
5
^{1}H NMR (400 MHz, CDCl$_3$)
f1 (ppm)

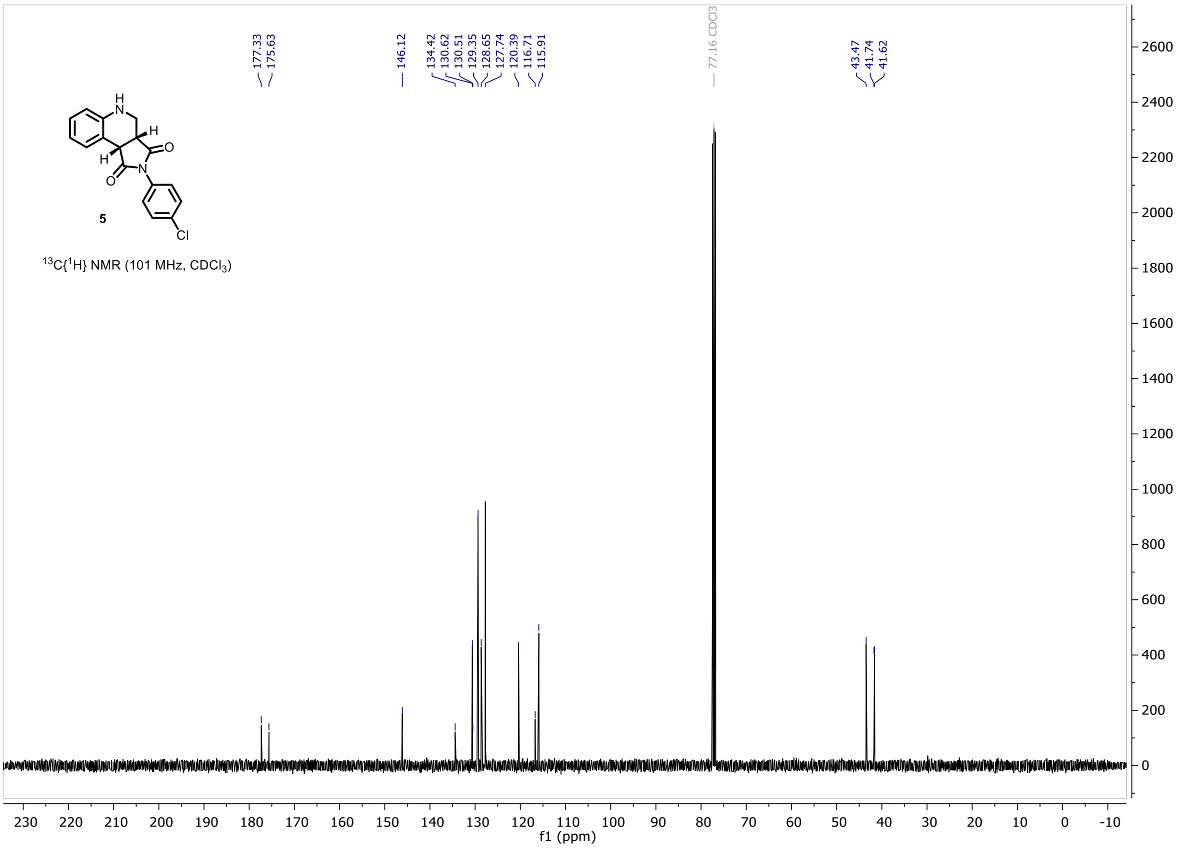
5
^{13}C{^{1}H} NMR (101 MHz, CDCl$_3$)
f1 (ppm)

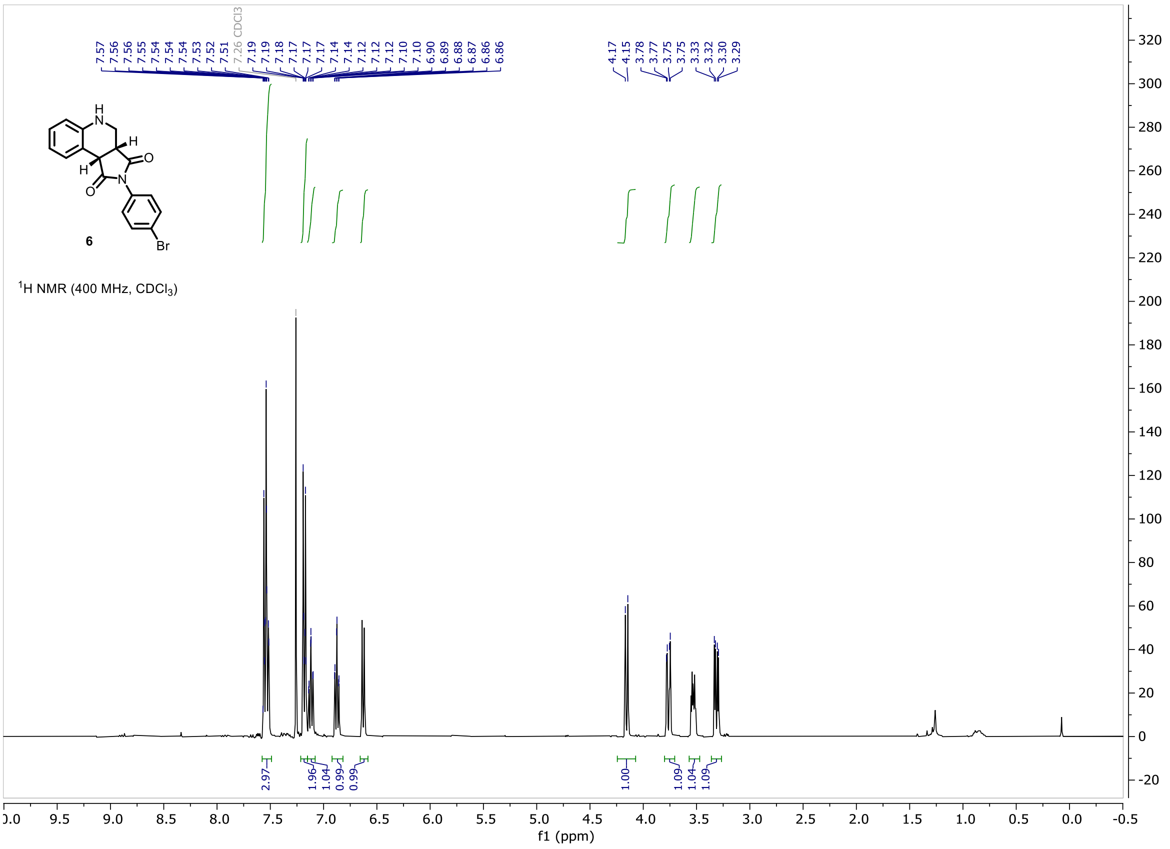

6
^{1}H NMR (400 MHz, CDCl$_3$)
7.26 CDCl3
f1 (ppm)

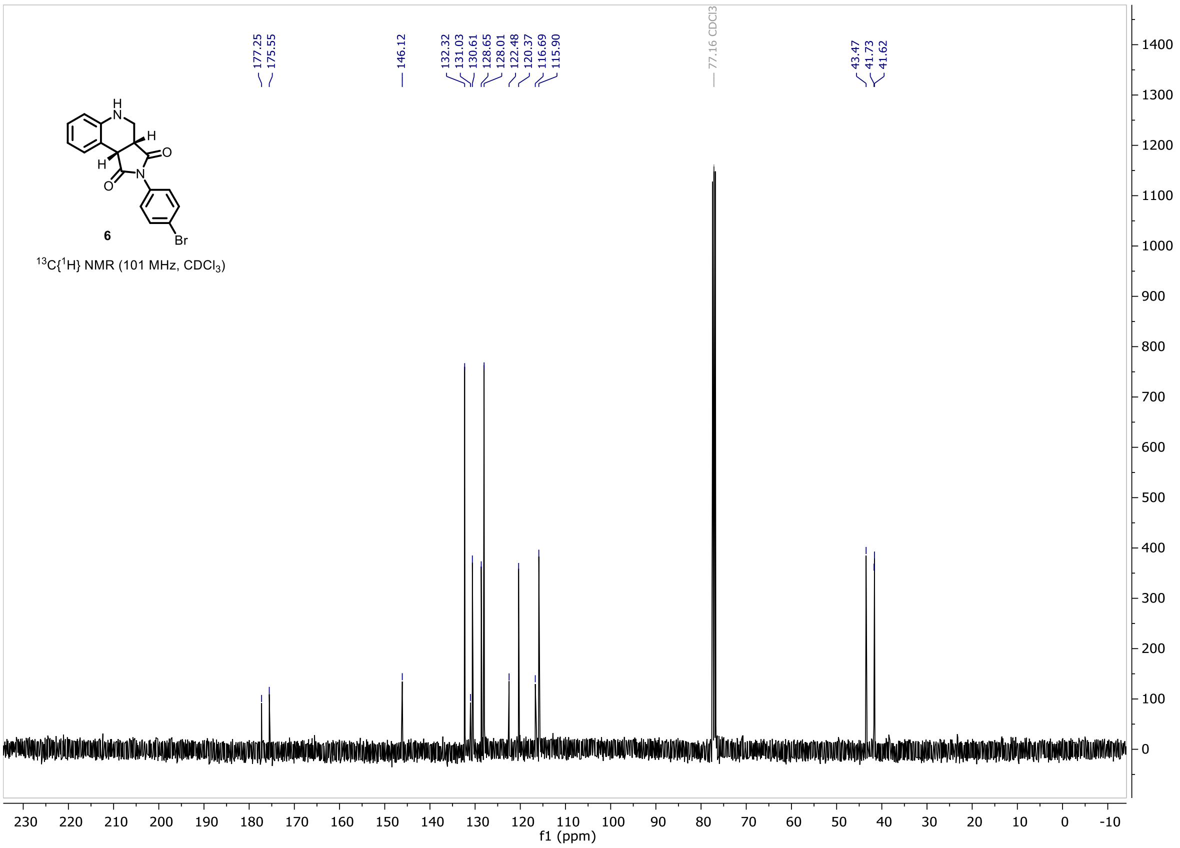

6
^{13}C{^{1}H} NMR (101 MHz, CDCl$_3$)
177.25
175.55
146.12
132.32
131.03
130.61
128.65
128.01
122.48
120.37
116.69
115.90
77.16 CDCl3
43.47
41.73
41.62
f1 (ppm)

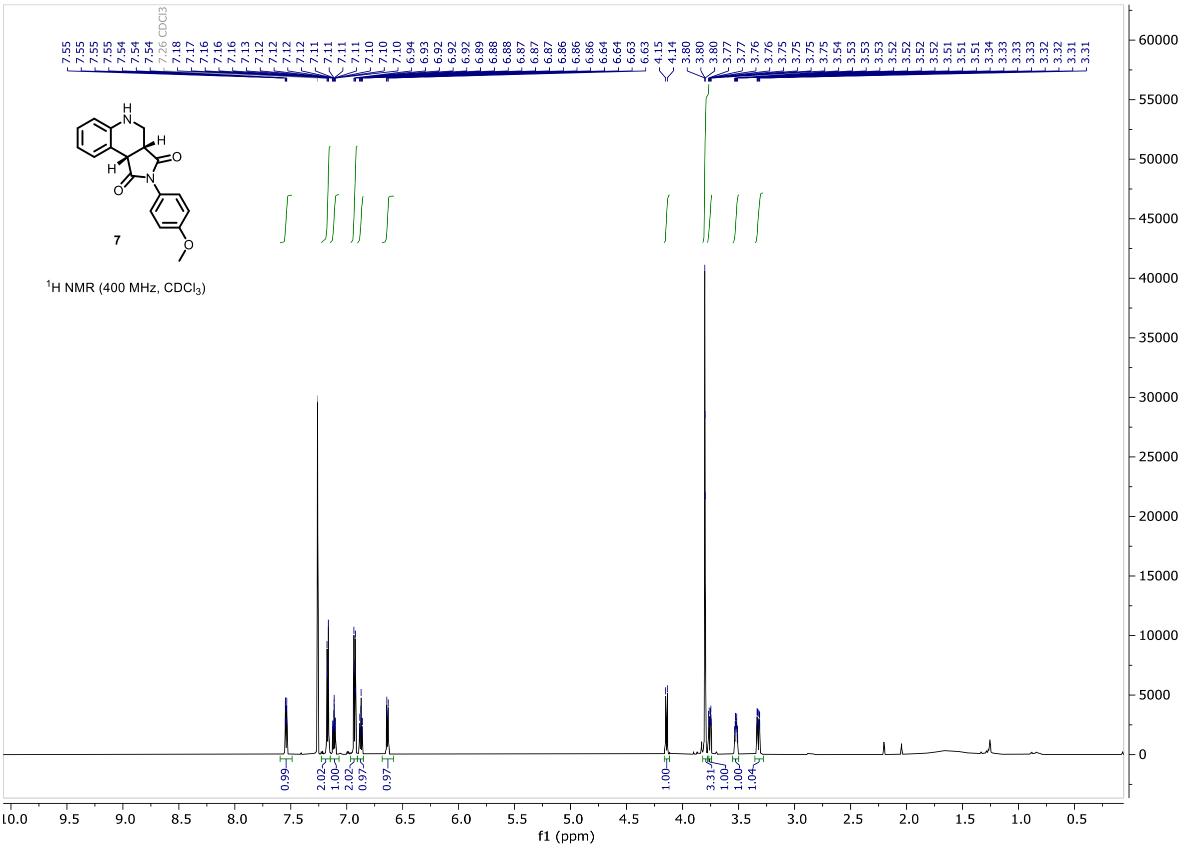
7.55 7.55 7.55 7.54 7.54 7.54 7.26 CDCl3 7.18 7.17 7.16 7.16 7.13 7.12 7.12 7.12 7.11 7.11 7.11 7.11 7.10 7.10 6.94 6.93 6.92 6.92 6.89 6.88 6.88 6.87 6.87 6.86 6.86 6.86 6.64 6.64 6.63 4.15 4.14 3.80 3.80 3.80 3.77 3.77 3.76 3.76 3.75 3.75 3.75 3.54 3.53 3.53 3.53 3.52 3.52 3.52 3.51 3.51 3.51 3.34 3.33 3.33 3.33 3.32 3.32 3.31 3.31
H
N
H
O
H
O
N
O
7
^{1}H NMR (400 MHz, CDCl$_3$)
0.99 2.02 1.00 2.02 0.97 0.97 1.00 3.31 1.00 1.00 1.04
f1 (ppm)

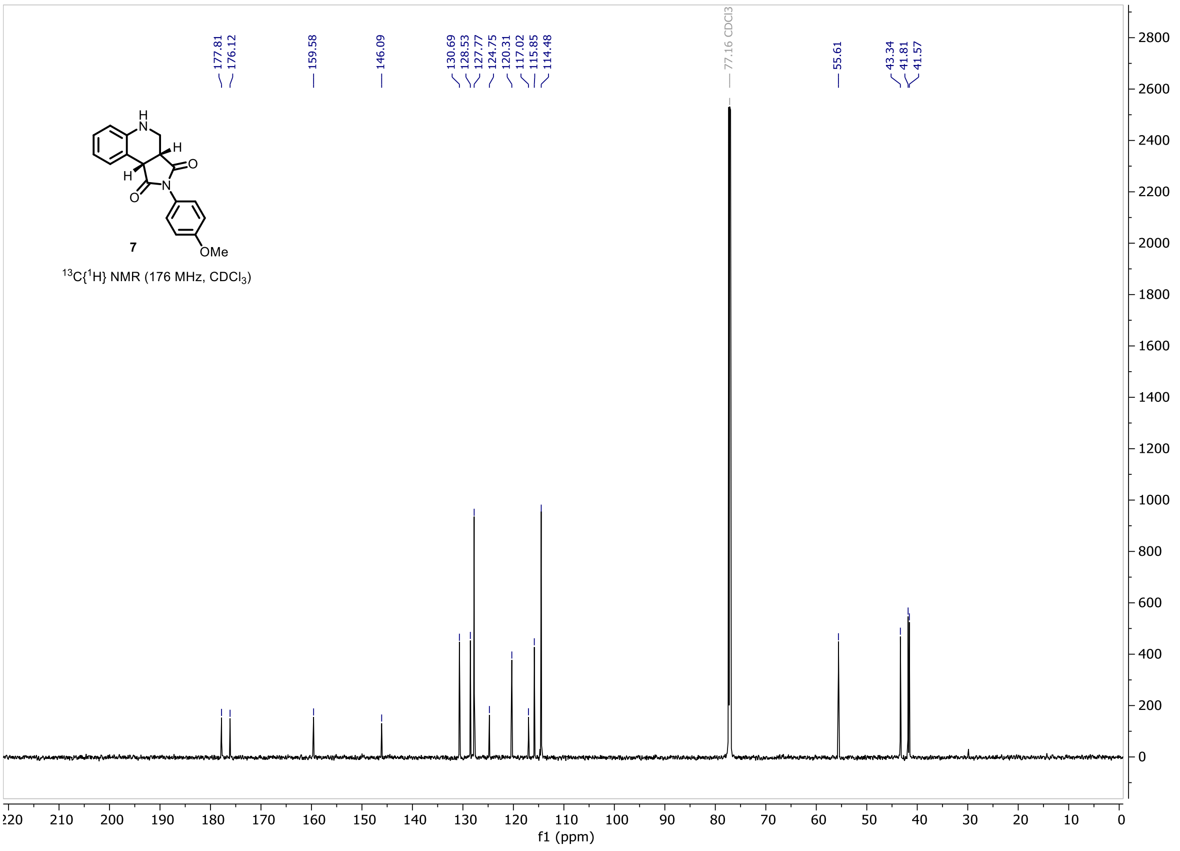
177.81 176.12 159.58 146.09 130.69 128.53 127.77 124.75 120.31 117.02 115.85 114.48 77.16 CDCl3 55.61 43.34 41.81 41.57
H
N
H
O
H
O
N
7
OMe
^{13}C{^{1}H} NMR (176 MHz, CDCl$_3$)
f1 (ppm)

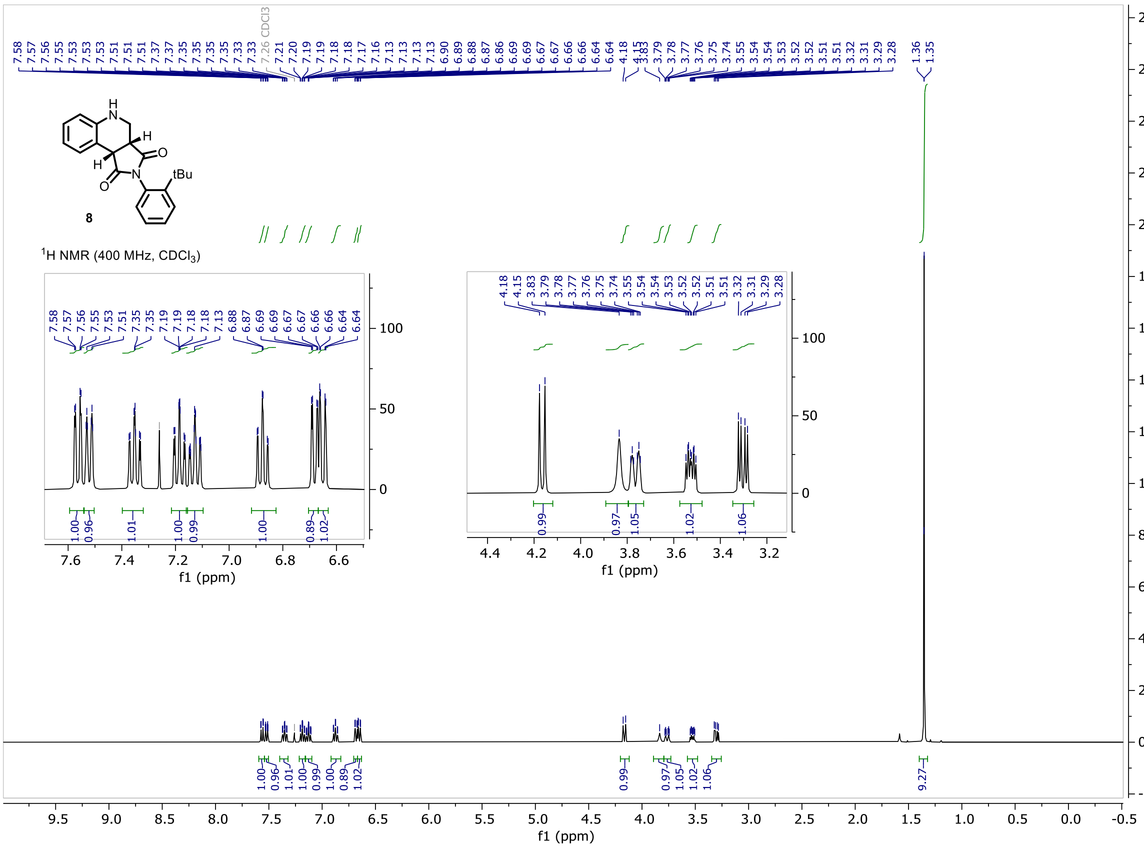

8
tBu
^{1}H NMR (400 MHz, CDCl$_3$)
7.26 CDCl3
1.36
1.35
9.27
f1 (ppm)

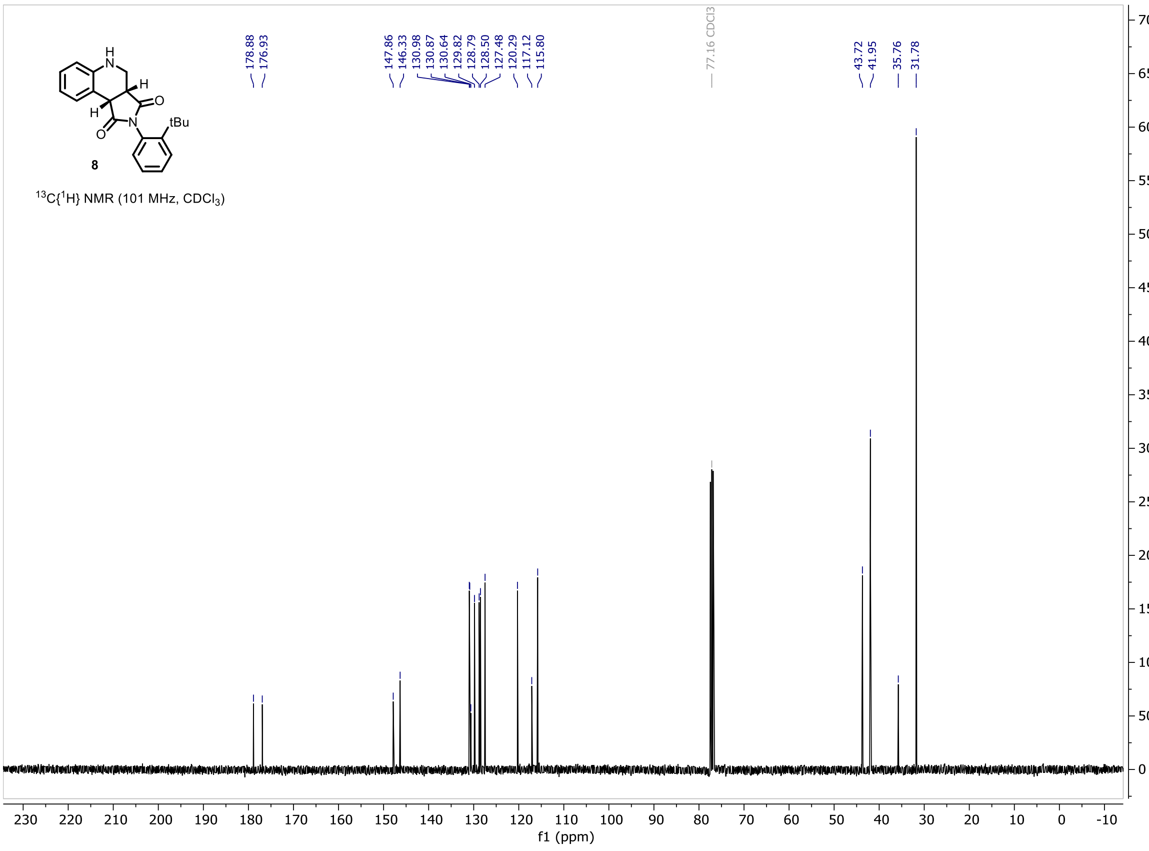

8
tBu
^{13}C{^{1}H} NMR (101 MHz, CDCl$_3$)
178.88
176.93
147.86
146.33
130.98
130.87
130.64
129.82
128.79
128.50
127.48
120.29
117.12
115.80
77.16 CDCl3
43.72
41.95
35.76
31.78
f1 (ppm)

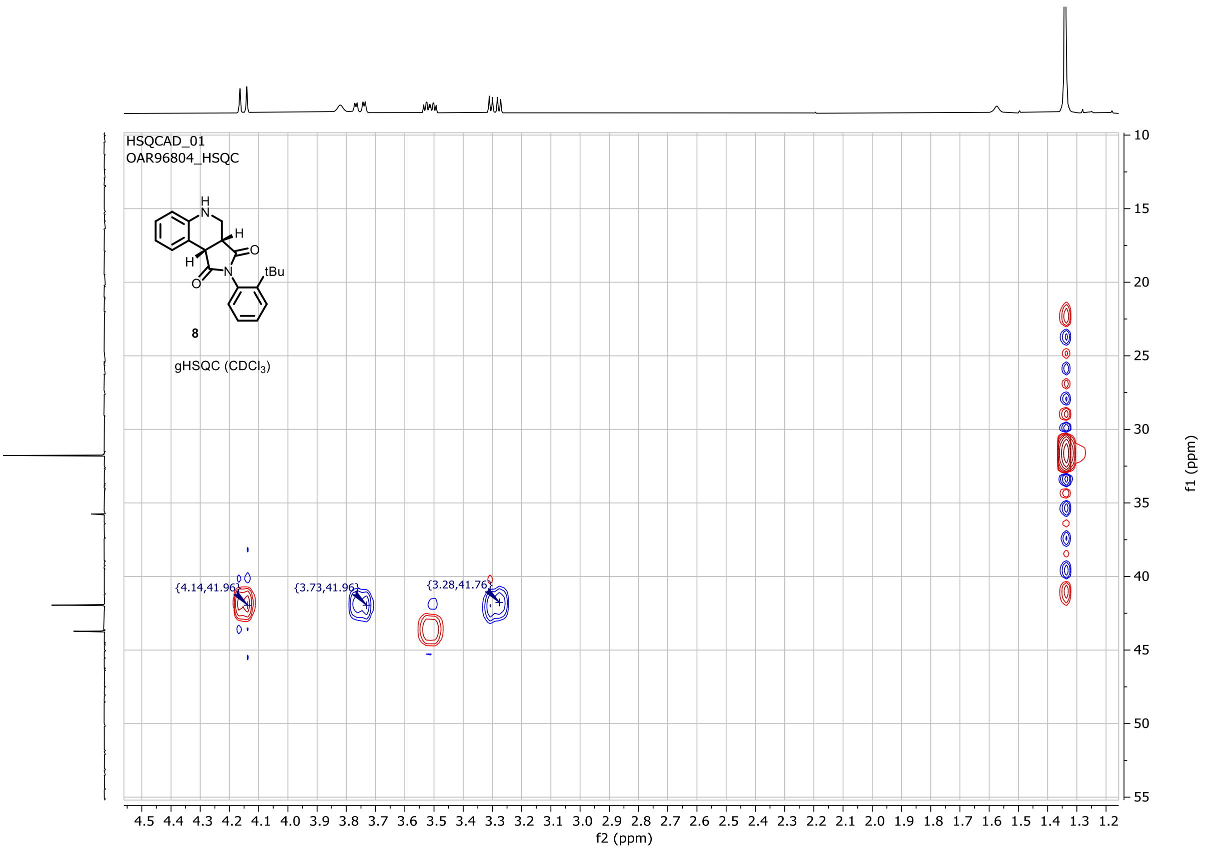
HSQCAD_01
OAR96804_HSQC
8
gHSQC (CDCl3)
{4.14,41.96}
{3.73,41.96}
{3.28,41.76}
f1 (ppm)
f2 (ppm)

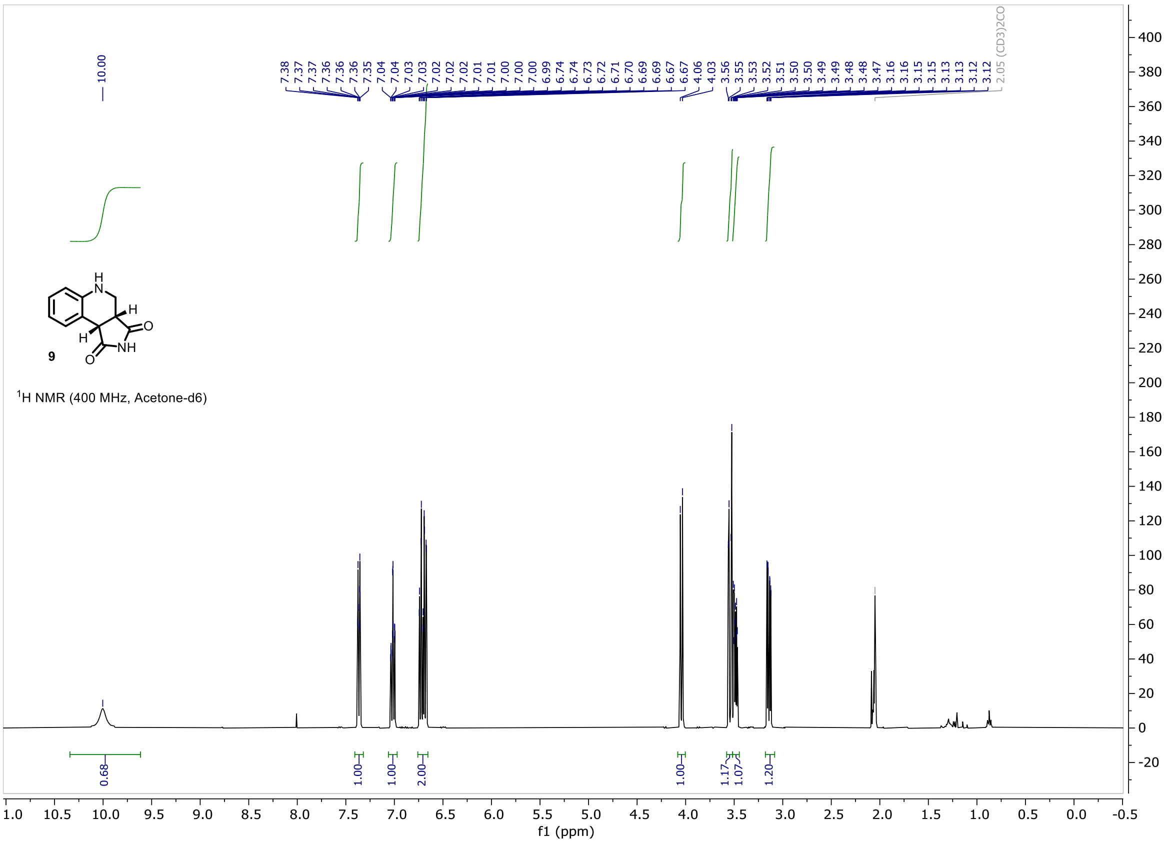

10.00
7.38 7.37 7.37 7.36 7.36 7.35 7.04 7.04 7.03 7.03 7.02 7.02 7.01 7.01 7.00 7.00 7.00 6.99 6.74 6.74 6.73 6.72 6.71 6.70 6.69 6.69 6.67 6.67 4.06 4.03 3.56 3.55 3.53 3.52 3.51 3.50 3.50 3.49 3.49 3.48 3.48 3.47 3.16 3.16 3.15 3.15 3.13 3.13 3.12 3.12
2.05 (CD3)2CO
9
^{1}H NMR (400 MHz, Acetone-d6)
0.68 1.00 1.00 2.00 1.00 1.17 1.07 1.20
f1 (ppm)

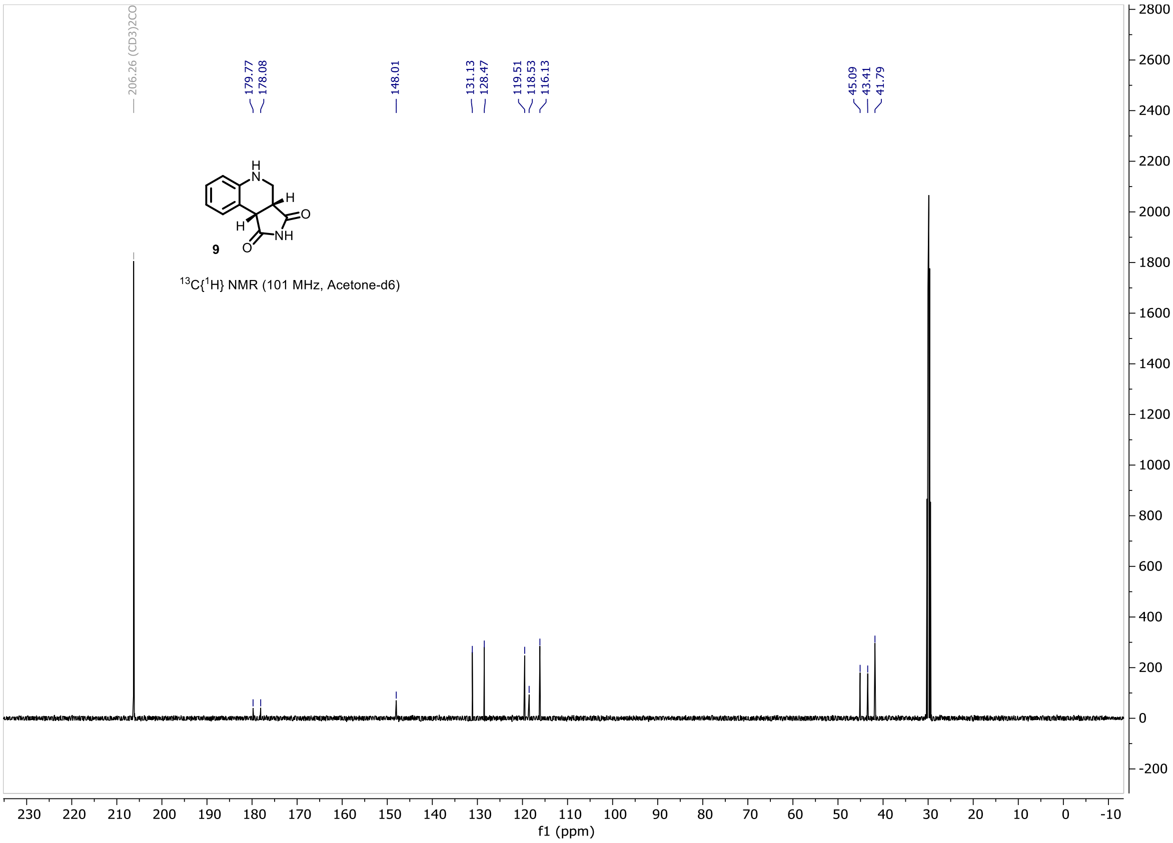

206.26 (CD3)2CO
179.77 178.08
148.01
131.13 128.47
119.51 118.53 116.13
45.09 43.41 41.79
9
^{13}C{^{1}H} NMR (101 MHz, Acetone-d6)
f1 (ppm)

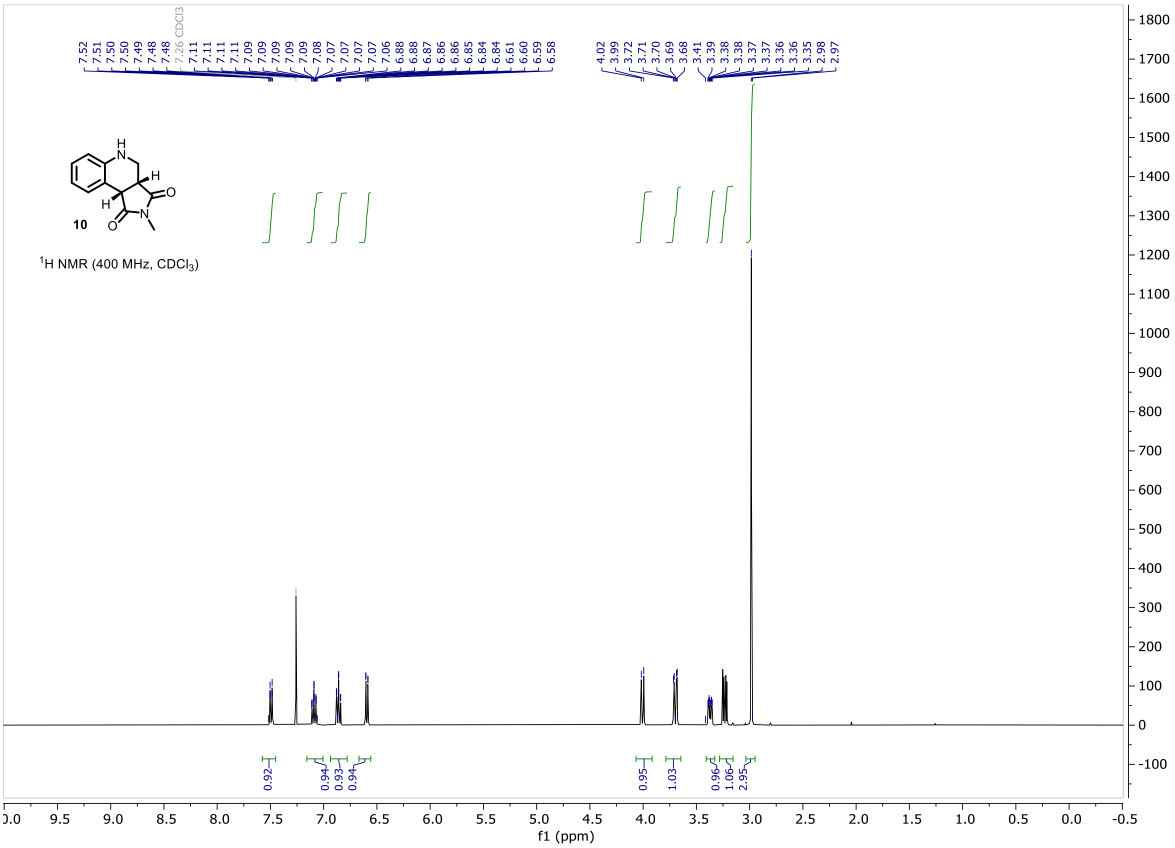
10
1H NMR (400 MHz, CDCl3)
7.52 7.51 7.50 7.50 7.49 7.48 7.48 7.26 CDCl3 7.11 7.11 7.11 7.11 7.11 7.09 7.09 7.09 7.09 7.09 7.08 7.07 7.07 7.07 7.07 7.06 6.88 6.88 6.87 6.86 6.86 6.85 6.84 6.84 6.61 6.60 6.59 6.58
4.02 3.99 3.72 3.71 3.70 3.69 3.68 3.41 3.39 3.38 3.38 3.37 3.37 3.36 3.36 3.35 2.98 2.97
0.92 0.94 0.93 0.94 0.95 1.03 0.96 1.06 2.95
f1 (ppm)

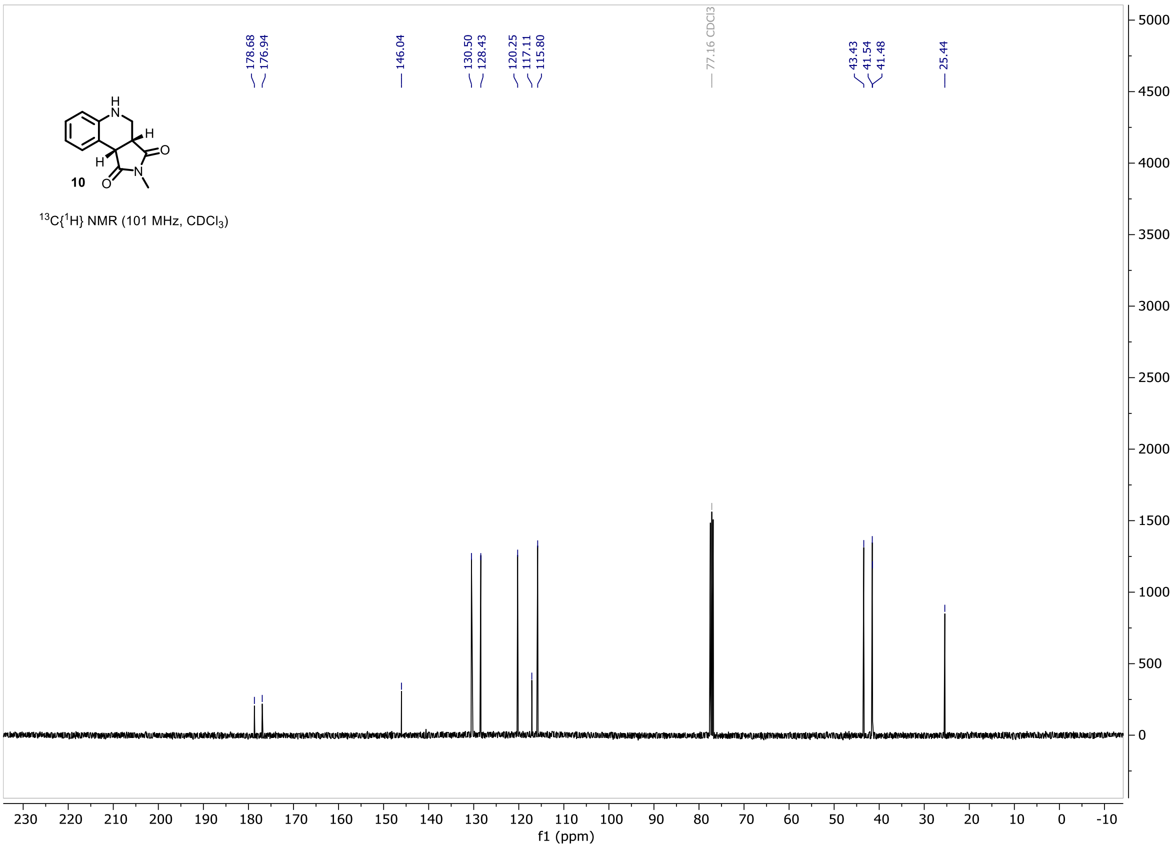
10
13C{1H} NMR (101 MHz, CDCl3)
178.68 176.94 146.04 130.50 128.43 120.25 117.11 115.80 77.16 CDCl3 43.43 41.54 41.48 25.44
f1 (ppm)

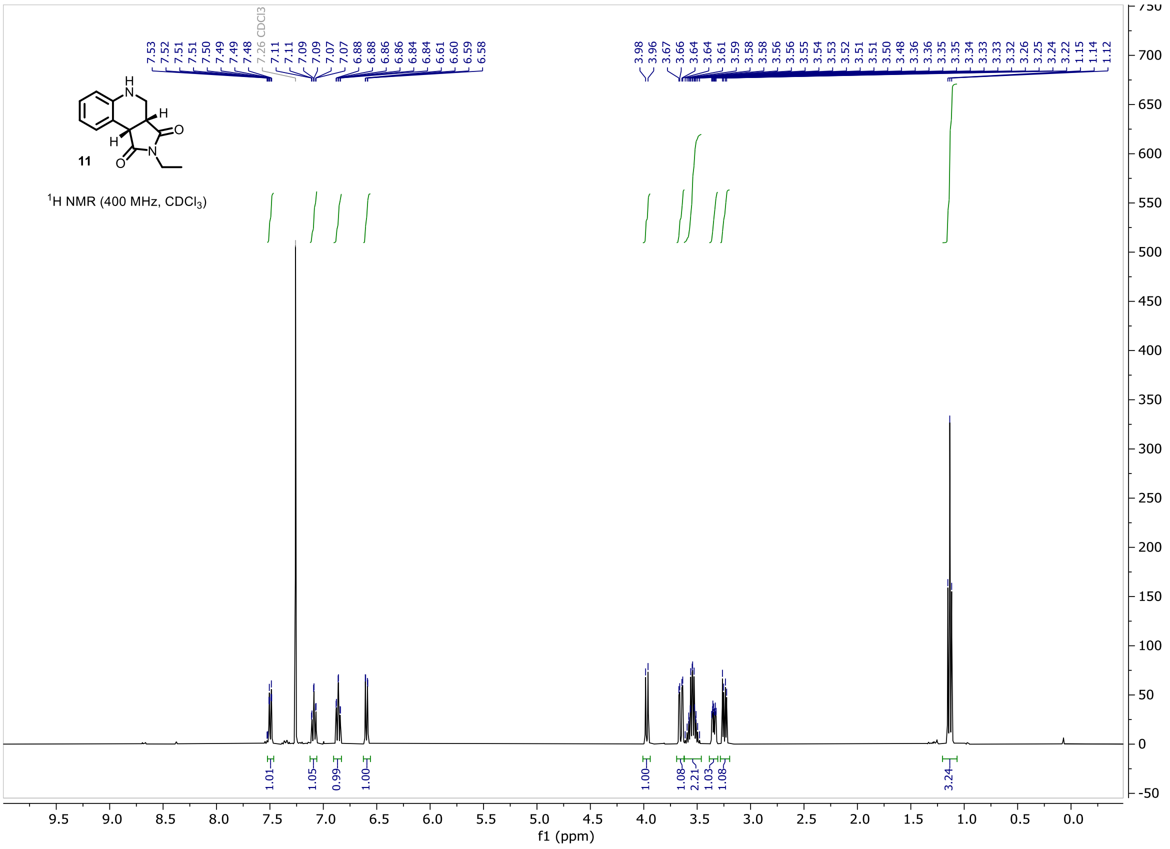
11
^{1}H NMR (400 MHz, CDCl$_3$)
f1 (ppm)

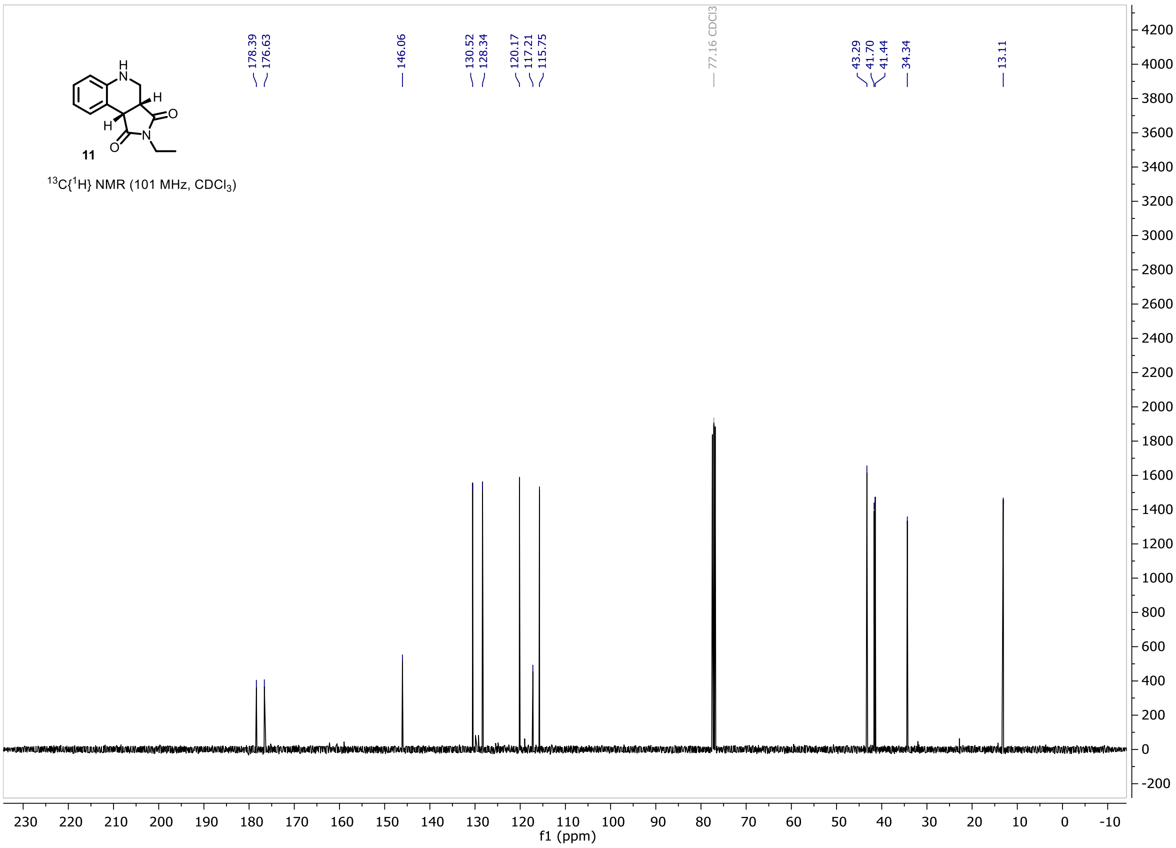
11
^{13}C{^{1}H} NMR (101 MHz, CDCl$_3$)
178.39
176.63
146.06
130.52
128.34
120.17
117.21
115.75
77.16 CDCl3
43.29
41.70
41.44
34.34
13.11
f1 (ppm)

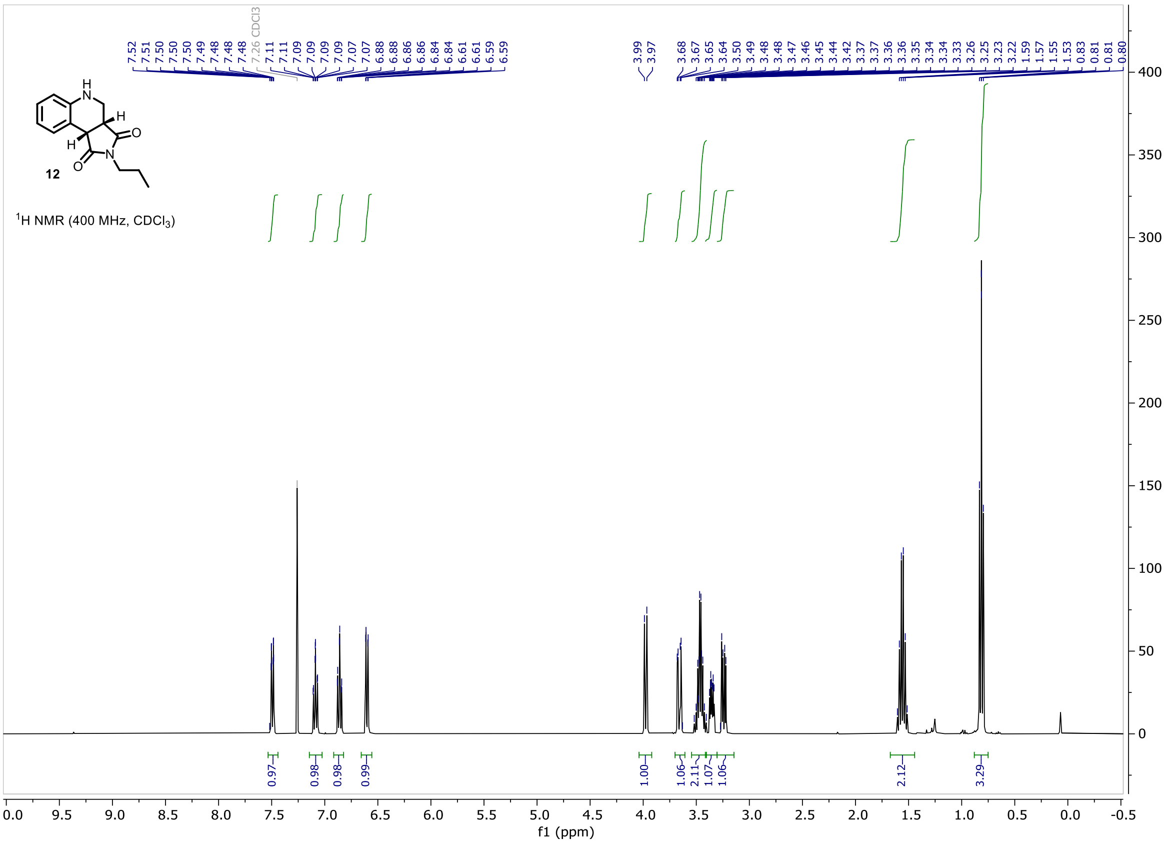
12
1H NMR (400 MHz, CDCl3)
7.52 7.51 7.50 7.50 7.50 7.49 7.48 7.48 7.48 7.26 CDCl3 7.11 7.11 7.09 7.09 7.09 7.09 7.07 7.07 6.88 6.88 6.86 6.86 6.86 6.84 6.61 6.61 6.59 6.59
3.99 3.97 3.68 3.67 3.65 3.64 3.50 3.49 3.48 3.48 3.47 3.46 3.45 3.44 3.42 3.37 3.37 3.36 3.36 3.35 3.34 3.34 3.33 3.26 3.25 3.23 3.22 1.59 1.57 1.55 1.53 0.83 0.81 0.81 0.80
0.97 0.98 0.98 0.99 1.00 1.06 2.11 1.07 1.06 2.12 3.29
f1 (ppm)

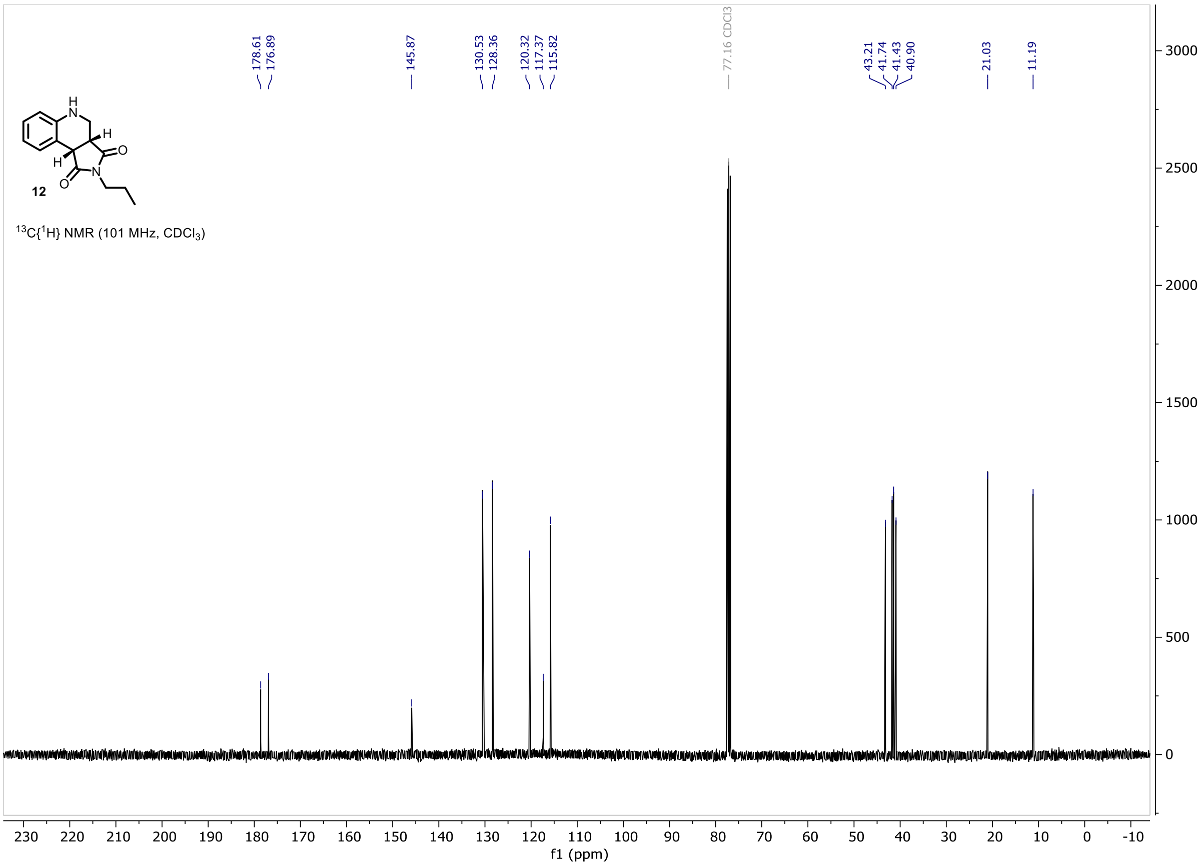
12
13C{1H} NMR (101 MHz, CDCl3)
178.61 176.89 145.87 130.53 128.36 120.32 117.37 115.82 77.16 CDCl3 43.21 41.74 41.43 40.90 21.03 11.19
f1 (ppm)

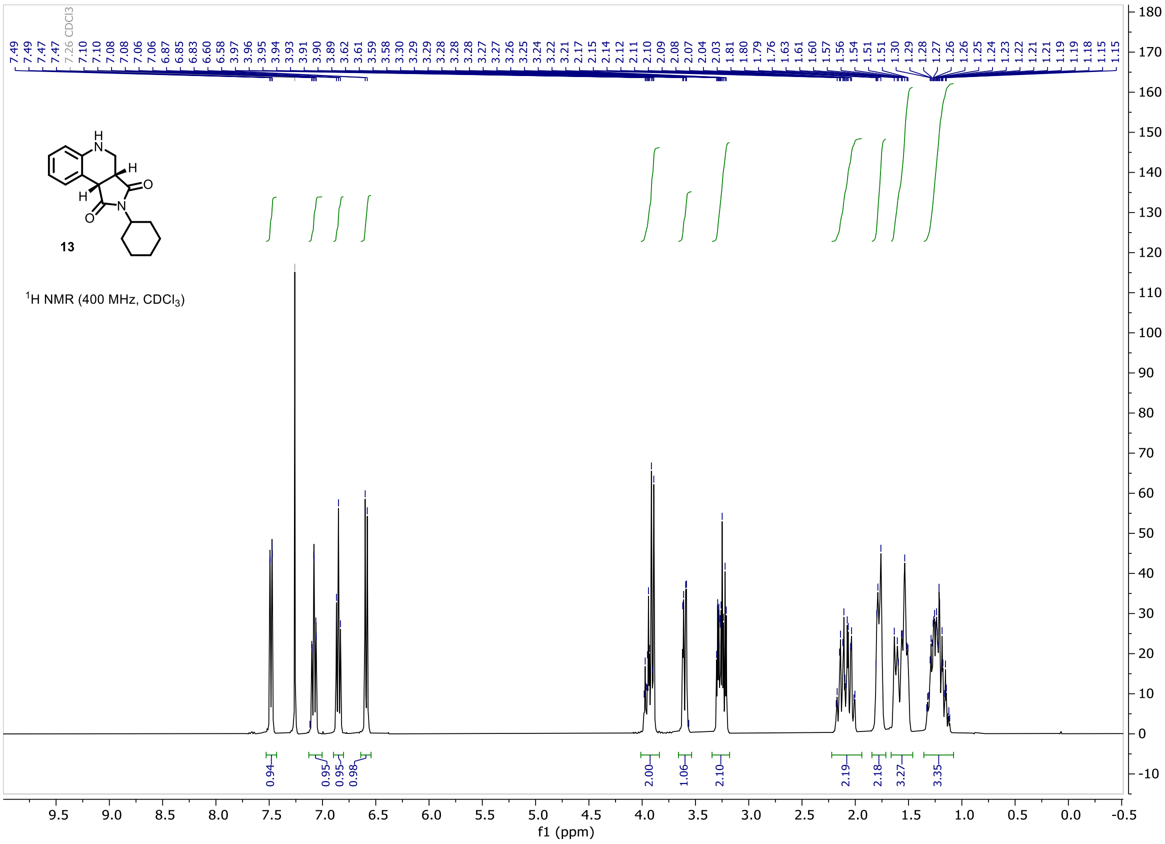
7.49 7.49 7.47 7.26 CDCl3 7.10 7.10 7.08 7.08 7.06 7.06 6.87 6.85 6.83 6.60 6.58 3.97 3.96 3.95 3.94 3.93 3.91 3.90 3.89 3.62 3.61 3.59 3.30 3.29 3.29 3.28 3.28 3.27 3.27 3.26 3.25 3.24 3.22 3.21 2.17 2.15 2.14 2.12 2.11 2.10 2.09 2.08 2.07 2.04 2.03 1.81 1.80 1.79 1.76 1.63 1.61 1.60 1.57 1.56 1.54 1.51 1.51 1.30 1.29 1.28 1.27 1.26 1.26 1.25 1.24 1.23 1.22 1.22 1.21 1.21 1.19 1.19 1.18 1.15 1.15
13
1H NMR (400 MHz, CDCl3)
0.94 0.95 0.95 0.98 2.00 1.06 2.10 2.19 2.18 3.27 3.35
f1 (ppm)

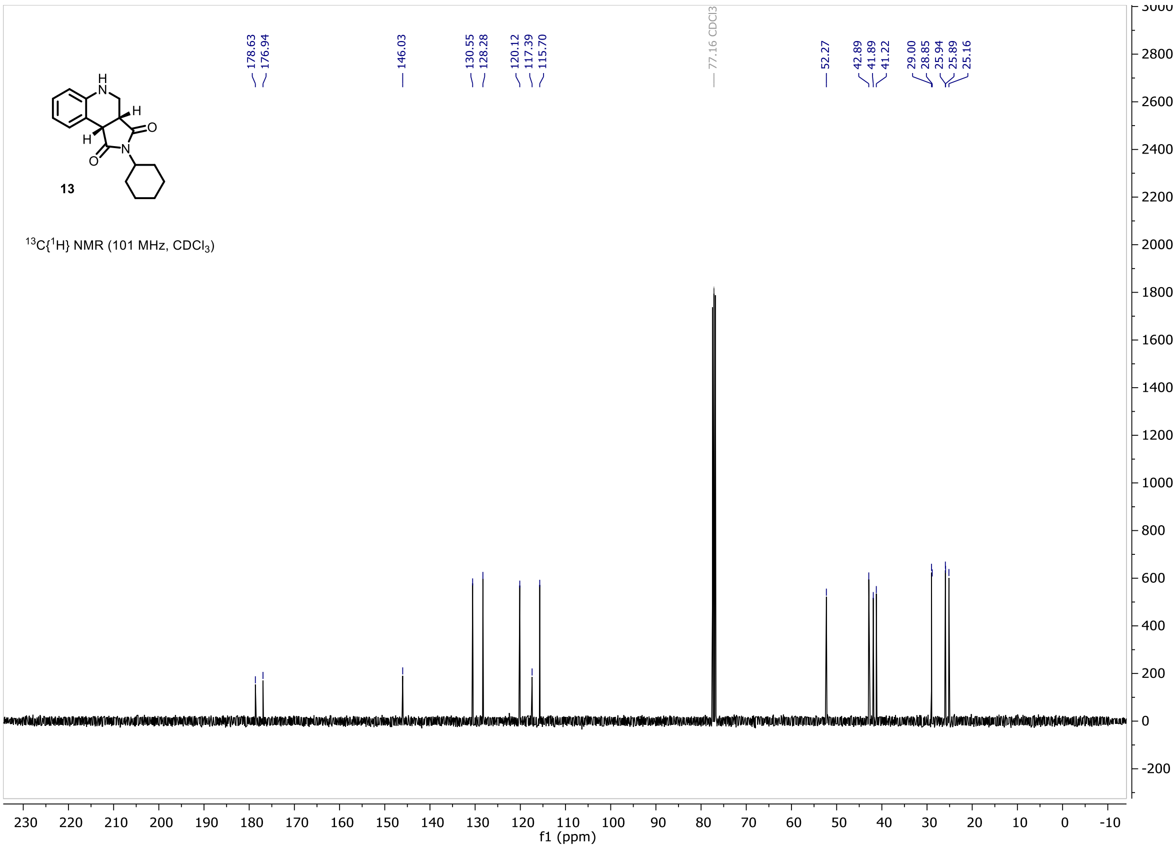
178.63 176.94 146.03 130.55 128.28 120.12 117.39 115.70 77.16 CDCl3 52.27 42.89 41.89 41.22 29.00 28.85 25.94 25.89 25.16
13
13C{1H} NMR (101 MHz, CDCl3)
f1 (ppm)

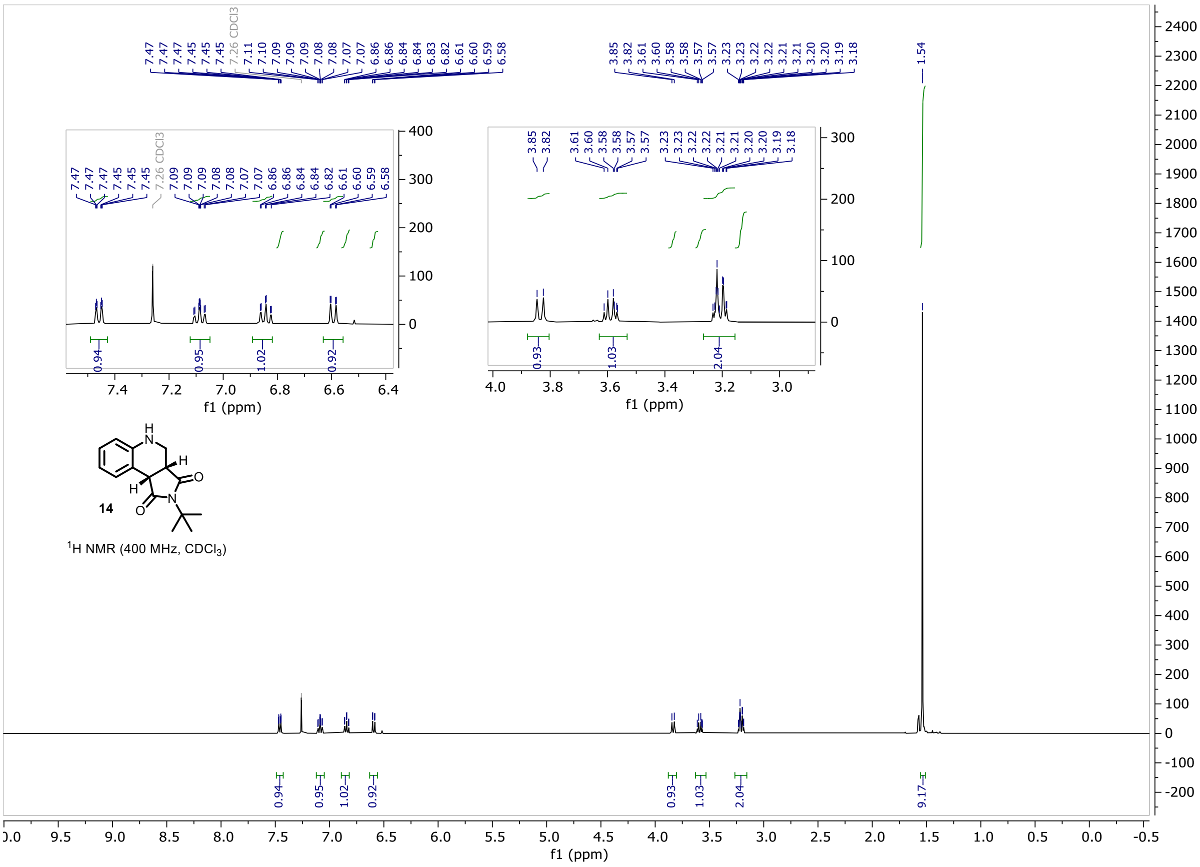
7.47 7.47 7.47 7.45 7.45 7.45 7.26 CDCl3 7.11 7.10 7.09 7.09 7.09 7.08 7.08 7.07 7.07 6.86 6.86 6.84 6.84 6.83 6.82 6.82 6.61 6.60 6.59 6.58
3.85 3.82 3.61 3.60 3.58 3.58 3.57 3.57 3.23 3.23 3.22 3.22 3.21 3.21 3.21 3.20 3.20 3.19 3.18
1.54
0.94
0.95
1.02
0.92
0.93
1.03
2.04
9.17
14
1H NMR (400 MHz, CDCl3)
f1 (ppm)

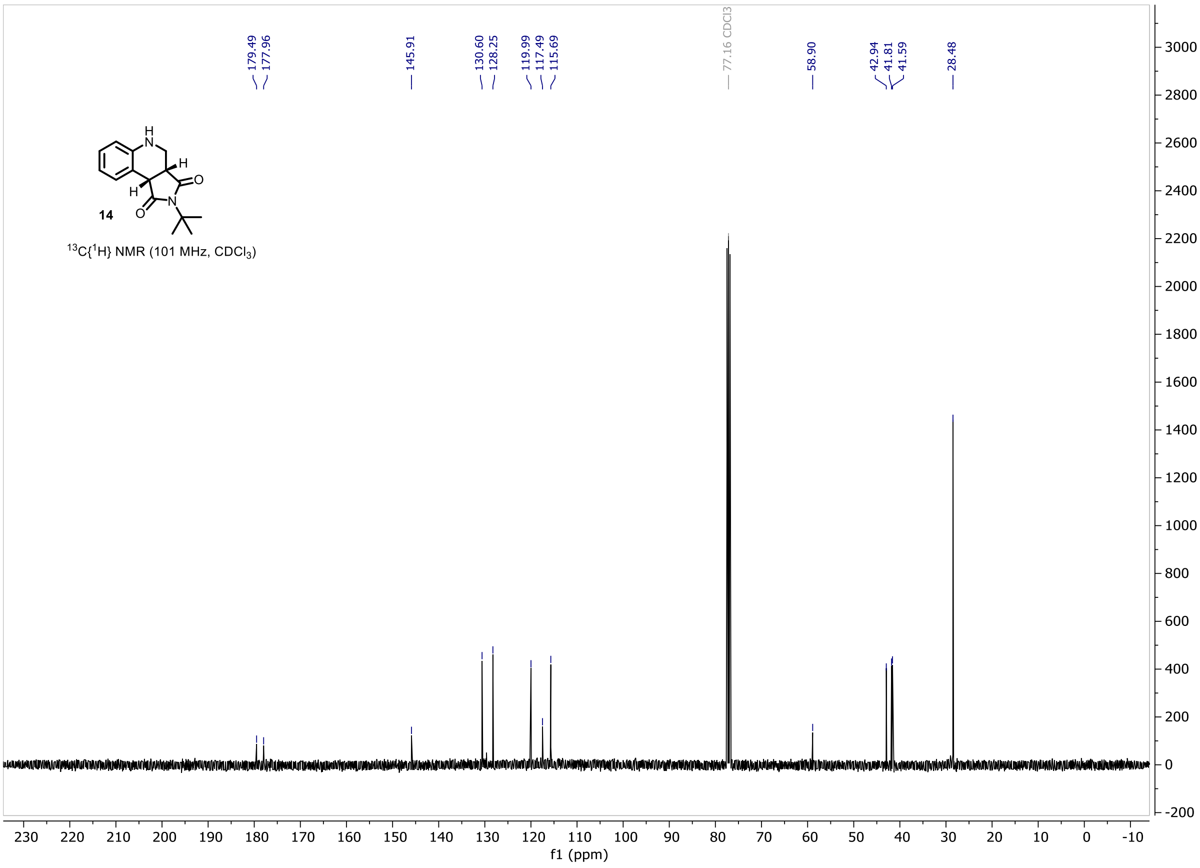
179.49 177.96
145.91
130.60 128.25
119.99 117.49 115.69
77.16 CDCl3
58.90
42.94 41.81 41.59
28.48
14
13C{1H} NMR (101 MHz, CDCl3)
f1 (ppm)

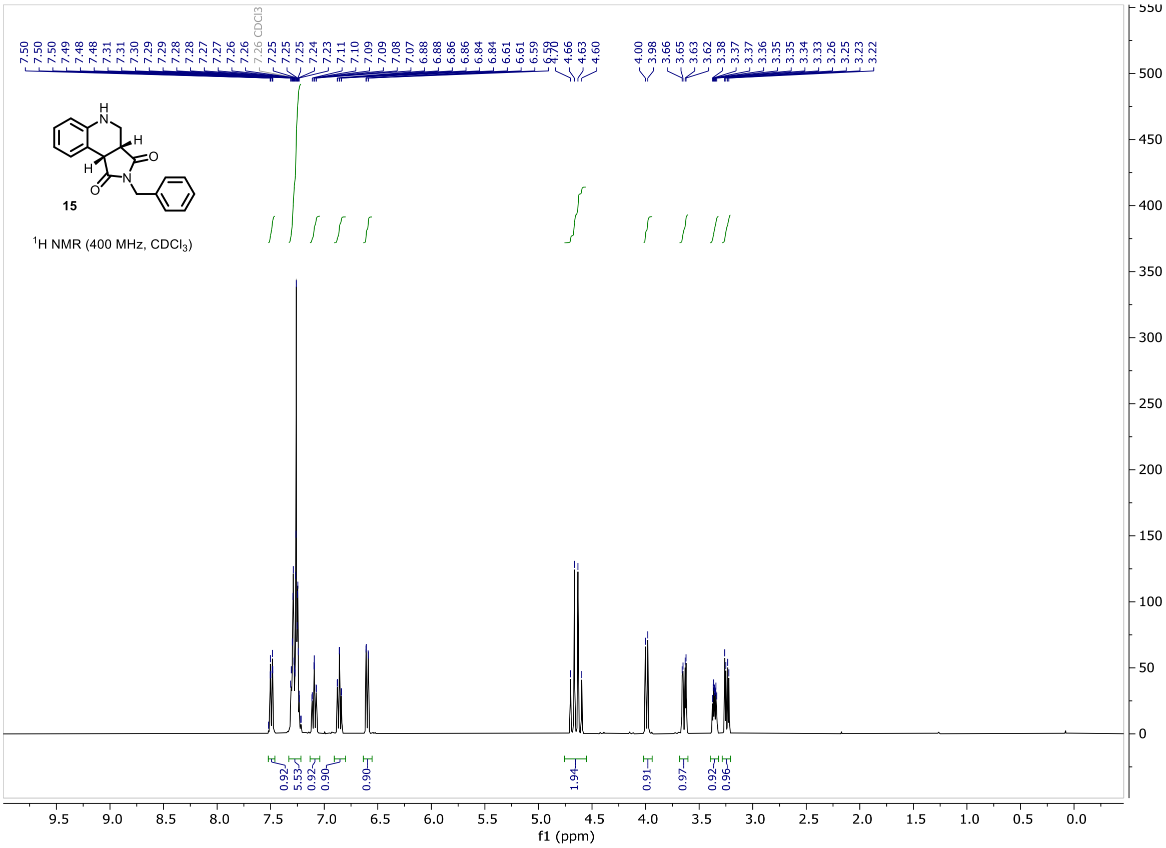
15
^{1}H NMR (400 MHz, CDCl$_3$)
7.50 7.50 7.49 7.48 7.48 7.31 7.31 7.30 7.29 7.29 7.28 7.28 7.27 7.27 7.26 7.26 7.26 CDCl3 7.25 7.25 7.25 7.24 7.23 7.11 7.10 7.09 7.08 7.07 6.88 6.88 6.86 6.86 6.84 6.61 6.61 6.59 6.59 4.70 4.66 4.63 4.60 4.00 3.98 3.66 3.65 3.63 3.62 3.38 3.37 3.37 3.36 3.35 3.35 3.34 3.33 3.26 3.25 3.23 3.22
0.92 5.53 0.92 0.90 0.90 1.94 0.91 0.97 0.92 0.96
f1 (ppm)

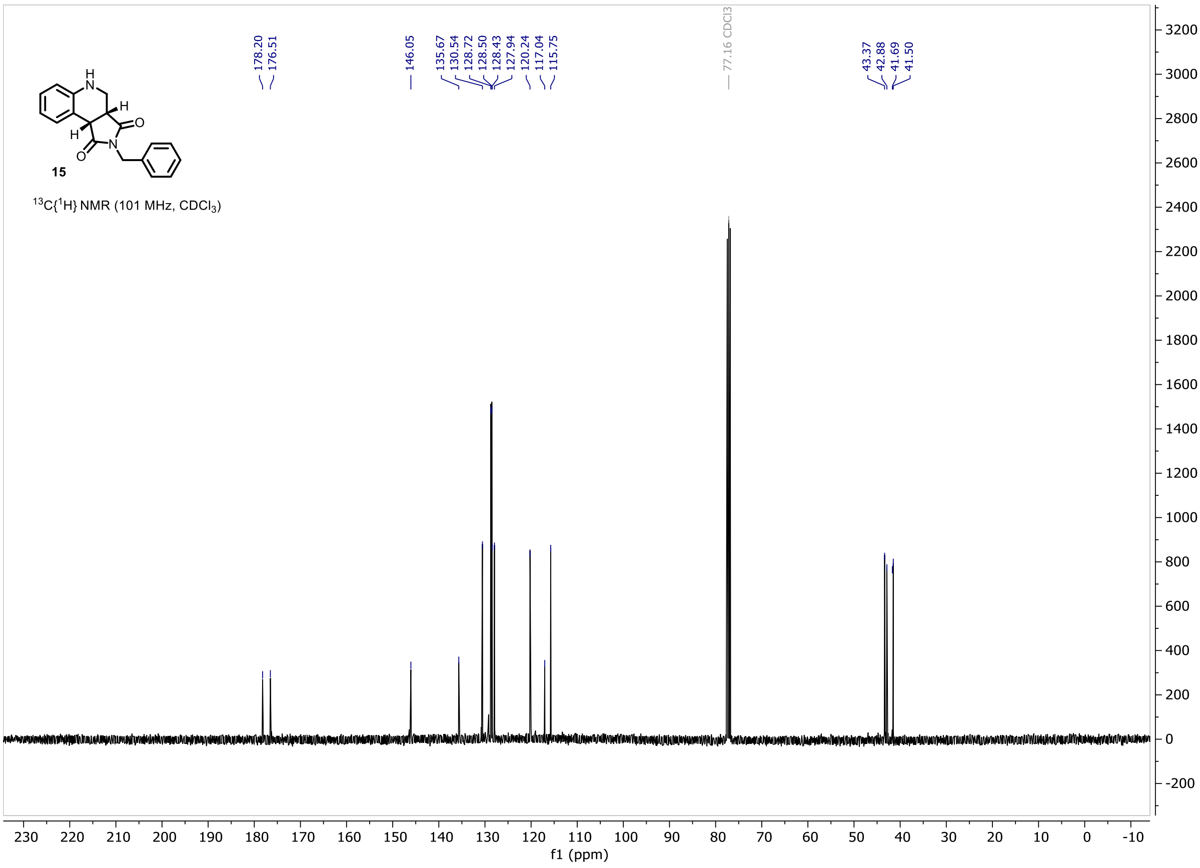
15
^{13}C{^{1}H} NMR (101 MHz, CDCl$_3$)
178.20 176.51 146.05 135.67 130.54 128.72 128.50 128.43 127.94 120.24 117.04 115.75 77.16 CDCl3 43.37 42.88 41.69 41.50
f1 (ppm)

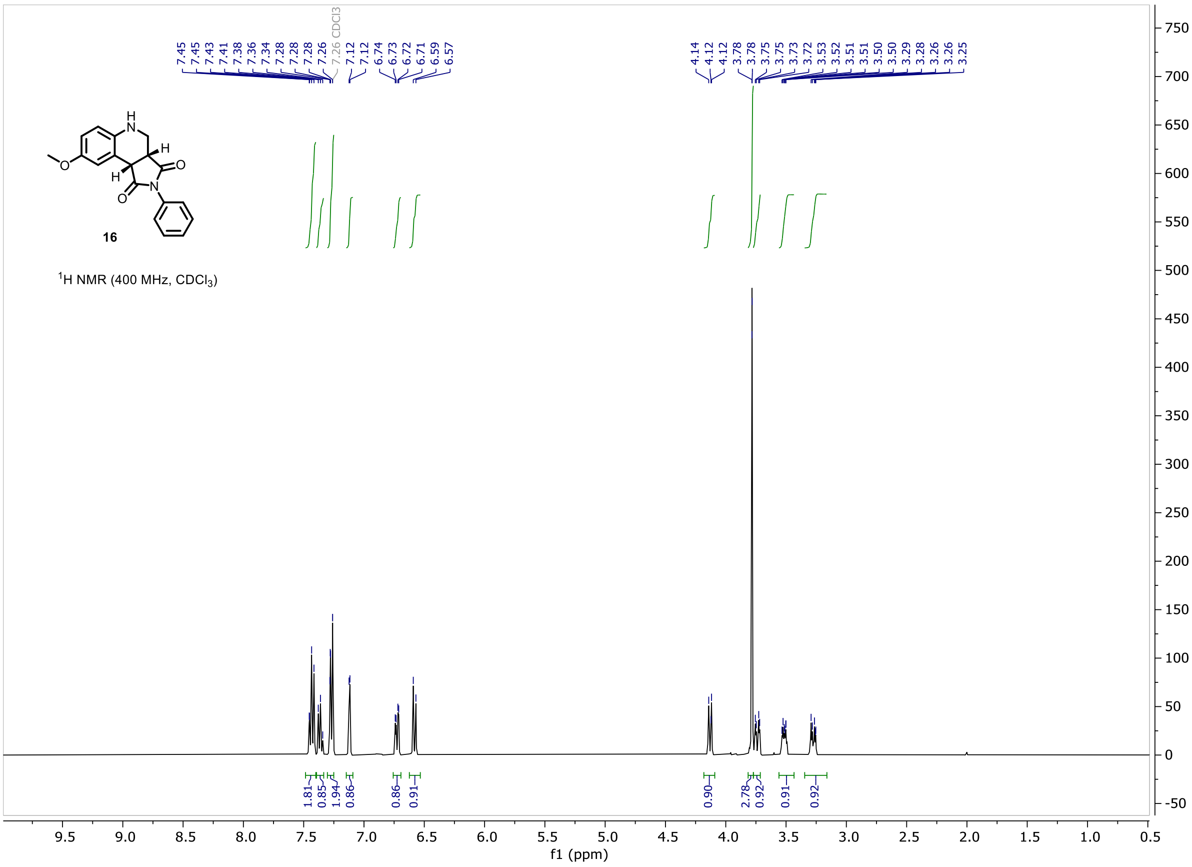
16
^{1}H NMR (400 MHz, CDCl$_3$)
f1 (ppm)

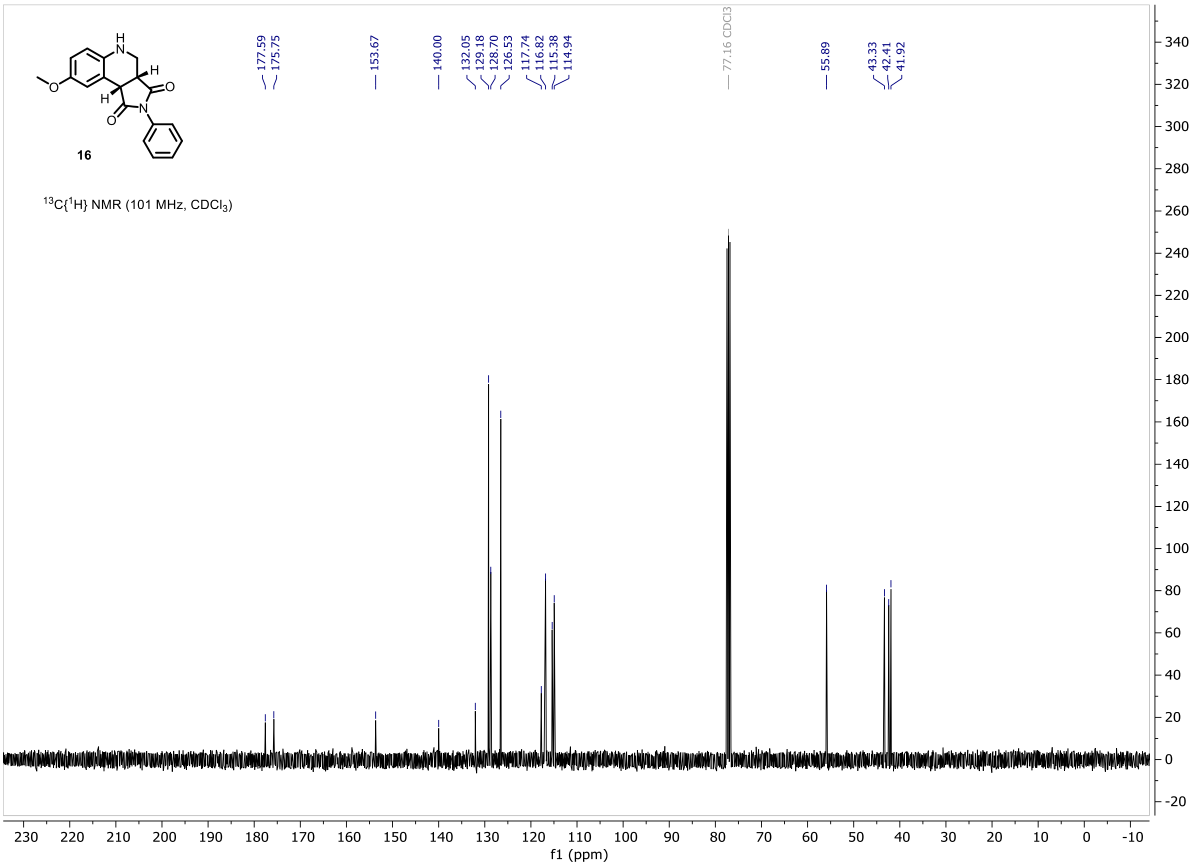
16
^{13}C{^{1}H} NMR (101 MHz, CDCl$_3$)
177.59
175.75
153.67
140.00
132.05
129.18
128.70
126.53
117.74
116.82
115.38
114.94
77.16 CDCl3
55.89
43.33
42.41
41.92
f1 (ppm)

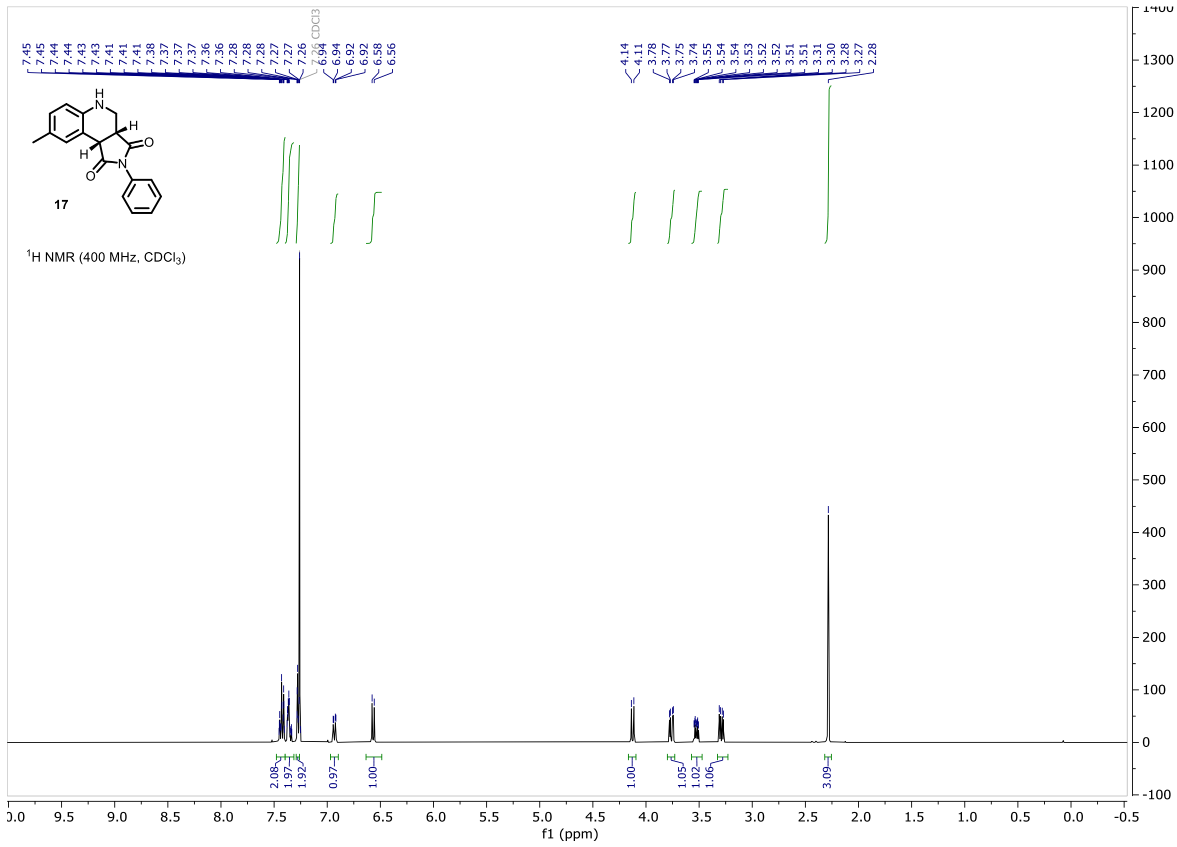
17
1H NMR (400 MHz, CDCl3)
7.45 7.45 7.44 7.44 7.43 7.43 7.41 7.41 7.41 7.38 7.37 7.37 7.37 7.36 7.28 7.28 7.28 7.27 7.27 7.26 7.26 CDCl3 6.94 6.94 6.92 6.92 6.58 6.56
4.14 4.11 3.78 3.77 3.75 3.74 3.55 3.54 3.54 3.53 3.52 3.52 3.51 3.51 3.31 3.30 3.28 3.27 2.28
2.08 1.97 1.92 0.97 1.00 1.00 1.05 1.02 1.06 3.09
f1 (ppm)

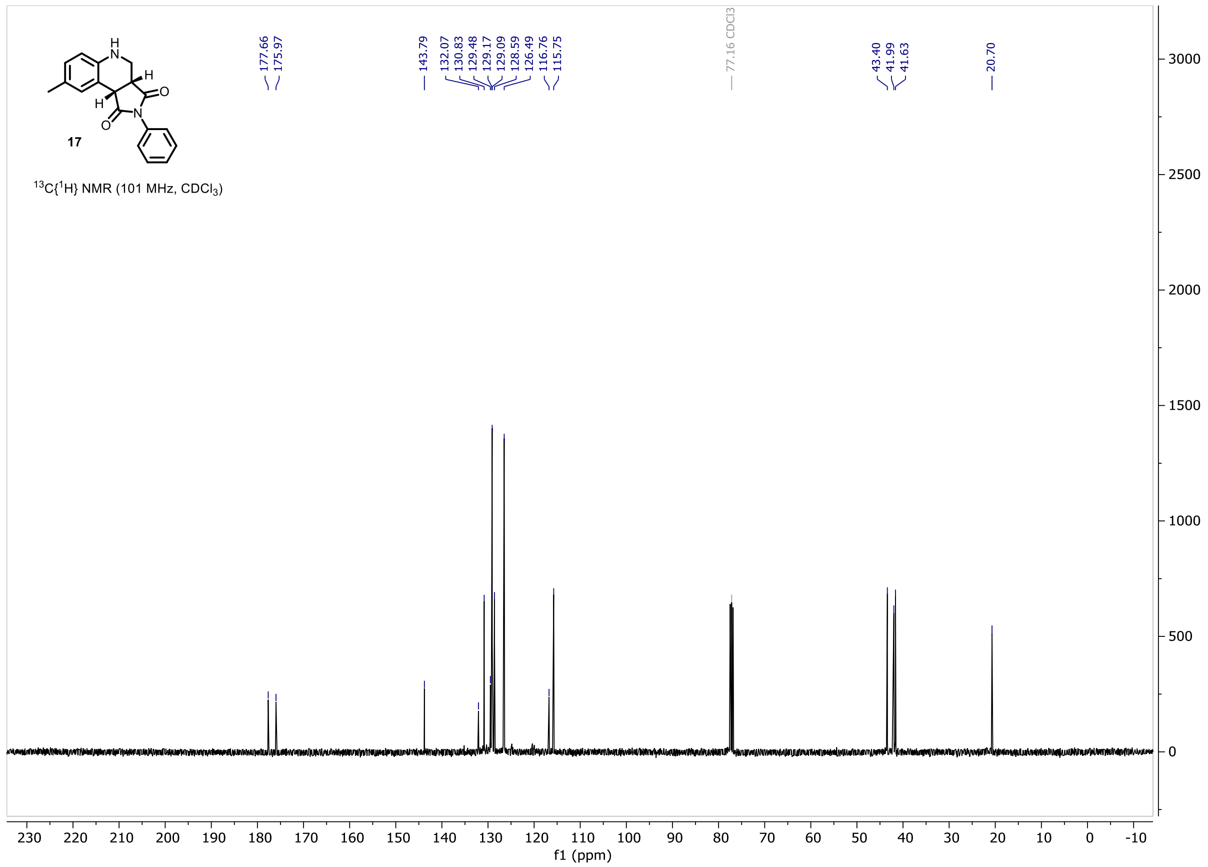
17
13C{1H} NMR (101 MHz, CDCl3)
177.66 175.97 143.79 132.07 130.83 129.48 129.17 129.09 128.59 126.49 116.76 115.75 77.16 CDCl3 43.40 41.99 41.63 20.70
f1 (ppm)

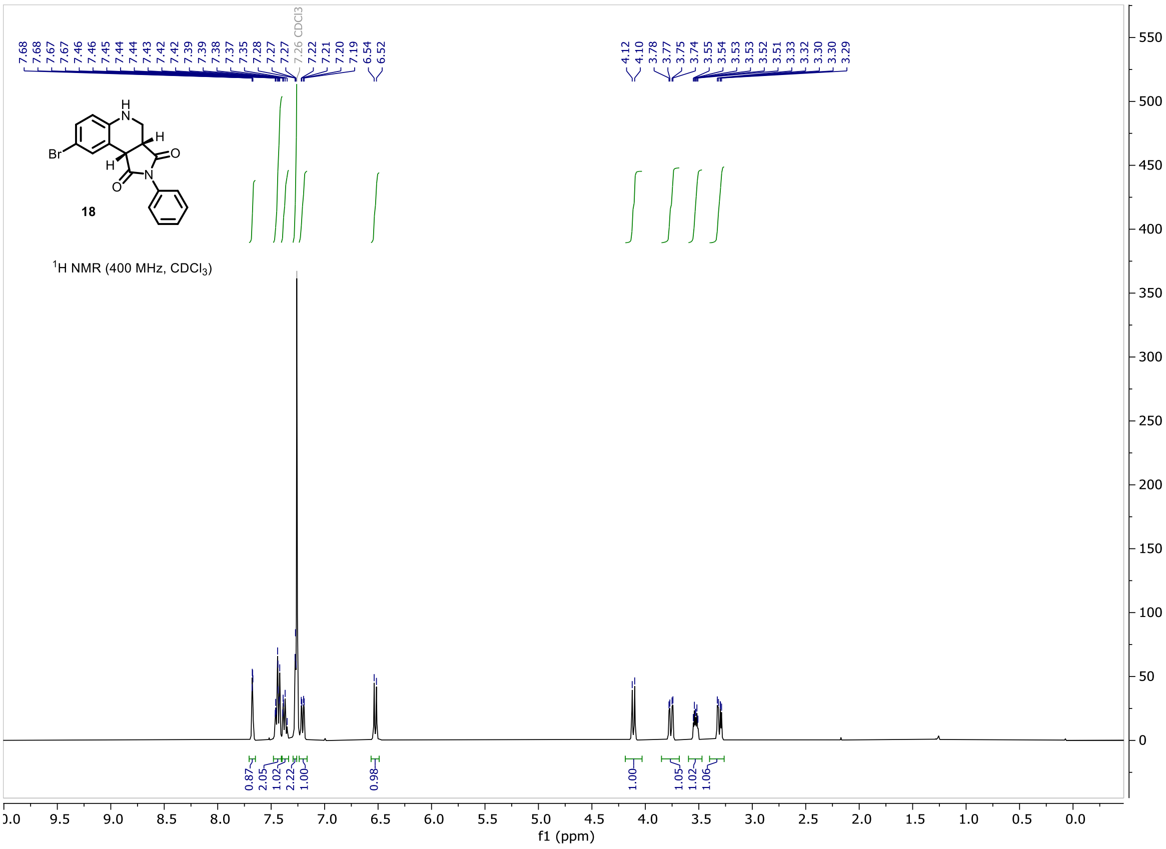
Br
H
N
H
O
O
N
18
¹H NMR (400 MHz, CDCl₃)
f1 (ppm)

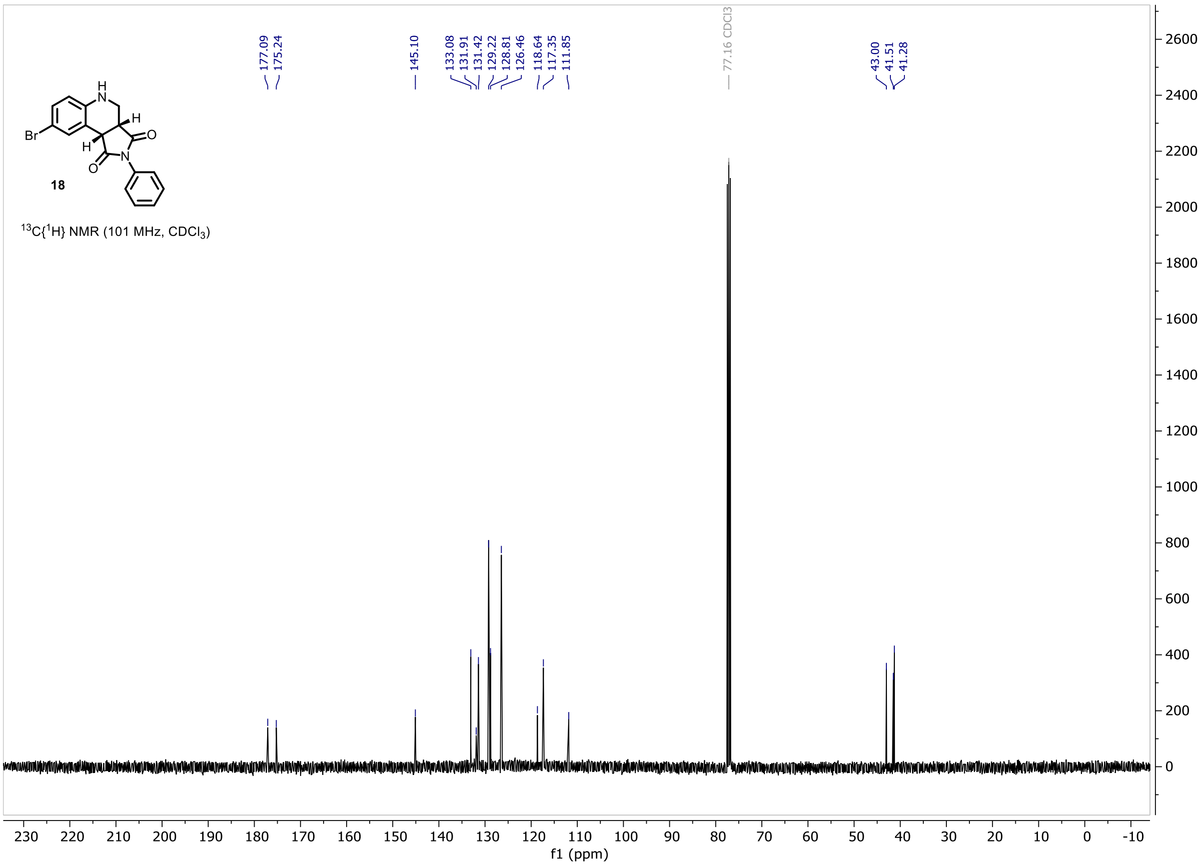
Br
H
N
H
O
O
N
18
¹³C{¹H} NMR (101 MHz, CDCl₃)
f1 (ppm)

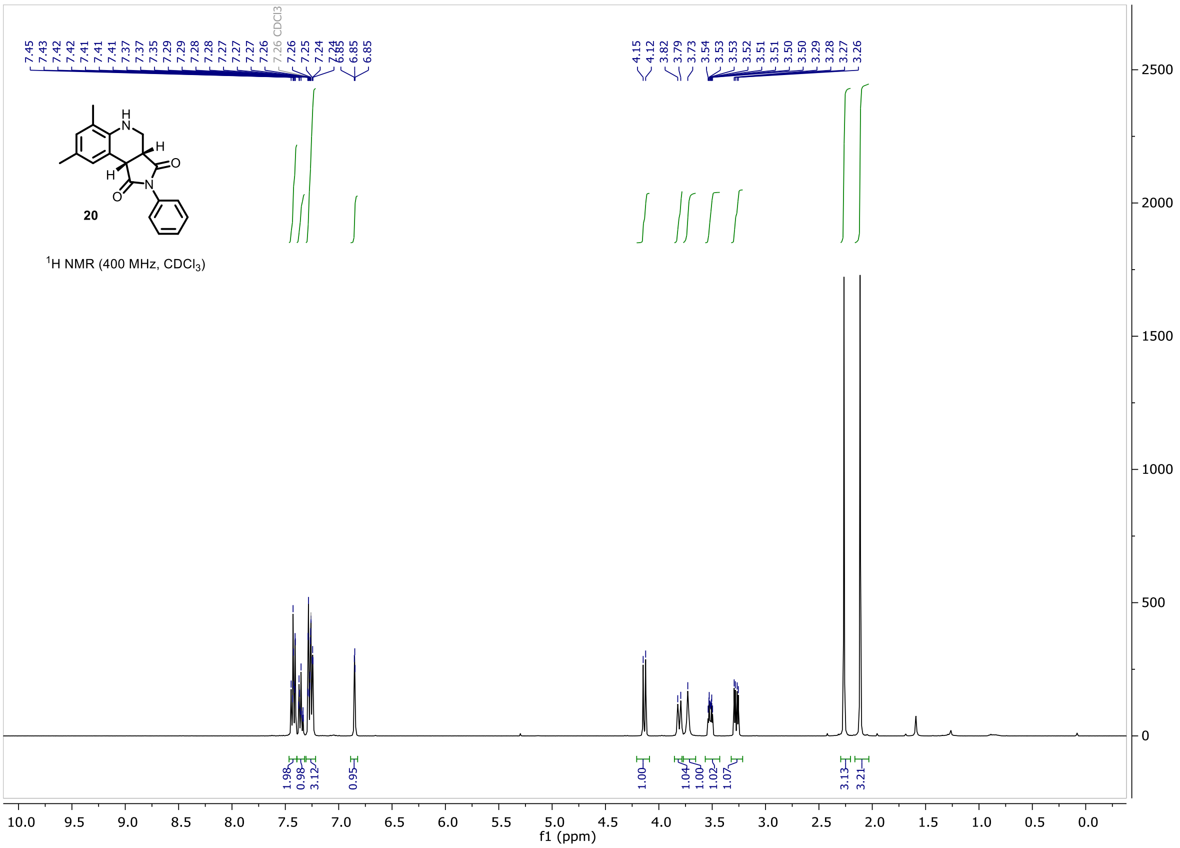

7.45 7.43 7.42 7.42 7.41 7.41 7.41 7.37 7.37 7.35 7.29 7.29 7.28 7.28 7.27 7.27 7.27 7.26
7.26 CDCl3
7.26 7.25 7.24 6.85 6.85 6.85
4.15 4.12 3.82 3.79 3.73 3.54 3.53 3.53 3.52 3.51 3.51 3.50 3.50 3.29 3.28 3.27 3.26
H
N
H
O
H
N
O
20
1H NMR (400 MHz, CDCl3)
1.98 0.98 3.12 0.95 1.00 1.04 1.00 1.02 1.07 3.13 3.21
f1 (ppm)

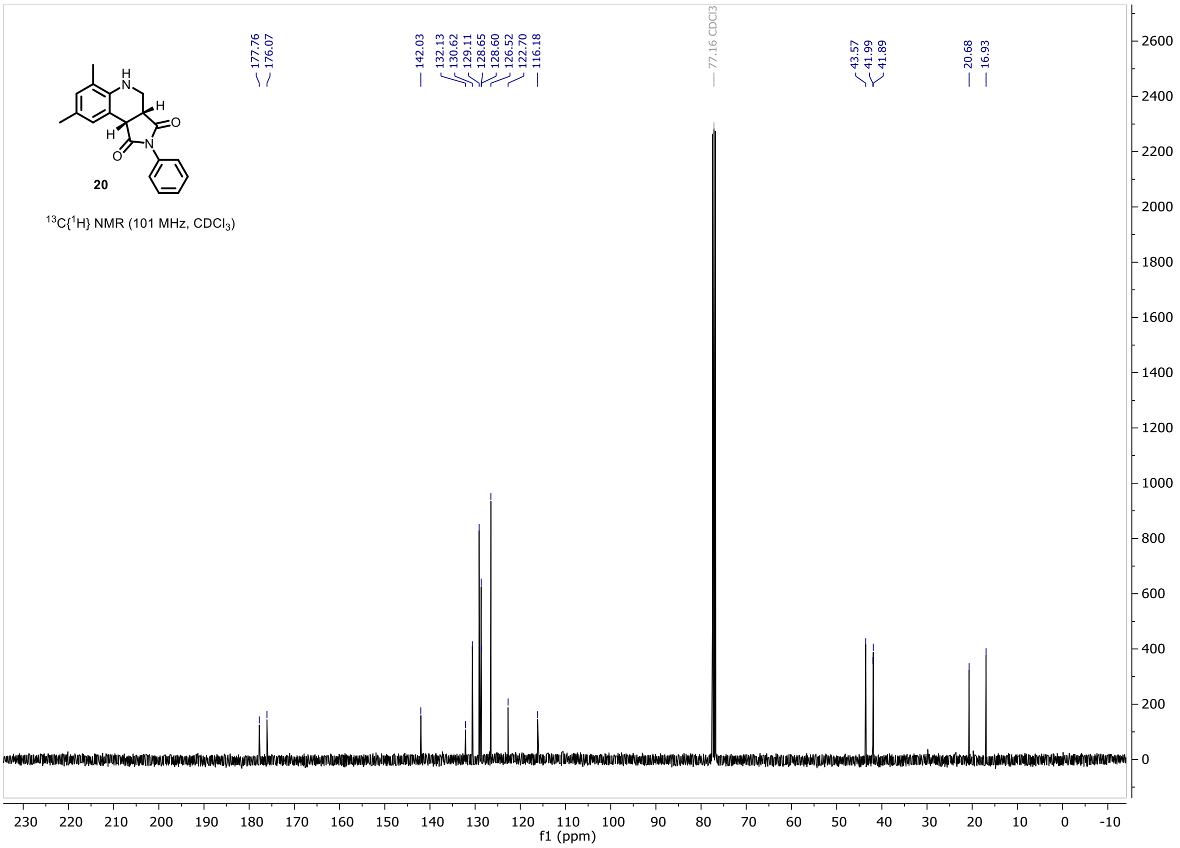

177.76 176.07
142.03 132.13 130.62 129.11 128.65 128.60 126.52 122.70 116.18
77.16 CDCl3
43.57 41.99 41.89
20.68 16.93
H
N
H
O
H
N
O
20
13C{1H} NMR (101 MHz, CDCl3)
f1 (ppm)

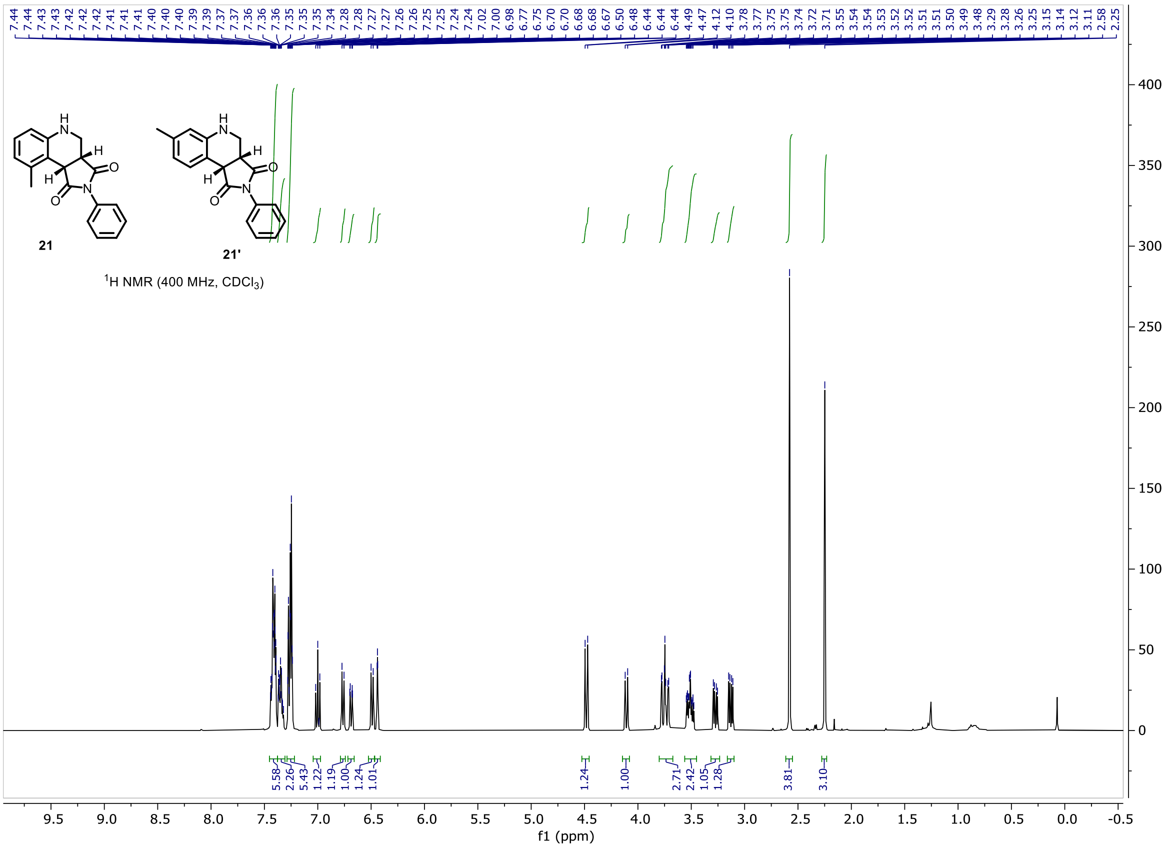
7.44 7.43 7.43 7.42 7.42 7.42 7.41 7.41 7.41 7.40 7.40 7.40 7.39 7.39 7.37 7.37 7.36 7.36 7.35 7.35 7.35 7.34 7.28 7.28 7.27 7.27 7.26 7.26 7.25 7.25 7.24 7.24 7.02 7.00 6.98 6.77 6.75 6.70 6.70 6.68 6.68 6.67 6.50 6.48 6.44 6.44 6.44 4.49 4.47 4.12 4.10 3.78 3.77 3.75 3.75 3.74 3.72 3.71 3.55 3.54 3.54 3.53 3.52 3.52 3.51 3.51 3.50 3.49 3.48 3.29 3.28 3.26 3.25 3.15 3.14 3.12 3.11 2.58 2.25
21
21'
^{1}H NMR (400 MHz, CDCl$_3$)
5.58 2.26 5.43 1.22 1.19 1.00 1.24 1.01 1.24 1.00 2.71 2.42 1.05 1.28 3.81 3.10
f1 (ppm)

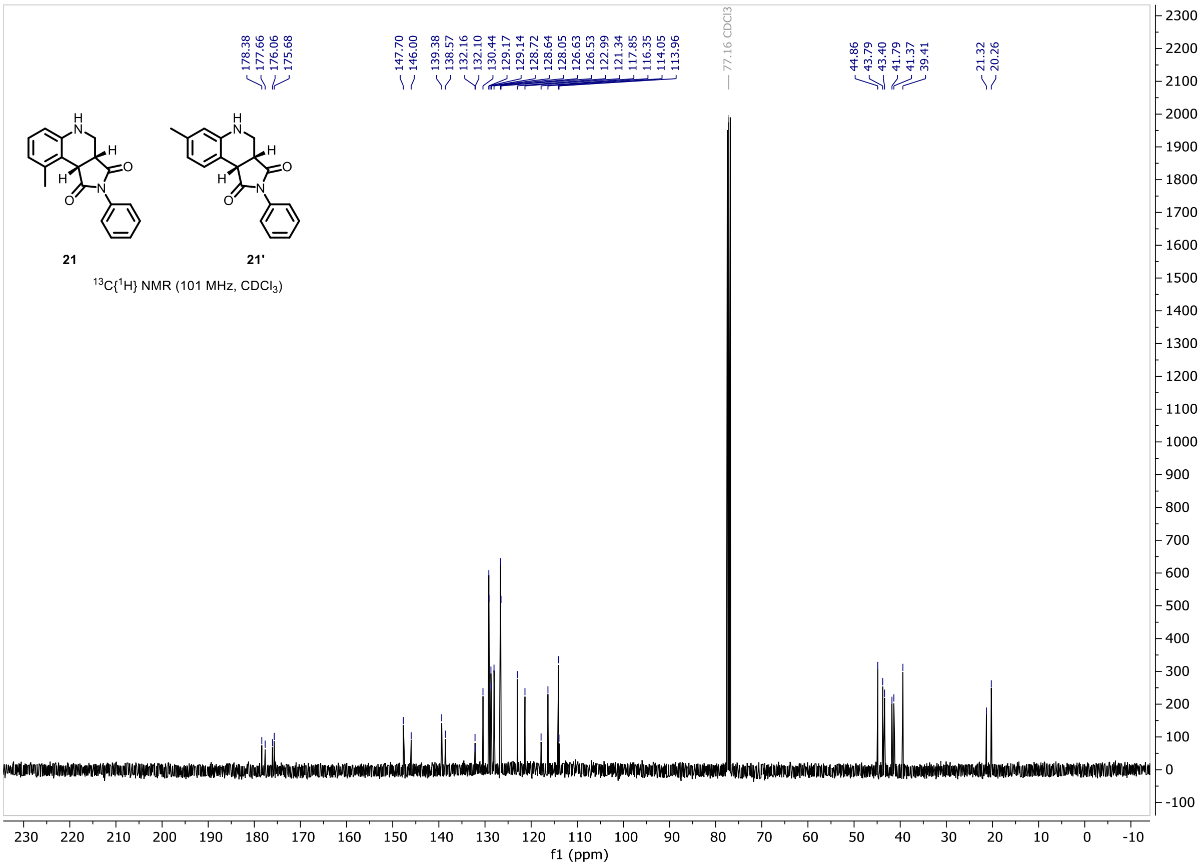
178.38 177.66 176.06 175.68 147.70 146.00 139.38 138.57 132.16 132.10 130.44 129.17 129.14 128.72 128.64 128.05 126.63 126.53 122.99 121.34 117.85 116.35 114.05 113.96
77.16 CDCl3
44.86 43.79 43.40 41.79 41.37 39.41 21.32 20.26
21
21'
^{13}C{^{1}H} NMR (101 MHz, CDCl$_3$)
f1 (ppm)

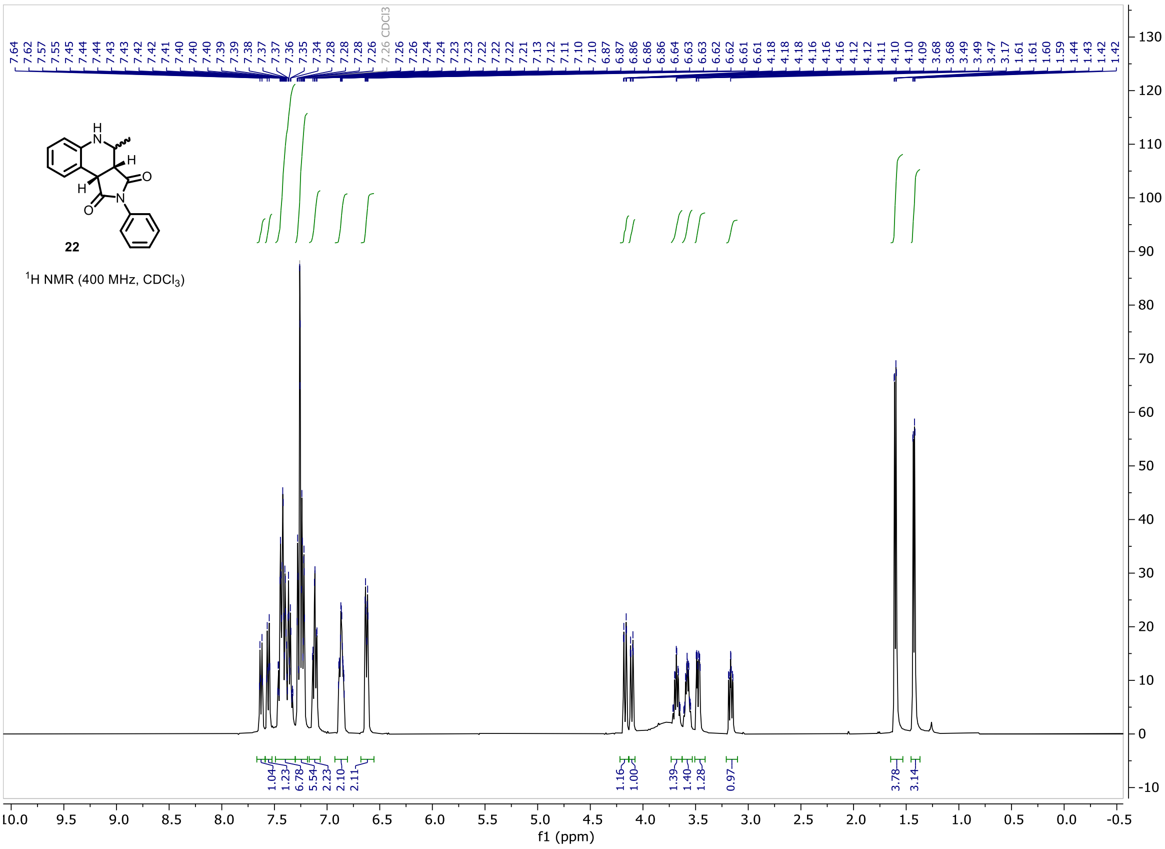
22
^{1}H NMR (400 MHz, CDCl$_3$)
7.64 7.62 7.57 7.55 7.45 7.44 7.44 7.43 7.43 7.42 7.42 7.41 7.40 7.40 7.39 7.39 7.38 7.37 7.37 7.36 7.35 7.34 7.28 7.28 7.26 7.26 CDCl3 7.26 7.26 7.24 7.24 7.23 7.23 7.22 7.22 7.22 7.21 7.13 7.12 7.11 7.10 6.87 6.86 6.86 6.86 6.64 6.63 6.63 6.62 6.62 6.61 6.61 4.18 4.18 4.18 4.16 4.16 4.12 4.12 4.11 4.10 4.10 4.09 3.68 3.49 3.49 3.47 3.17 1.61 1.61 1.60 1.59 1.44 1.43 1.42 1.42
1.04 1.23 6.78 5.54 2.23 2.10 2.11 1.16 1.00 1.39 1.40 1.28 0.97 3.78 3.14
f1 (ppm)

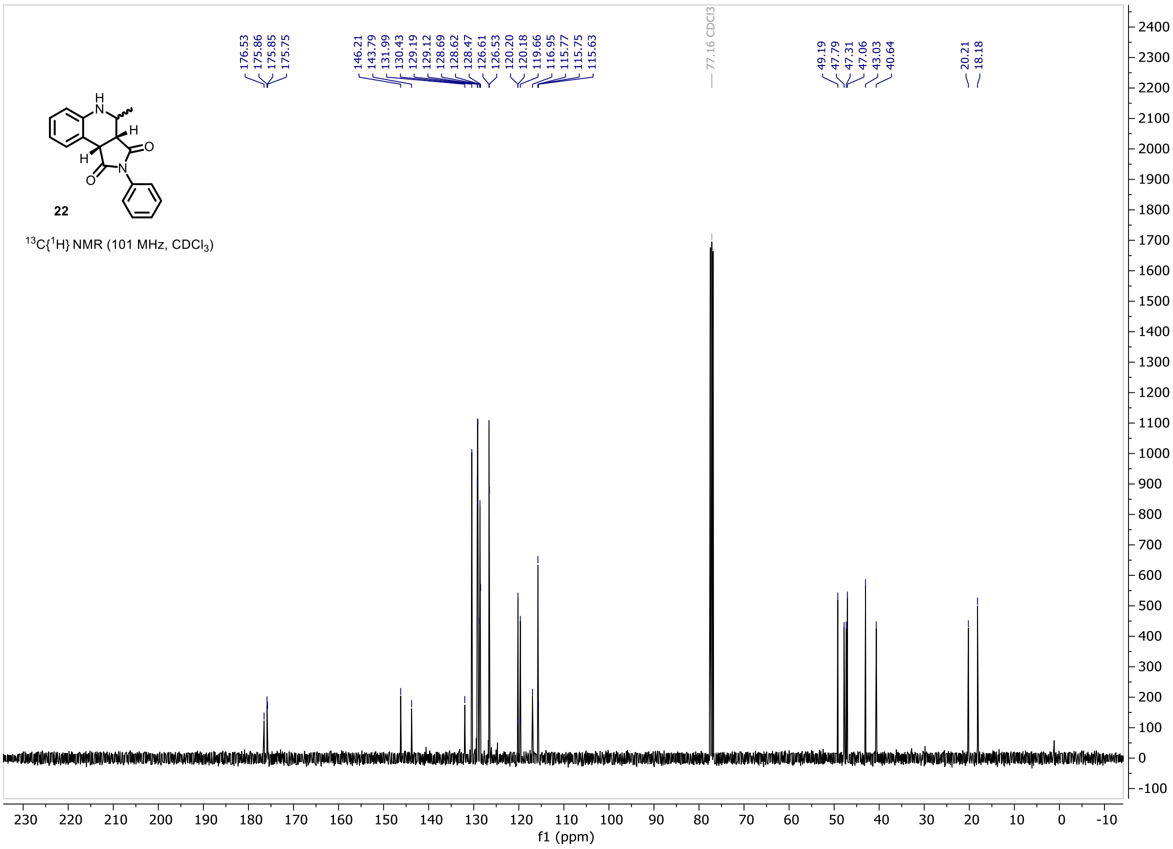
22
^{13}C{^{1}H} NMR (101 MHz, CDCl$_3$)
176.53 175.86 175.85 175.75 146.21 143.79 131.99 130.43 129.19 129.12 128.69 128.62 128.47 126.61 126.53 120.20 120.18 119.66 116.95 115.77 115.75 115.63 77.16 CDCl3 49.19 47.79 47.31 47.06 43.03 40.64 20.21 18.18
f1 (ppm)

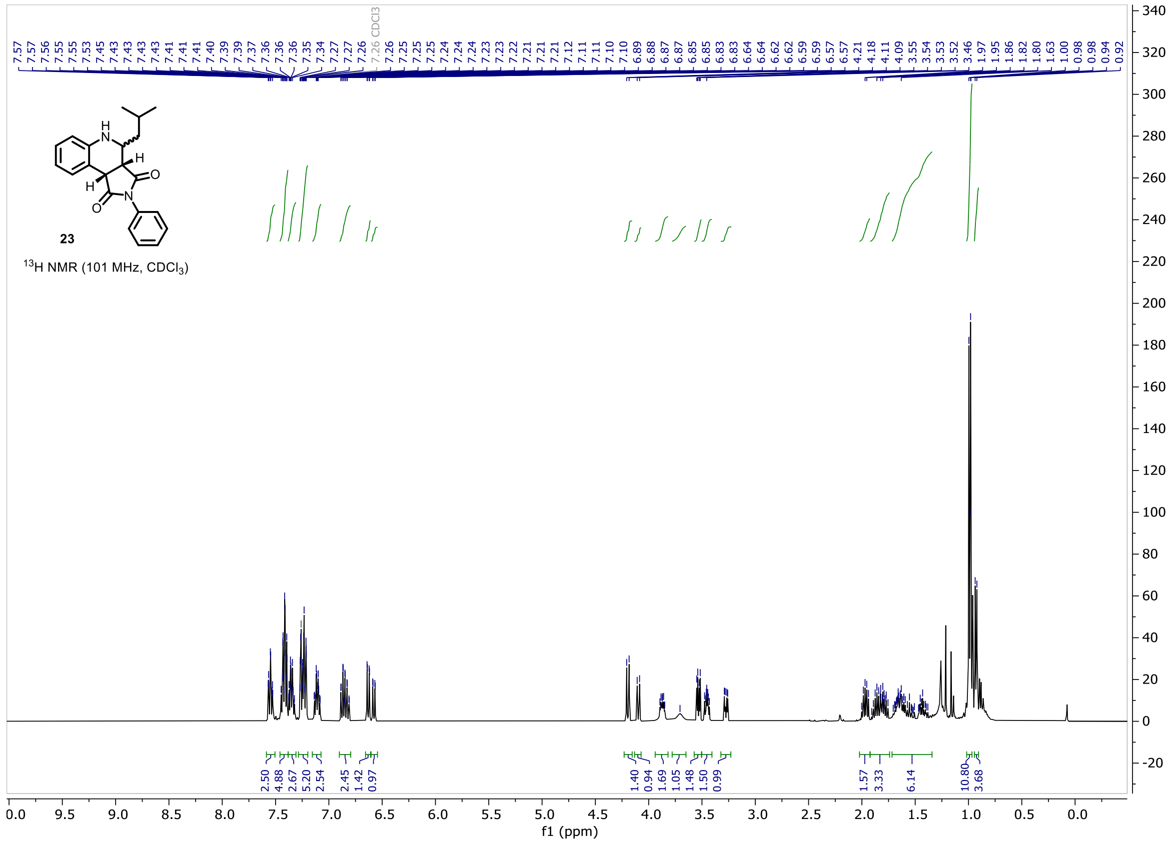
23
13H NMR (101 MHz, CDCl3)
f1 (ppm)

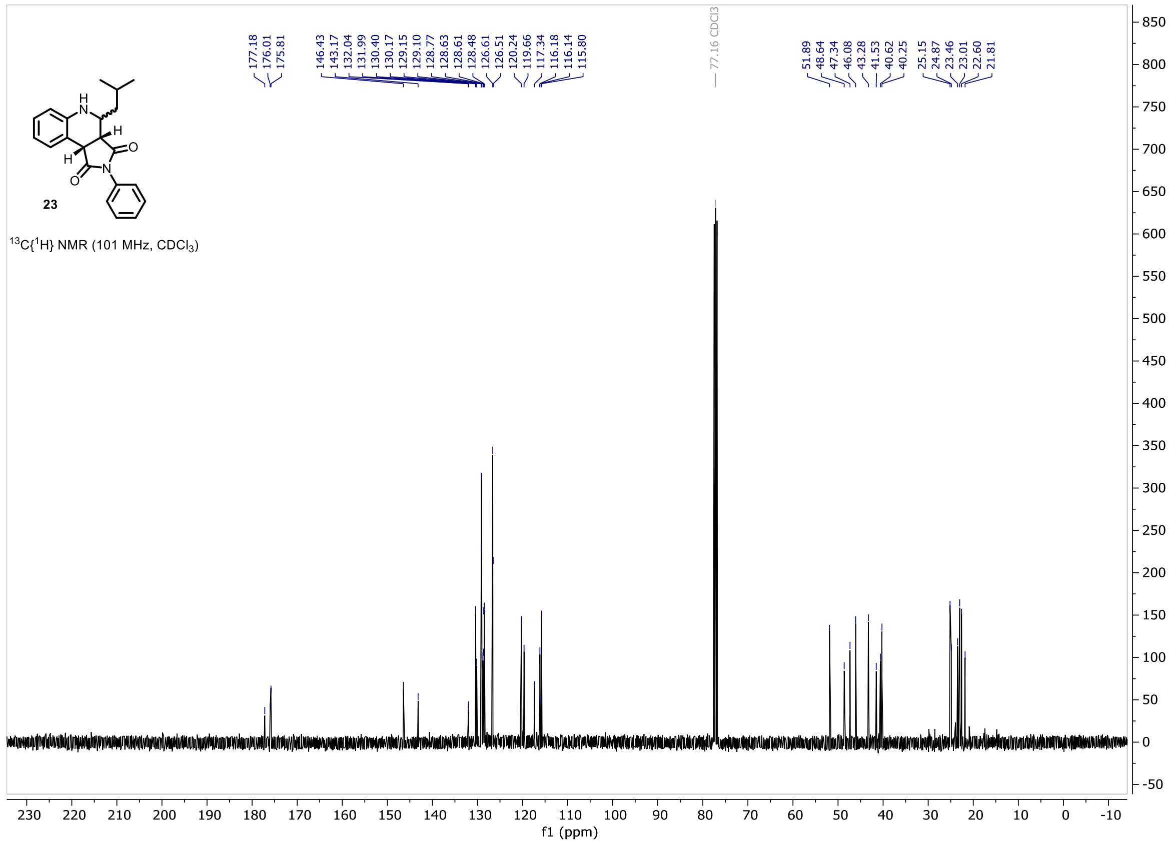
23
13C{1H} NMR (101 MHz, CDCl3)
f1 (ppm)

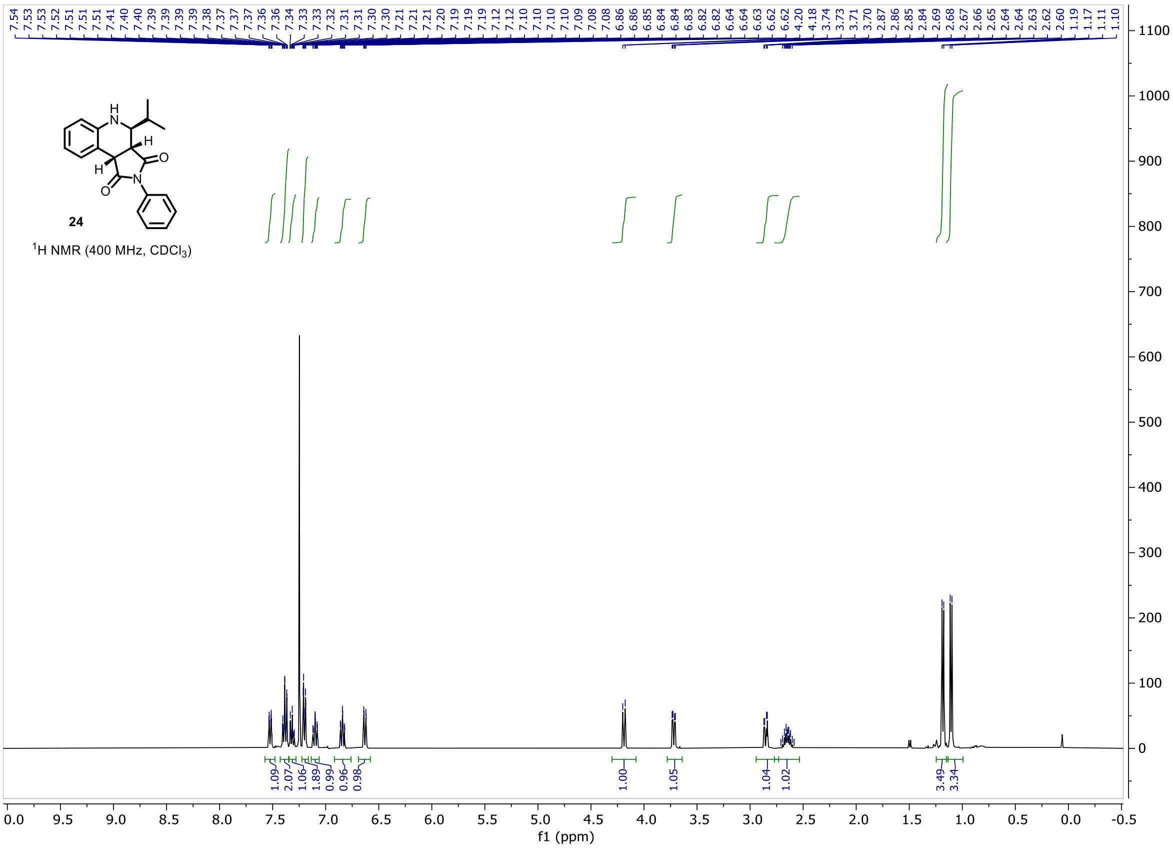
7.54 7.53 7.53 7.52 7.51 7.51 7.41 7.40 7.39 7.39 7.39 7.39 7.38 7.37 7.37 7.36 7.34 7.33 7.33 7.32 7.31 7.31 7.30 7.21 7.21 7.21 7.20 7.19 7.19 7.19 7.12 7.12 7.10 7.10 7.10 7.10 7.09 7.08 7.08 6.86 6.86 6.85 6.84 6.84 6.83 6.82 6.82 6.64 6.64 6.63 6.62 6.62 4.20 4.18 3.74 3.73 3.71 3.70 2.87 2.86 2.85 2.84 2.69 2.68 2.67 2.66 2.65 2.64 2.64 2.63 2.62 2.60 1.19 1.17 1.11 1.10
24
^{1}H NMR (400 MHz, $CDCl_3$)
1.09 2.07 1.06 1.89 0.99 0.96 0.98 1.00 1.05 1.04 1.02 3.49 3.34
f1 (ppm)

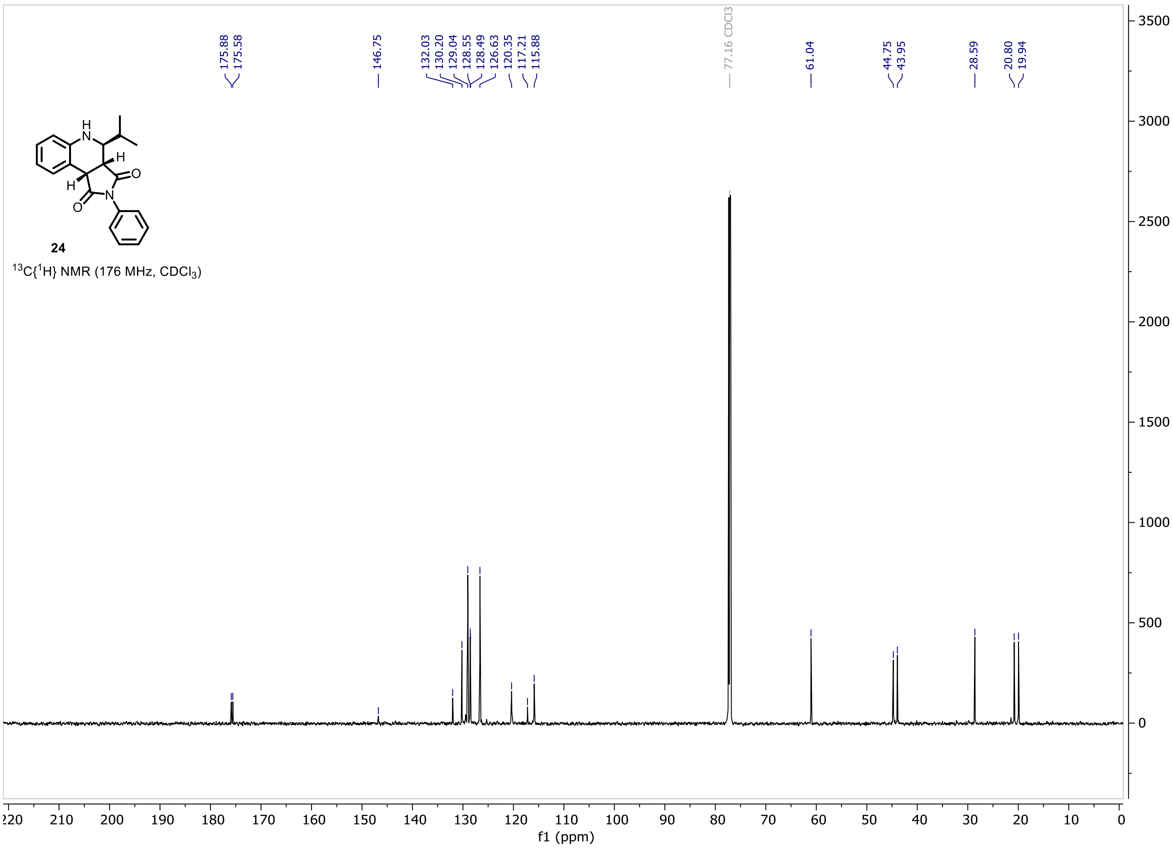
175.88 175.58 146.75 132.03 130.20 129.04 128.55 128.49 126.63 120.35 117.21 115.88 77.16 CDCl3 61.04 44.75 43.95 28.59 20.80 19.94
24
$^{13}C\{^{1}H\}$ NMR (176 MHz, $CDCl_3$)
f1 (ppm)

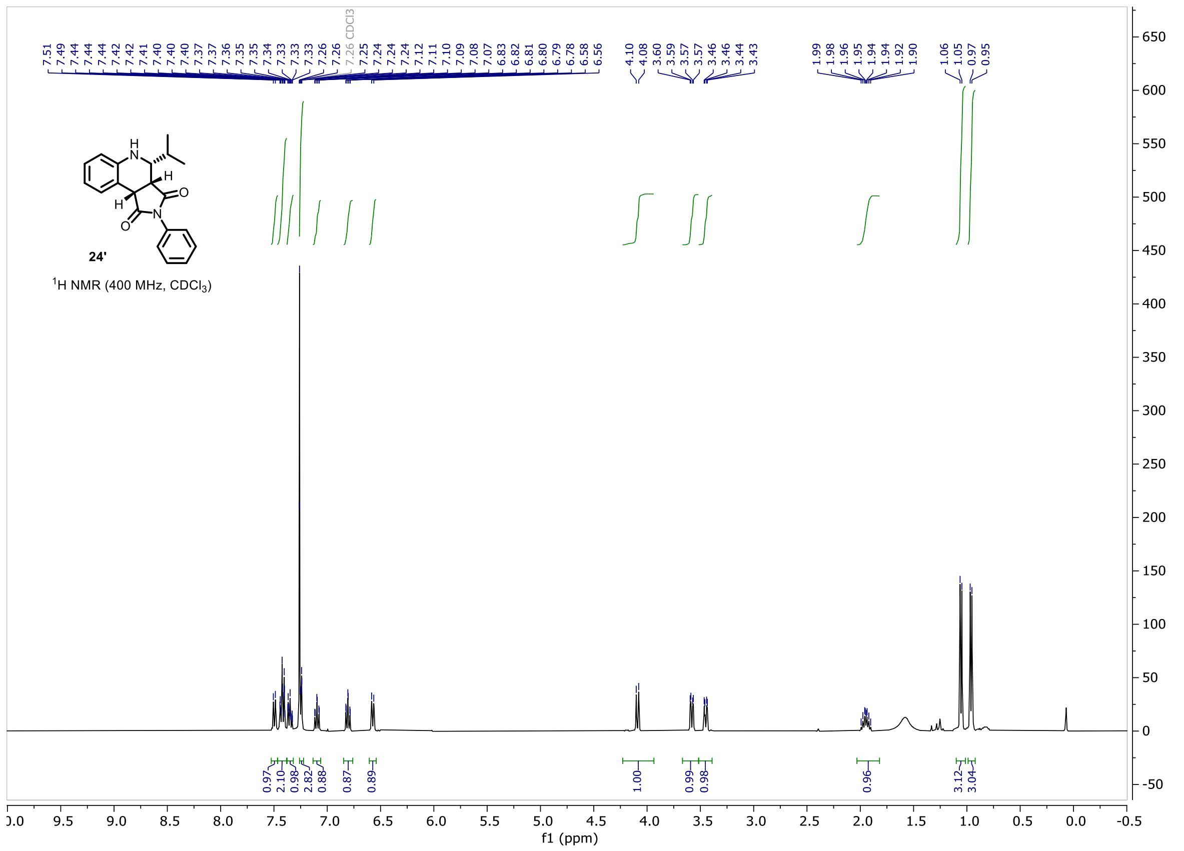
24'
^{1}H NMR (400 MHz, CDCl$_3$)
f1 (ppm)

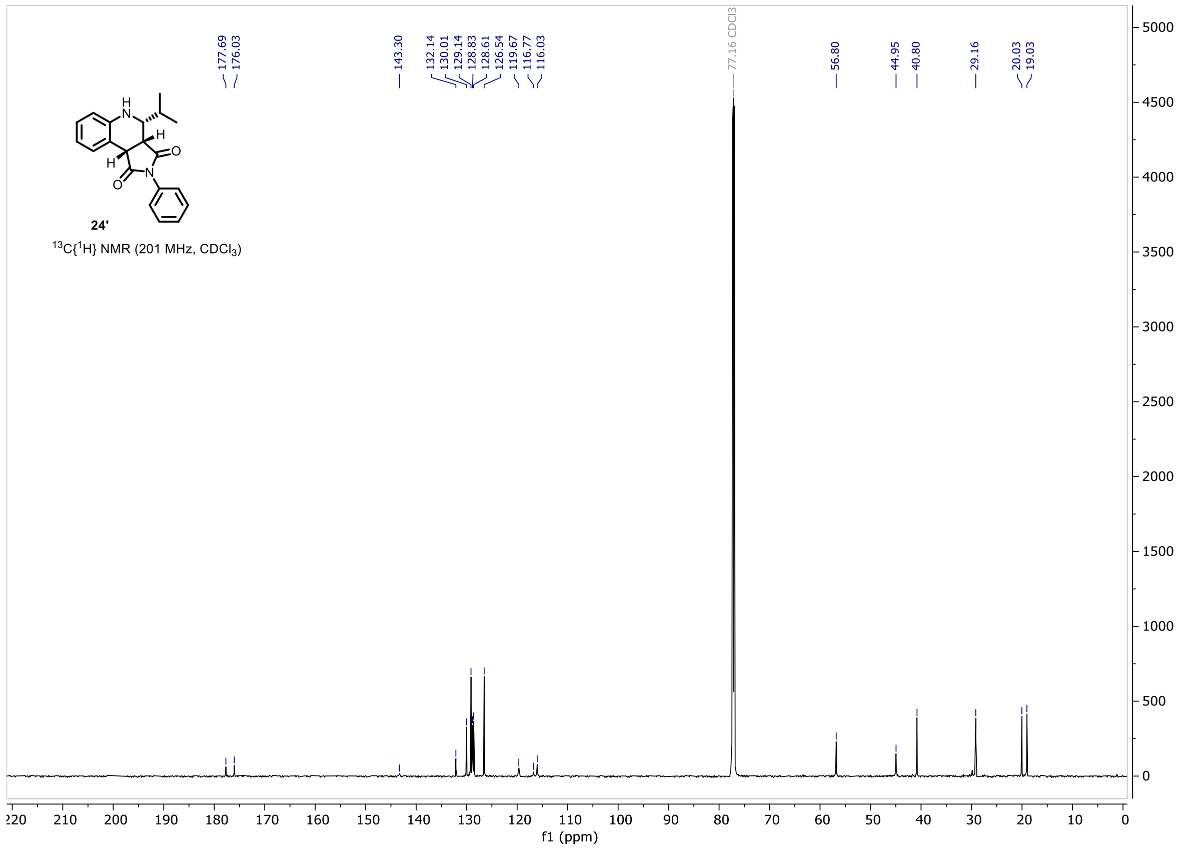
24'
^{13}C{^{1}H} NMR (201 MHz, CDCl$_3$)
177.69
176.03
143.30
132.14
130.01
129.14
128.83
128.61
126.54
119.67
116.77
116.03
77.16 CDCl3
56.80
44.95
40.80
29.16
20.03
19.03
f1 (ppm)

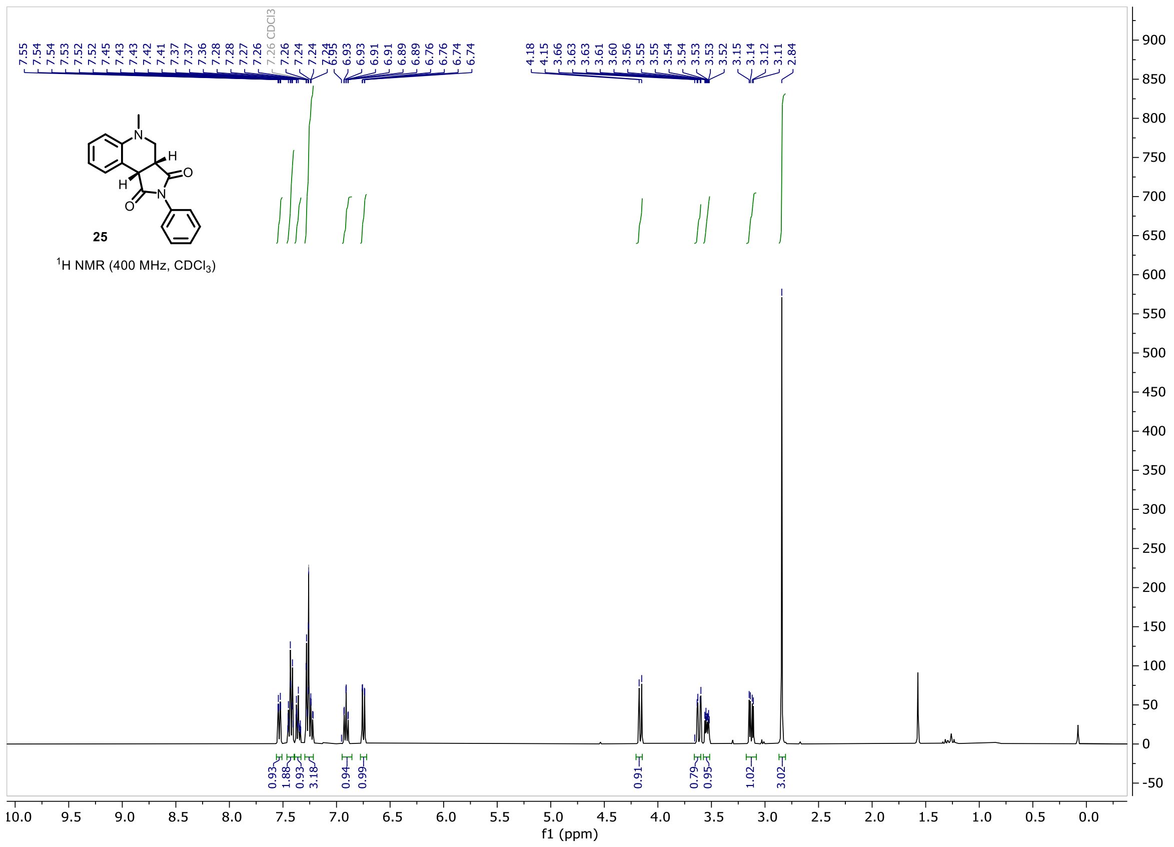

25
^{1}H NMR (400 MHz, CDCl$_3$)
f1 (ppm)

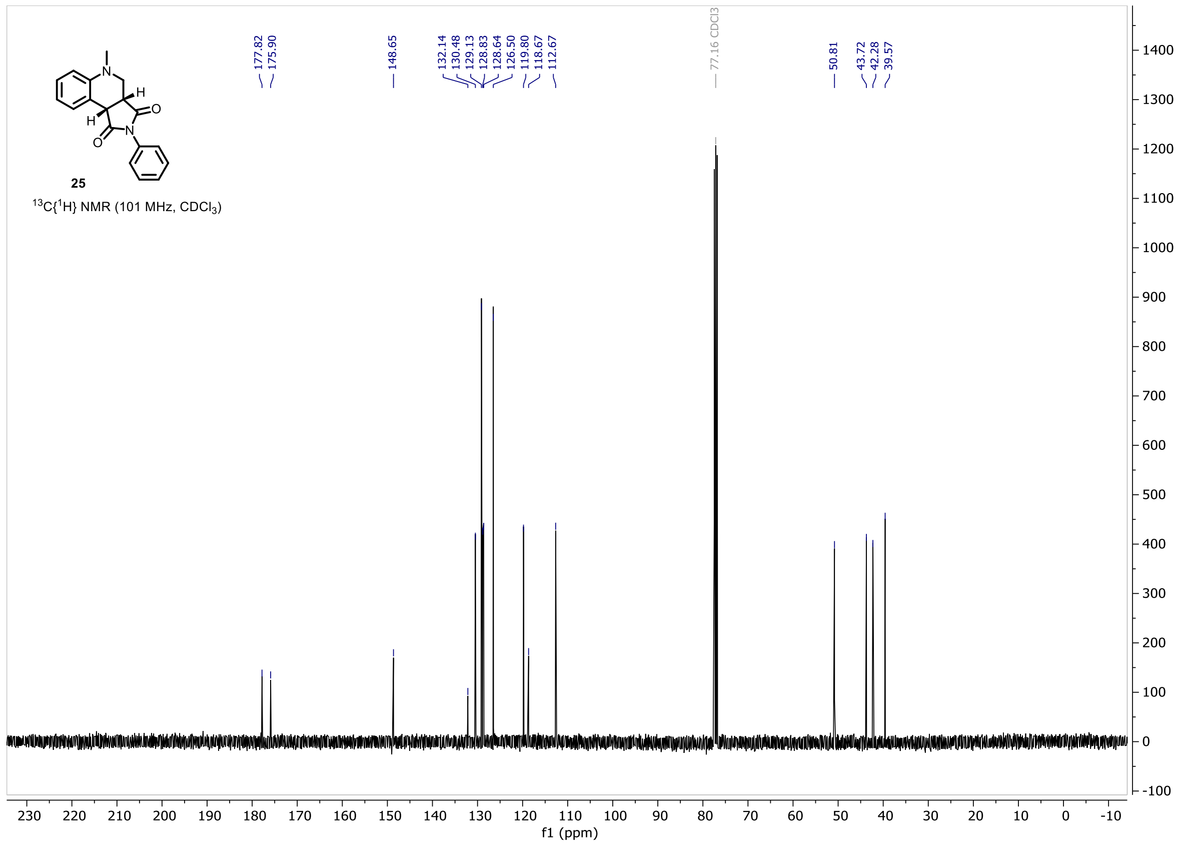

25
^{13}C{^{1}H} NMR (101 MHz, CDCl$_3$)
f1 (ppm)

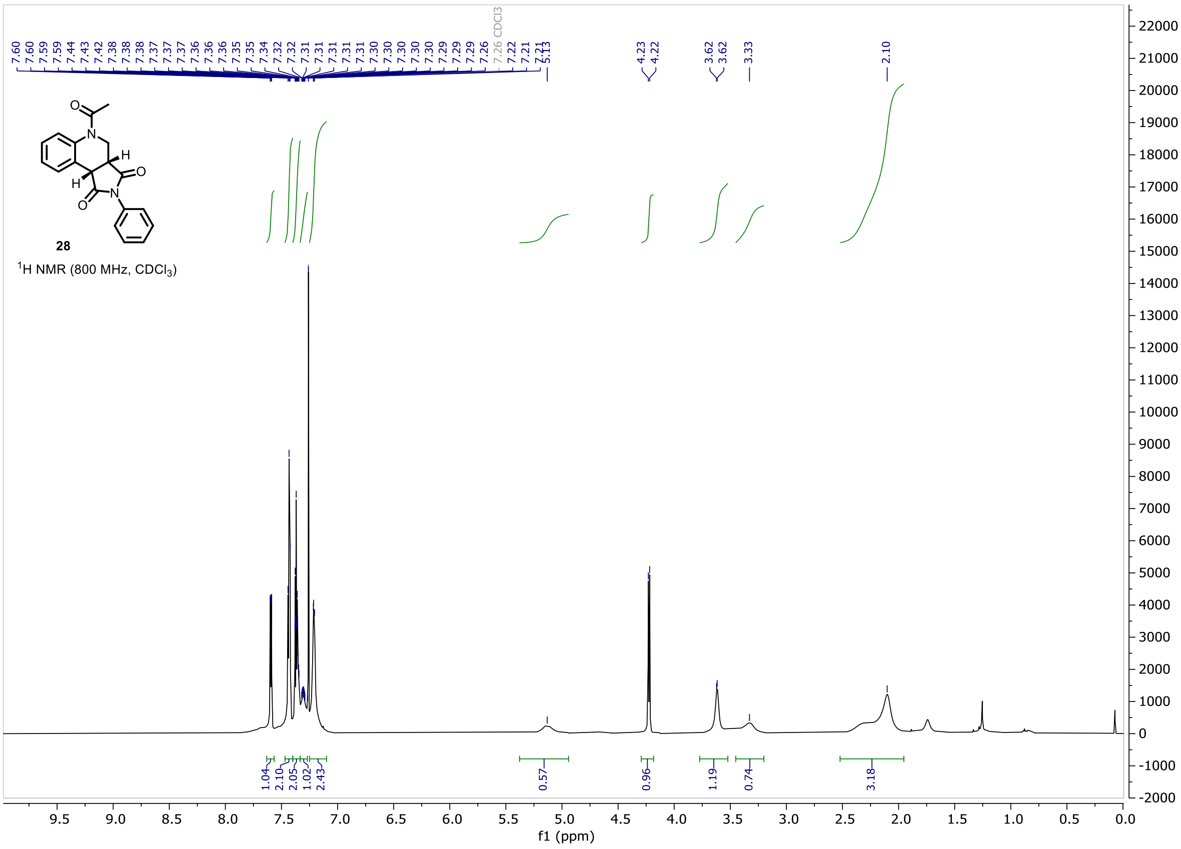
7.60 7.60 7.59 7.59 7.44 7.43 7.42 7.38 7.38 7.38 7.37 7.37 7.36 7.36 7.36 7.35 7.35 7.34 7.32 7.32 7.31 7.31 7.31 7.31 7.31 7.30 7.30 7.30 7.30 7.29 7.29 7.29 7.26 7.26 CDCl3 7.22 7.21 7.21 5.13
4.23 4.22
3.62 3.62
3.33
2.10
28
1H NMR (800 MHz, CDCl3)
1.04 2.10 2.05 1.02 2.43
0.57
0.96
1.19
0.74
3.18
f1 (ppm)

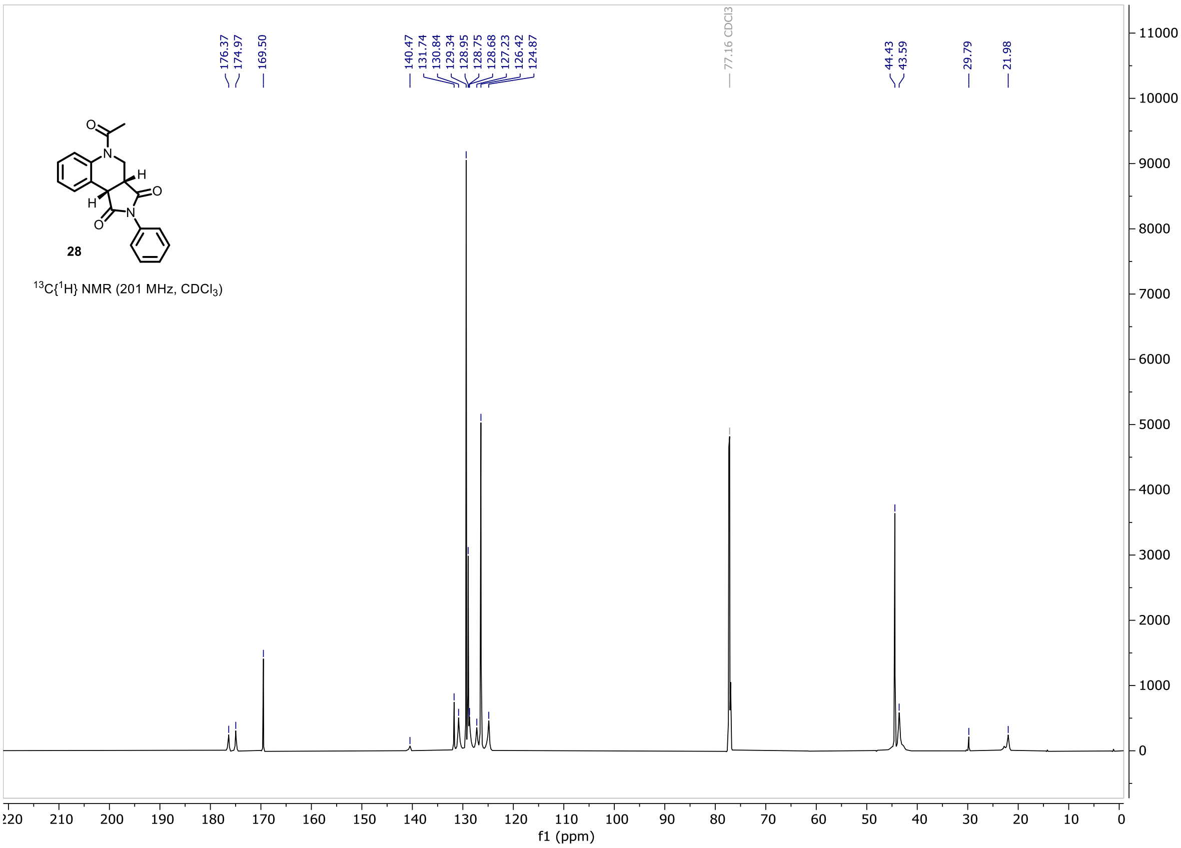
176.37 174.97
169.50
140.47 131.74 130.84 129.34 128.95 128.75 128.68 127.23 126.42 124.87
77.16 CDCl3
44.43 43.59
29.79
21.98
28
13C{1H} NMR (201 MHz, CDCl3)
f1 (ppm)

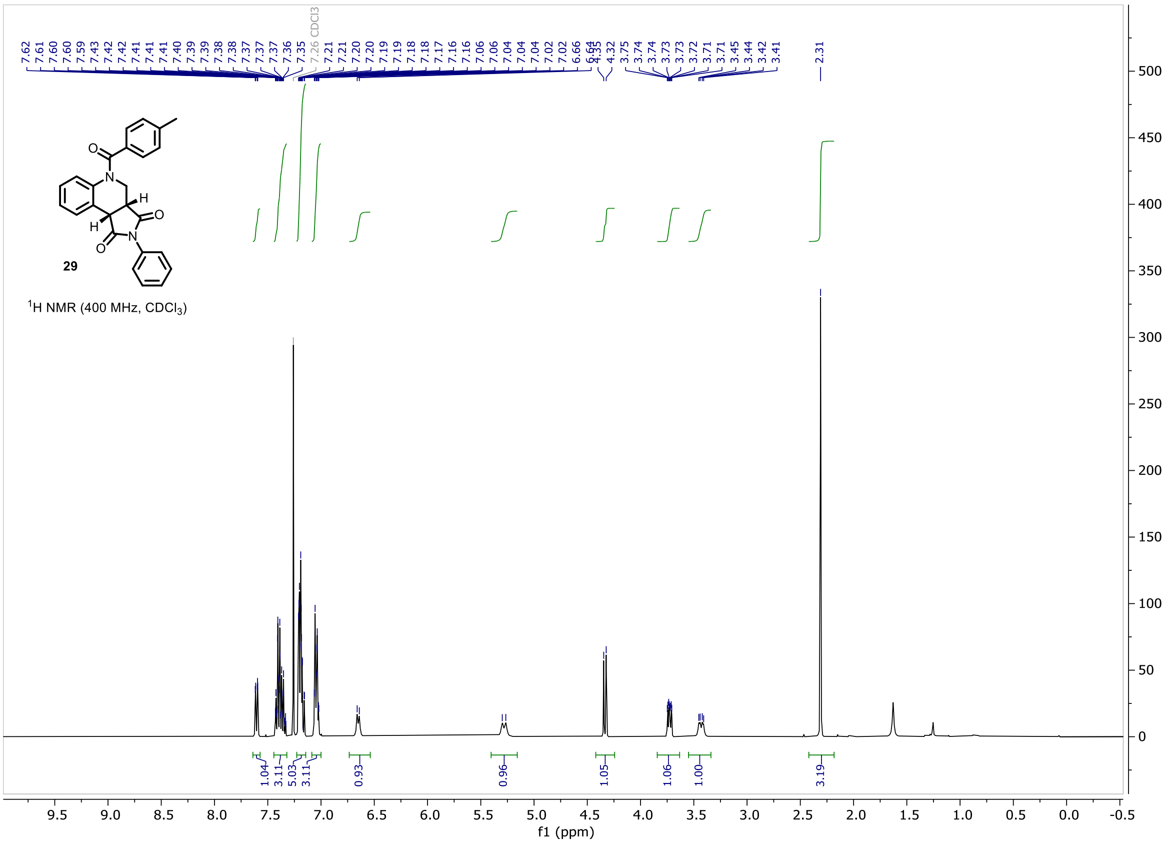
29
^{1}H NMR (400 MHz, CDCl$_3$)
7.26 CDCl3
2.31
f1 (ppm)

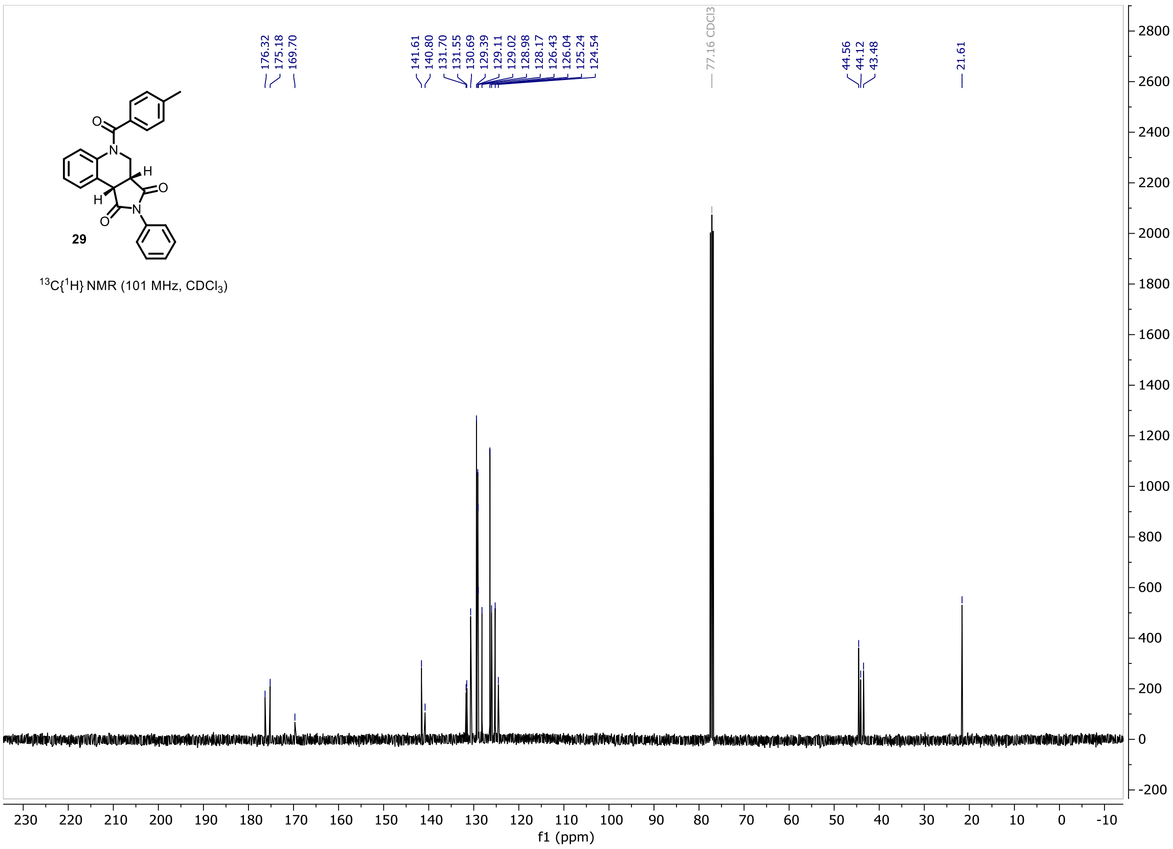
29
^{13}C{^{1}H} NMR (101 MHz, CDCl$_3$)
176.32
175.18
169.70
141.61
140.80
131.70
131.55
130.69
129.39
129.11
129.02
128.98
128.17
126.43
126.04
125.24
124.54
77.16 CDCl3
44.56
44.12
43.48
21.61
f1 (ppm)

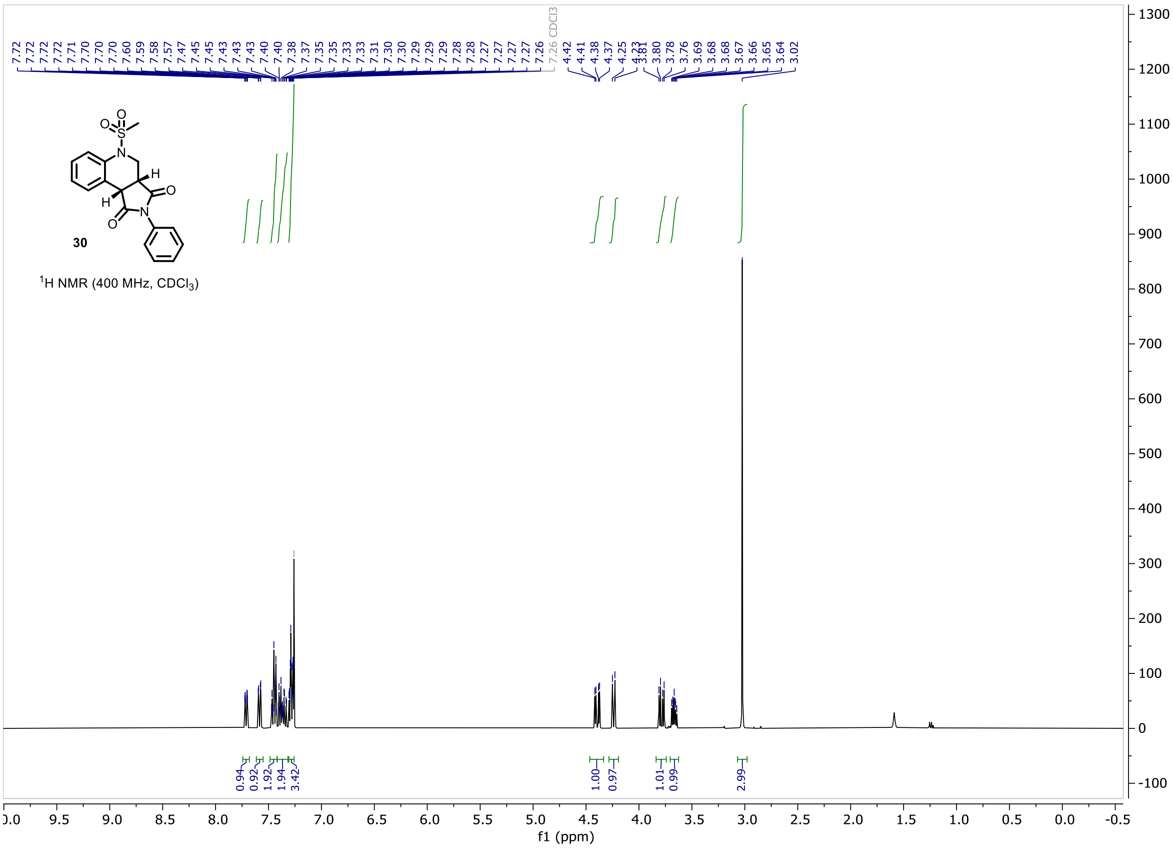

7.72 7.72 7.72 7.72 7.71 7.70 7.70 7.70 7.60 7.59 7.58 7.57 7.47 7.45 7.45 7.43 7.43 7.43 7.40 7.38 7.37 7.35 7.35 7.33 7.33 7.31 7.30 7.29 7.29 7.29 7.28 7.28 7.27 7.27 7.27 7.27 7.26
7.26 CDCl3
4.42 4.41 4.38 4.37 4.25 4.23 3.81 3.80 3.78 3.76 3.69 3.68 3.68 3.67 3.66 3.65 3.64 3.02
30
1H NMR (400 MHz, CDCl3)
0.94 0.92 1.92 1.94 3.42 1.00 0.97 1.01 0.99 2.99
f1 (ppm)

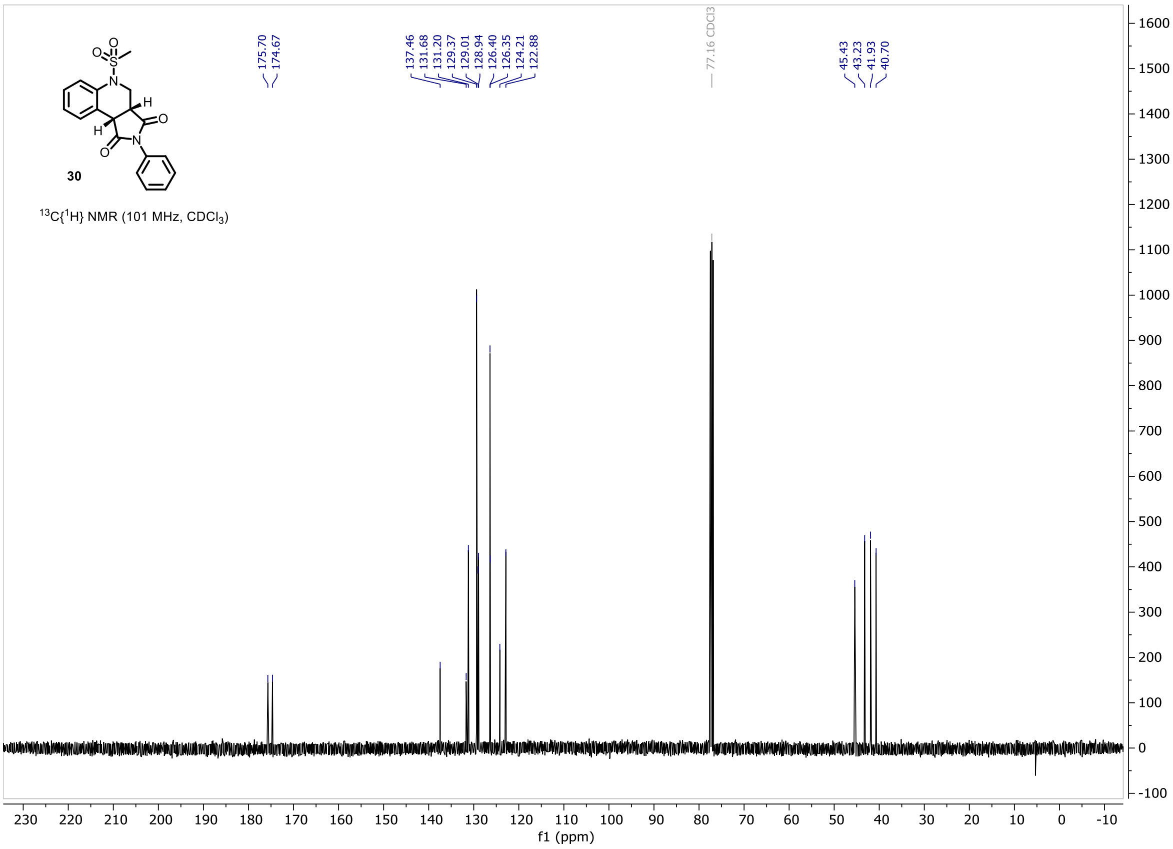

175.70 174.67
137.46 131.68 131.20 129.37 129.01 128.94 126.40 126.35 124.21 122.88
77.16 CDCl3
45.43 43.23 41.93 40.70
30
13C{1H} NMR (101 MHz, CDCl3)
f1 (ppm)

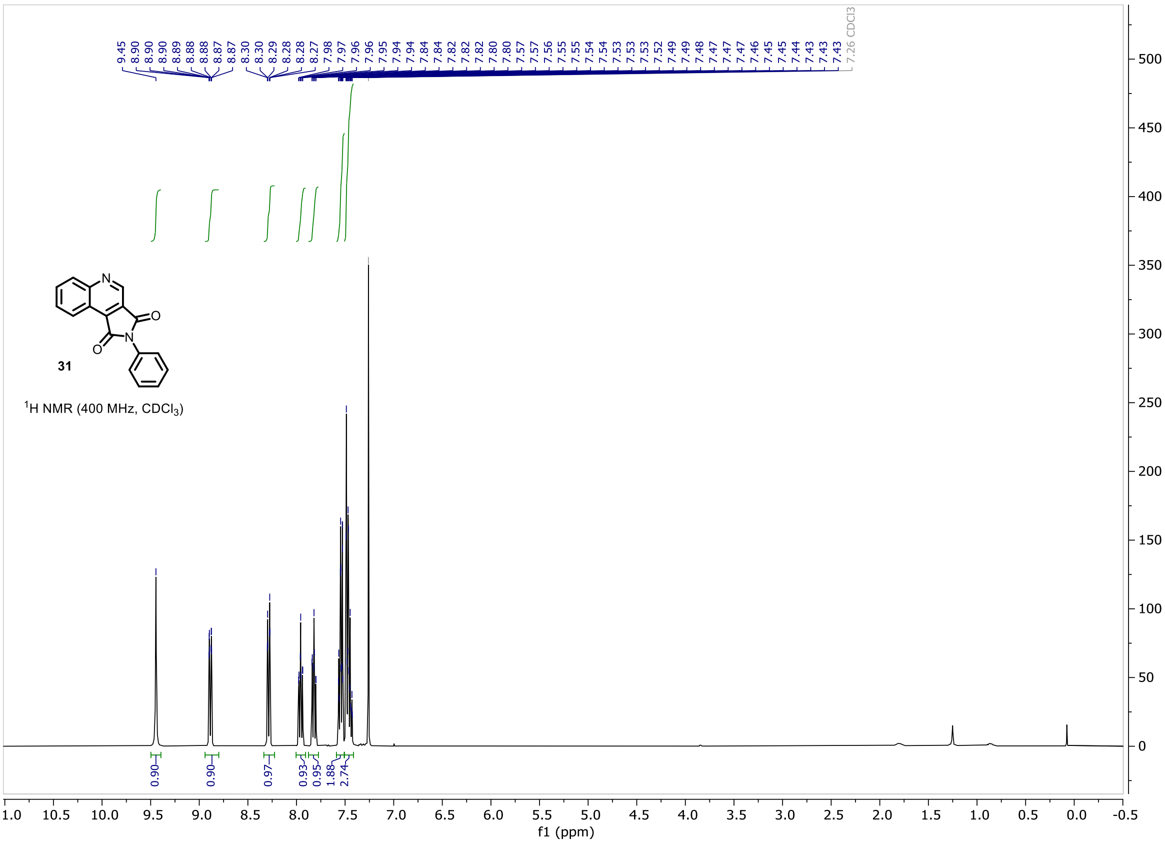
31
^{1}H NMR (400 MHz, CDCl$_3$)
f1 (ppm)
7.26 CDCl3

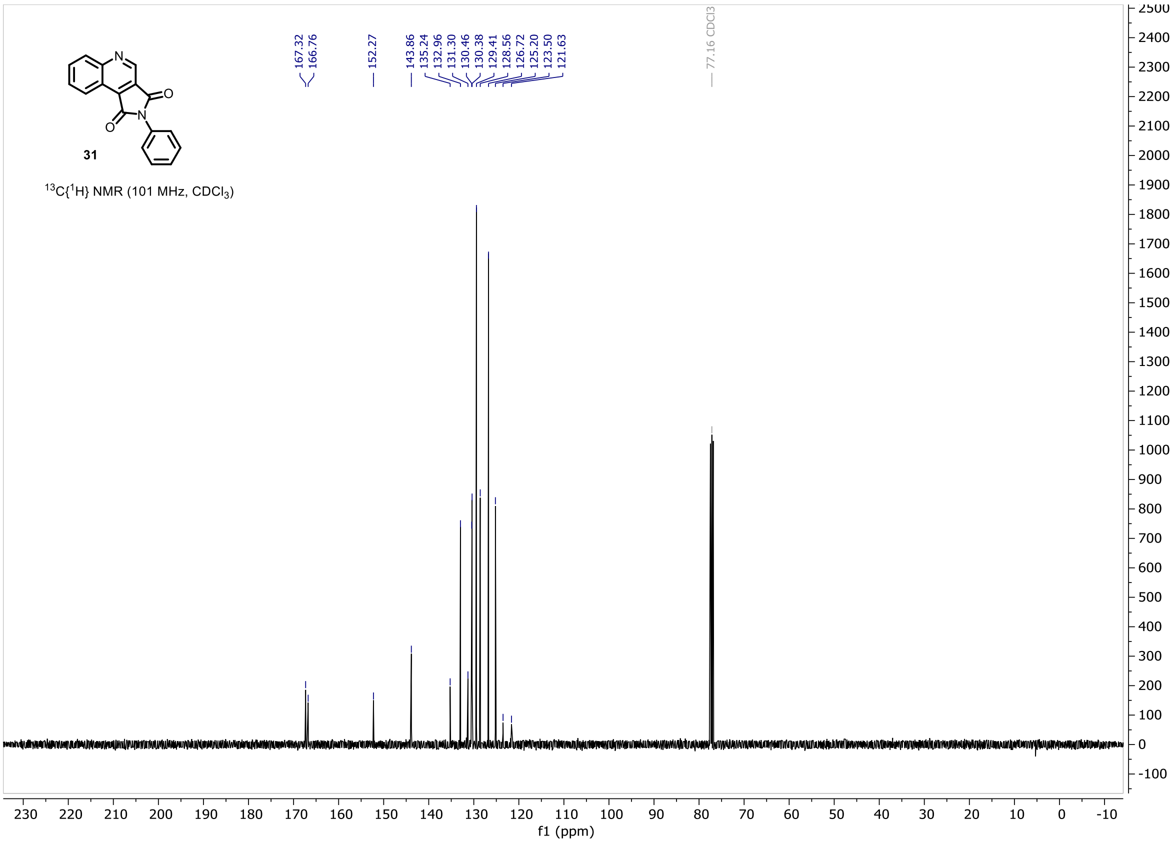
31
^{13}C{^{1}H} NMR (101 MHz, CDCl$_3$)
f1 (ppm)
77.16 CDCl3

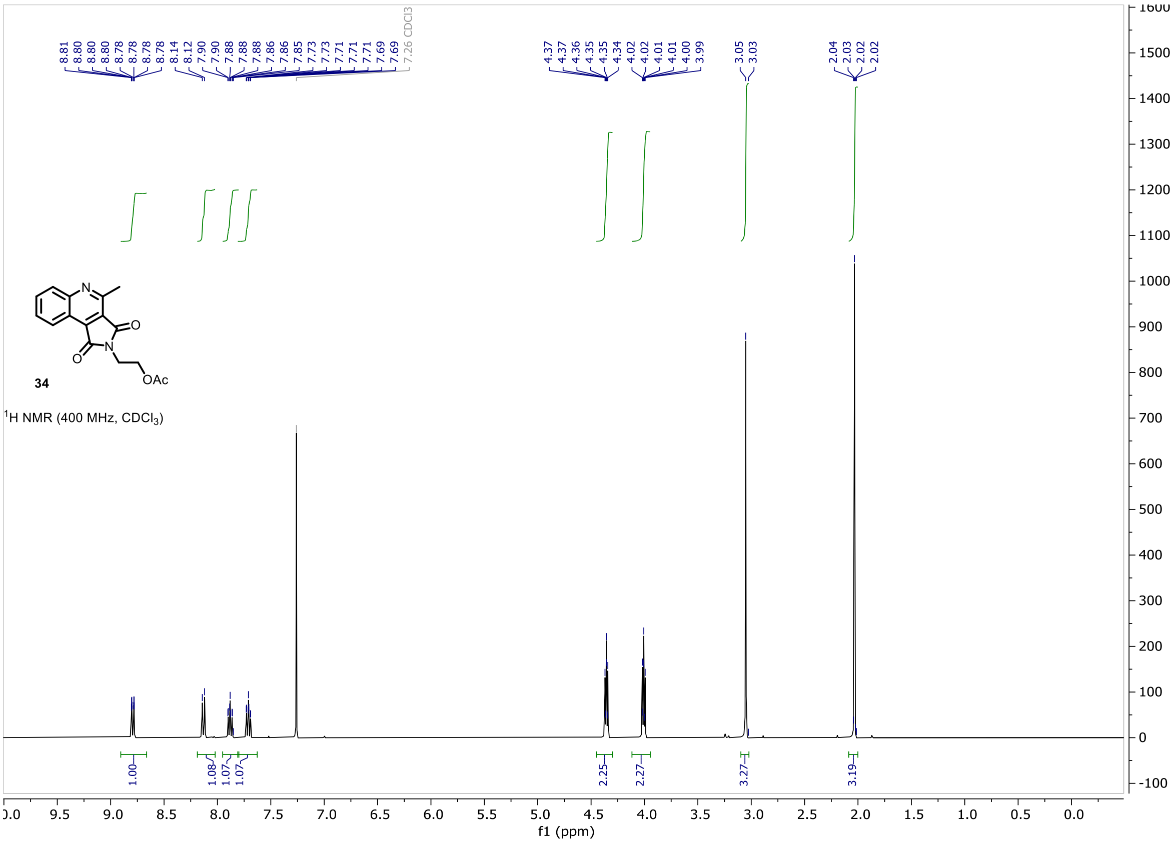
34
OAc
^{1}H NMR (400 MHz, CDCl3)
8.81 8.80 8.80 8.78 8.78 8.78 8.14 8.12 7.90 7.90 7.88 7.88 7.88 7.86 7.85 7.73 7.73 7.71 7.71 7.69 7.69
7.26 CDCl3
4.37 4.37 4.36 4.35 4.35 4.34 4.02 4.02 4.01 4.01 4.00 3.99
3.05 3.03
2.04 2.03 2.02 2.02
1.00 1.08 1.07 1.07 2.25 2.27 3.27 3.19
f1 (ppm)

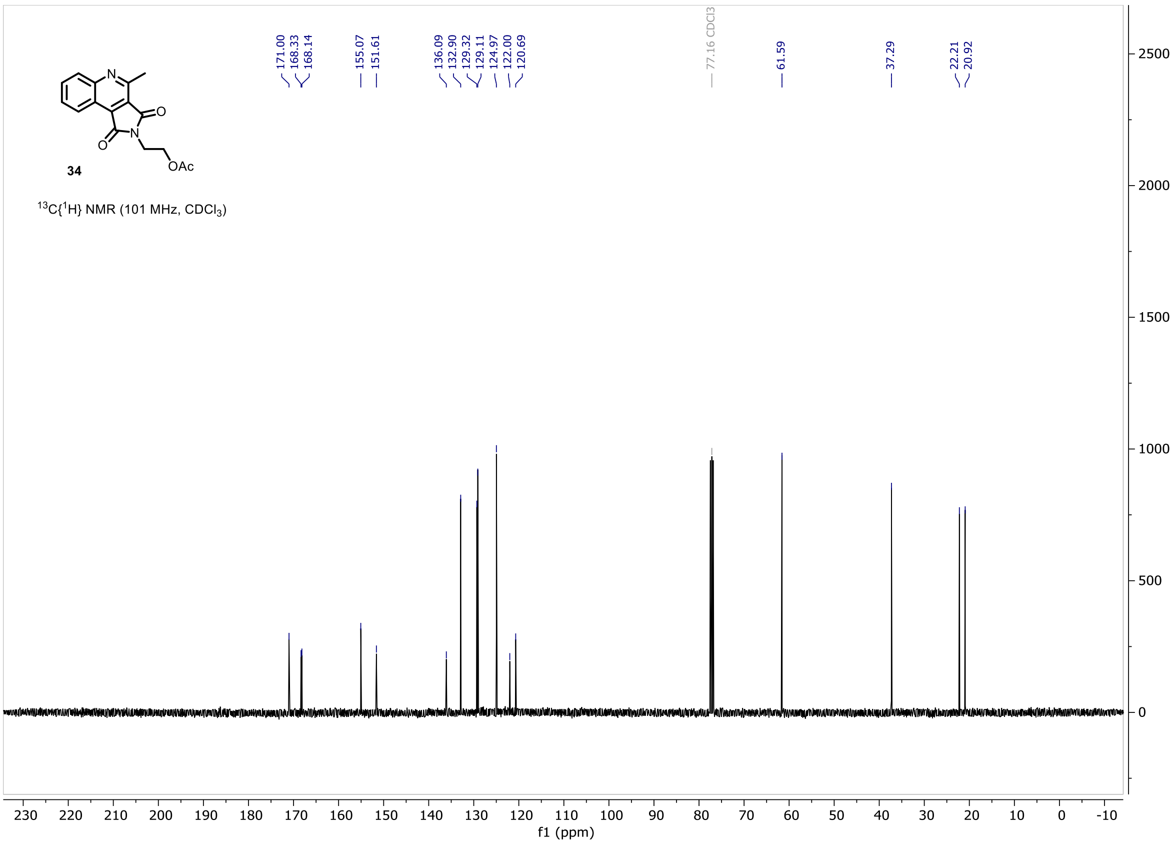
34
OAc
^{13}C{^{1}H} NMR (101 MHz, CDCl3)
171.00 168.33 168.14 155.07 151.61 136.09 132.90 129.32 129.11 124.97 122.00 120.69
77.16 CDCl3
61.59 37.29 22.21 20.92
f1 (ppm)
